# Antibiotic Prophylaxis for the Prevention of Urinary Tract Infections in Children: Guideline and Recommendations from the Emilia-Romagna Pediatric Urinary Tract Infections (UTI-Ped-ER) Study Group

**DOI:** 10.3390/antibiotics12061040

**Published:** 2023-06-12

**Authors:** Giovanni Autore, Luca Bernardi, Filippo Ghidini, Claudio La Scola, Alberto Berardi, Giacomo Biasucci, Federico Marchetti, Andrea Pasini, Maria Elena Capra, Claudia Castellini, Vera Cioni, Sante Cantatore, Andrea Cella, Francesca Cusenza, Alessandro De Fanti, Elisa Della Casa Muttini, Margherita Di Costanzo, Alessandra Dozza, Claudia Gatti, Cristina Malaventura, Luca Pierantoni, Giovanni Parente, Gabriella Pelusi, Serafina Perrone, Laura Serra, Francesco Torcetta, Enrico Valletta, Gianluca Vergine, Francesco Antodaro, Andrea Bergomi, Jennifer Chiarlolanza, Laura Leoni, Franco Mazzini, Roberto Sacchetti, Agnese Suppiej, Lorenzo Iughetti, Andrea Pession, Mario Lima, Susanna Esposito, The UTI-Ped-ER Study Group

**Affiliations:** 1 Pediatric Clinic, University Hospital, Department of Medicine and Surgery, University of Parma, 43126 Parma, Italy; giovanniautore@gmail.com (G.A.); bernardi.luca.91@gmail.com (L.B.); francescacusenza7@gmail.com (F.C.); 2 Pediatric Surgery, University of Modena and Reggio Emilia, 41125 Modena, Italy; ghidini.filippo@aou.mo.it; 3 Pediatric Clinic, IRCCS Azienda Ospedaliera Universitaria di Bologna, 40138 Bologna, Italy; clasc1976@gmail.com (C.L.S.); andrea.pasini@aosp.bo.it (A.P.); andrea.pession@unibo.it (A.P.); 4 Neonatology and Neonatal Intensive Care Unit, Department of Medical and Surgical Sciences of Mothers, Children and Adults, University of Modena and Reggio Emilia, 41125 Modena, Italy; alberto.berardi@unimore.it (A.B.); dellacasa.elisa@aou.mo.it (E.D.C.M.); 5 Pediatrics and Neonatology Unit, Guglielmo da Saliceto Hospital, 29122 Piacenza, Italy; giacomo.biasucci@unipr.it (G.B.); m.capra@ausl.pc.it (M.E.C.); a.cella@ausl.pc.it (A.C.); m.dicostanzo@ausl.pc.it (M.D.C.); 6 Pediatrics and Neonatology Unit, Ravenna Hospital, AUSL Romagna, 48121 Ravenna, Italy; federico.marchetti@auslromagna.it; 7 Pediatric Unit, Carpi Hospital, AUSL Modena, 41012 Carpi, Italy; c.castellini@ausl.mo.it (C.C.); f.torcetta@ausl.mo.it (F.T.); 8 Pediatric Unit, Sassuolo Hospital, AUSL Modena, 41049 Sassuolo, Italy; v.cioni@ospedalesassuolo.it; 9 Pediatrics Unit, Department of Medical and Surgical Sciences of Mothers, Children and Adults, University of Modena and Reggio Emilia, 41125 Modena, Italy; cantadore.sante@aou.mo.it (S.C.); lorenzo.iughetti@unimore.it (L.I.); 10 Pediatrics Unit, Santa Maria Nuova Hospital, AUSL-IRCCS of Reggio Emilia, 42123 Reggio Emilia, Italy; alessandro.defanti@ausl.re.it; 11 Pediatric Unit, Pavullo Hospital, AUSL Modena, 41026 Pavullo, Italy; a.dozza@ausl.modena.it; 12 Pediatric Surgery, University Hospital, 43126 Parma, Italy; clgatti@ao.pr.it; 13 Pediatric Clinic, University of Ferrara, 44124 Ferrara, Italy; mlvcst@unife.it (C.M.); agnese.suppiej@unife.it (A.S.); 14 Pediatric Emergency Unit, IRCCS Azienda Ospedaliera Universitaria di Bologna, 40138 Bologna, Italy; luca.pierantoni@aosp.bo.it; 15 Pediatric Surgery, IRCCS Azienda Ospedaliera Universitaria di Bologna, 40138 Bologna, Italy; giovanni.parente@outlook.com (G.P.); mario.lima@unibo.it (M.L.); 16 Pediatrics Surgery, Rimini Hospital, AUSL Romagna, 47921 Rimini, Italy; gabriella.pelusi2@auslromagna.it; 17 Neonatology Unit, University Hospital, Department of Medicine and Surgery, University of Parma, 43126 Parma, Italy; serafina.perrone@unipr.it; 18 Pediatric Unit, Imola Hospital, AUSL Imola, 40026 Imola, Italy; l.serra@ausl.imola.bo.it; 19 Pediatric Unit, Forlì Hospital, AUSL Romagna, 47122 Forlì, Italy; enrico.valletta@auslromagna.it; 20 Pediatric Clinic, Rimini Hospital, AUSL Romagna, 47921 Rimini, Italy; gianluca.vergine@auslromagna.it; 21 Primary Care Pediatrician, AUSL Modena, 41125 Modena, Italy; francesco.antodaro@gmail.com (F.A.); nao.bergomi@gmail.com (A.B.); jenniferchiarolanza@gmail.com (J.C.); 22 Primary Care Pediatrician, AUSL Parma, 43126 Parma, Italy; lauraleoni19@gmail.com; 23 Primary Care Pediatrician, AUSL Romagna, 47521 Cesena, Italy; franco.mazzini@medici.progetto-sole.it; 24 Primary Care Pediatrician, AUSL Piacenza, 29121 Piacenza, Italy; robertosacchetti16@gmail.com

**Keywords:** antibiotic prophylaxis, antibiotic resistance, obstructive uropathies, pediatrics, urinary tract infection

## Abstract

*Background:* Urinary tract infection (UTI) represents one of the most common infectious diseases and a major cause of antibiotic prescription in children. To prevent recurrent infections and long-term complications, low-dose continuous antibiotic prophylaxis (CAP) has been used. However, the efficacy of CAP is controversial. The aim of this document was to develop updated guidelines on the efficacy and safety of CAP to prevent pediatric UTIs. *Methods:* A panel of experts on pediatric infectious diseases, pediatric nephrology, pediatric urology, and primary care was asked clinical questions concerning the role of CAP in preventing UTIs in children. Overall, 15 clinical questions were addressed, and the search strategy included accessing electronic databases and a manual search of gray literature published in the last 25 years. After data extraction and narrative synthesis of results, recommendations were developed using the Grading of Recommendations, Assessment, Development, and Evaluations (GRADE) methodology. *Results:* The use of CAP is not recommended in children with a previous UTI, with recurrent UTIs, with vesicoureteral reflux (VUR) of any grade, with isolated hydronephrosis, and with neurogenic bladder. CAP is suggested in children with significant obstructive uropathies until surgical correction. Close surveillance based on early diagnosis of UTI episodes and prompt antibiotic therapy is proposed for conditions in which CAP is not recommended. *Conclusions:* Our systematic review shows that CAP plays a limited role in preventing recurrences of UTI in children and has no effect on its complications. On the other hand, the emergence of new antimicrobial resistances is a proven risk.

## 1. Introduction

Urinary tract infection (UTI) represents one of the most common infectious diseases and a major cause of antibiotic prescription in the pediatric population [1]. UTIs affect up to 2.8% of children annually in high-income countries, with recurrence rates ranging from 8% to 30% [2,3]. Risk factors for recurrent UTIs in pediatric age include congenital abnormalities in the urinary tract (i.e., vesicoureteral reflux (VUR), ureteropelvic junction obstruction, urethral valves), chronic constipation, voiding bladder dysfunction or incomplete bladder emptying, neurogenic bladder, gender, and poor toilet hygiene [2,3]. Even though acute septic complications are uncommon, permanent renal scarring may occur in 15% of first episodes of UTI and in 40% of total cases [4,5]. The loss of renal function may lead to proteinuria, hypertension, and kidney failure. To prevent these frightening complications and UTI recurrences, low-dose continuous antibiotic prophylaxis (CAP) has been used, particularly in children with urinary tract anomalies [3,4,5]. However, the efficacy of CAP is still controversial. Recent high-quality evidence reported a small benefit from CAP only in terms of prevention of recurrence and only in some subgroups of patients, without any effect on the risk of renal scarring [6].

On the other hand, misuse of antibiotics is known to increase the spread of antimicrobial resistance in community-acquired UTIs, which is already an alarming threat further limiting the effectiveness of available antibiotics. In a European study that included Italy, resistance rates to amoxicillin and trimethoprim–sulfamethoxazole were found to already exceed 50% in some populations of both outpatients and inpatients [7]. In a retrospective study conducted in the USA, 368,398 isolates from children with UTI were analyzed, and it was observed that 1.97% were resistant to third-generation cephalosporins and 0.47% were identified as extended-spectrum beta-lactamase (ESBL) producers, with both phenotypes increasing during the study period [8]. In an Italian population of hospitalized children with febrile UTI, resistance to amoxicillin/clavulanic acid was observed in 33.8% of cases, and infections caused by MDR pathogens represented 6.7% of cases [9].

Guidelines show uncertainty on which group of patients can really benefit from CAP. Available recommendations concern almost exclusively patients with VUR, and they are often based on expert opinions. In addition, there is no strong evidence to support individual drugs, dosages, and duration of antibiotic prophylaxis. Furthermore, the current evidence is biased by the heterogeneity in the classification and management of pediatric uropathies. This might undermine the gathering of strong evidence and the design of specific trials.

We previously performed a pilot study investigating the agreement among a panel of experts on the diagnosis and management of pediatric UTI through the Delphi method. The consensus document suggested antibiotic prophylaxis only in children with high-grade VUR or a history of recurrent UTIs until the exclusion of urological anomalies through voiding cystourethrography (VCUG) [10]. The study underlined the need for a more in-depth analysis of available evidence about CAP for pediatric UTIs. The aim of this document was to develop updated guidelines on the efficacy and safety of CAP to prevent pediatric UTIs.

## 2. Materials and Methods

This systematic review was conducted in accordance with the Preferred Reporting Items for Systematic Reviews and Meta-Analyses (PRISMA) statement [11]. A pre-specified protocol was prospectively registered in PROSPERO under registration number CRD42023411459 (available at http://www.crd.york.ac.uk/PROSPERO, accessed on 30 May 2023).

### 2.1. Clinical Question

A scientific board composed of experts on pediatric infectious diseases and primary care was in charge of designing and conducting this systematic review. A panel of experts on pediatric infectious diseases, pediatric nephrology, pediatric urology, and primary care was selected by the scientific board based on their clinical experience and research skills and was asked to develop clinical questions concerning the role of CAP in preventing UTIs in children. The panel voted and validated 15 clinical questions evaluating the efficacy and safety of CAP in subgroups of children with specific conditions at risk of UTI and investigating its optimal duration and dosage. According to validated clinical questions, populations, interventions, comparisons, and outcomes (PICO) were addressed by the scientific board and defined as follows:

- Populations included pediatric patients aged under 18 years with any of the following conditions: a previous UTI, history of recurrent UTIs, VUR, isolated hydronephrosis, infravesical obstruction, primary obstructive megaureter, or neurogenic bladder. Populations also included pediatric patients aged under 18 years undergoing pyeloplasty, ablation of urethral valves, ureteral reimplantation, or endoscopic treatment of VUR.
- Interventions and comparisons included CAP versus no prophylaxis or placebo, different antibiotics, different dosages, continuing versus discontinuing CAP after surgical or endoscopic treatments, and confirming versus changing antibiotics after a breakthrough infection.
- Outcomes were risk of UTI recurrences, risk of new renal scarring, risk of new antimicrobial resistances, and risk of drug-related adverse events.

### 2.2. Search Strategy and Eligibility Criteria

The search strategy included accessing electronic databases (MEDLINE, EMBASE, Scopus, and Cochrane Library) through ad hoc search queries and a manual search of gray literature consulting guideline-focused repositories (Web of Science, Google Scholar, and National Institute for Health and Care Excellence) and documents from scientific societies. Moreover, the references of included articles were further evaluated to identify other relevant documents for inclusion. The references were regularly updated during the drafting of the review. The search strategy is detailed in the Supplementary Materials.

Each record was independently screened by two members of the scientific board, and disagreements were resolved through consensus among the whole board. In the case of studies reporting results in more than one publication, the most recent and comprehensive article was included. Observational studies, randomized clinical trials, systematic reviews with or without meta-analysis, and guidelines published from January 1997 to January 2023 were considered for inclusion. Case reports, case series, editorials, and clinical studies from middle- and low-income countries were excluded. Articles published in languages other than English or Italian were also excluded. Studies excluded after full-text evaluation were documented, and reasons for exclusion are detailed in the Supplementary Material S1.

### 2.3. Risk of Bias and Methodological Quality Assessment

Each included article was independently evaluated by at least two members of the scientific board. The risk of bias of randomized clinical trials and observational studies was evaluated using the Risk of Bias 2 (RoB2) tool and the Newcastle–Ottawa Scale (NOS), respectively [12,13]. Results from RoB2 analysis are presented as plots [14].

The methodological quality of systematic reviews and meta-analyses was assessed using the AMSTAR-2 tool [15]. Evaluation of included guidelines was independently conducted by four members of the scientific board using the AGREE II tool [16,17].

### 2.4. Data Extraction and Synthesis

Data extraction was performed independently by three reviewers. Although no meta-analysis was performed, a narrative synthesis of included studies was provided. Results for comparable outcomes from randomized clinical trials and observational studies were pooled using the GRADEpro software and presented as risk ratio (RR), odds ratio (OR), or hazard ratio (HR) with 95% confidence intervals (95% CIs) [18].

Indirectness, inconsistency, and imprecision were evaluated for pooled results of each outcome. Assessment of imprecision was based on both the optimal information size (OIS) criterion and 95% CI. OIS was estimated using the parameters suggested by Guyatt et al. (α = 0.05, β = 0.2, relative risk reduction = 25%) [19]. Certainty of pooled results was established using GRADEpro software. Results from meta-analyses were not included in pooled synthesis but were presented separately to avoid compromising their higher statistical quality.

The panel followed a systematic process that included a standardized methodology for rating the certainty of the evidence and strength of recommendation using the Grading of Recommendations, Assessment, Development, and Evaluations (GRADE) methodology. Supplementary Material S1 reports the clinical questions and PICO items in detail. Recommendations were developed for each clinical question and presented with the associated strength and evidence quality. When evidence from clinical studies was insufficient, recommendations were developed through consensus among the panel of experts and presented as expert opinions. A draft version of the document underwent extensive review by the experts.

## 3. Results

**Figure 1:**
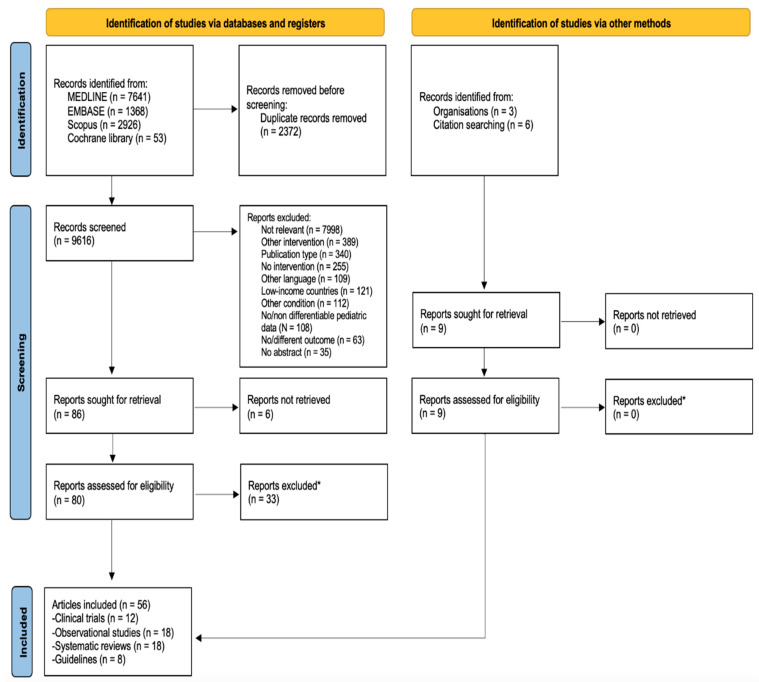
PRISMA flow diagram showing the identification, screening, and inclusion of the studies. * Full list of excluded studies with detailed description is provided in Supplementary Material S1 (Table S1).

Overall, 9616 records from electronic databases and 9 records from gray literature were screened, resulting in 89 full-text articles further assessed for eligibility. Of these articles, 6 reports were not retrieved and 33 were excluded. Fifty-six articles were finally included: 12 randomized clinical trials, 18 observational studies, 18 systematic reviews, and 8 guidelines [Figure 1]. At least one article was available for 13 of the 15 clinical questions.

### 3.1. Should Continuous Antibiotic Prophylaxis Be Used in All Children with a Previous UTI?

*Recommendation 1*

*Continuous antibiotic prophylaxis is not routinely indicated in all children after the first episode of UTI (strong recommendation against the intervention; evidence quality: moderate).*

Pooled results of three randomized clinical trials and one observational cohort study failed to demonstrate a significant benefit of CAP in all children with a previous UTI, in terms of both UTI recurrence and renal scarring. On the other hand, a significantly higher risk of drug-related adverse effects and an almost significant increase in antimicrobial resistances were observed in two of the included clinical trials [Table 1]. The trial conducted by Craig et al. was the only one reporting a statistically significant reduction in UTI recurrence in children receiving TMP-SMX (HR: 0.61; 95% CI: 0.40–0.93; *p* = 0.02) [20]. All three RCTs and the cohort study by Conway et al. showed that CAP had no effect on the risk of new renal scarring [20,21,22,23].

Children randomly assigned to CAP by Craig et al. had an almost significantly increased risk of subsequent UTIs caused by drug-resistant uropathogens (RR 1.85; 95% CI: 0.96–3.55) [20]. In the multivariate analysis of the observational cohort study by Conway et al., CAP was a significant risk factor for antimicrobial resistances (HR: 7.50; 95% CI: 1.60–35.17) [23]. An observational cross-sectional study confirmed that CAP is a significant risk factor for subsequent resistance to antibiotics commonly used for prophylaxis [24].

Drug-related adverse events were analyzed by two RCTs that reported a strong association with the use of CAP (pooled RR: 2.41; 95% CI: 1.19–4.89) [20,21] [Table 1].

**Table 1:** Pooled results and certainty assessment of randomized clinical trials and observational studies involving children with one previous UTI (first clinical question).

Certainty Assessment							No. of Patients		Effect		Certainty
No. of Studies	Study Design	Risk of Bias	Inconsistency	Indirectness	Imprecision	Other Considerations	Antibiotic Prophylaxis	No Prophylaxis	Relative (95% CI)	Absolute (95% CI)	
**Risk of UTI recurrence (follow-up: mean 12 months; assessed as rates of recurrence)**											
3 [20,21,22]	randomized trials	not serious ^a^	not serious	not serious	serious ^b^	none	68/515 (13.2%)	94/617 (15.2%)	**RR 0.87** (0.65 to 1.16)	**20 fewer per 1000** (53 fewer–24 more)	⨁⨁⨁◯ Moderate
1 [23]	observational studies	not serious ^c^	not serious	not serious	very serious ^b^	none	19/128 (14.8%)	64/483 (13.3%)	**HR 1.01** (0.50 to 2.02)	**1 more per 1000** (64 fewer–117 more)	⨁◯◯◯ Very low
**Risk of new renal scars (follow-up: mean 12 months; assessed as rates of new renal scars on DMSA scan)**											
3 [20,21,22]	randomized trials	not serious ^a^	not serious	not serious	serious ^b^	none	14/358 (3.9%)	15/309 (4.9%)	**RR 0.81** (0.40 to 1.64)	**9 fewer per 1000** (29 fewer–31 more)	⨁⨁⨁◯ Moderate
**Risk of new antimicrobial resistances (follow-up: mean 12 months; assessed as rates of infections resistant to empiric antibiotics)**											
1 [20]	randomized trials	not serious ^a^	not serious	not serious	serious ^b^	none	24/288 (8.3%)	13/288 (4.5%)	**RR 1.85** (0.96 to 3.55)	**38 more per 1000** (2 fewer–115 more)	⨁⨁⨁◯ Moderate
2 [23,25]	observational studies	not serious ^c^	not serious	not serious	serious ^d^	very strong association	24/45 (53.3%)	43/399 (10.8%)	**OR 4.49** (2.81 to 7.12)	**244 more per 1000** (146 more–355 more)	⨁⨁⨁◯ Moderate
**Risk of drug-related adverse events (follow-up: mean 12 months; assessed as rates of drug-related adverse event)**											
2 [20,21]	randomized trials	not seriou ^a^	not serious	not serious	not serious	strong association	29/499 (5.8%)	10/415 (2.4%)	**RR 2.41** (1.19 to 4.89)	**34 more per 1000** (5 more–94 more)	⨁⨁⨁⨁ High

CI: confidence interval; HR: hazard ratio; OR: odds ratio; RR: risk ratio. ^a^: complete risk of bias analysis with results for single domains of the RoB2 tool is presented in Supplementary Materials. ^b^: OIS criterion is not met and 95% CI overlaps no effect. ^c^: complete risk of bias analysis with results for Newcastle–Ottawa Scale tool is presented in Supplementary Tables and Figures. ^d^: OIS criterion is not met.

Nine systematic reviews (three high-quality, one moderate-quality, and five low-quality), including studies on children with and without VUR, agreed on the lack of benefits of CAP in all children after the first UTI [6,25,26,27,28,29,30,31]. Detailed results of all the included systematic reviews and meta-analyses are reported in Table S3 of Supplementary Materials.

Clinical guidelines published by the Italian Society for Pediatric Nephrology (SINePe) and the Kidney Health Australia/Caring for Australians and New Zealanders with Renal Impairment (KHA/CARI) and the Swiss consensus recommendations suggest against the routine use of CAP (Grade A) [32,33,34] [Table S4].

### 3.2. Should Continuous Antibiotic Prophylaxis Be Used in All Children with a History of Recurrent UTIs?

*Recommendation 2*

*A history of recurrent UTIs without underlying urological anomalies does not constitute a sufficient indication for continuous antibiotic prophylaxis (weak recommendation against the intervention; evidence quality: low).*

*Short-term prophylaxis may be considered until the exclusion of urological anomalies (weak recommendation for the intervention; expert opinion).*

Few studies evaluated the indication for CAP based only on a history of recurrent UTIs independently from the assessment of VUR or other urological abnormalities [Table 2].

A subgroup analysis on 98 children enrolled in the RCT by Craig et al. and defined only by the history of previous recurrent UTIs showed no benefits from CAP on the risk of breakthrough UTIs (*p* = 0.59; HR: 0.65, 95% CI: 0.32–1.32) [20]. A case–control study conducted on 41 cases of breakthrough UTIs in children with a history of recurrent UTIs showed a higher, although not statistically significant, risk of resistances to empiric antibiotics in children receiving CAP (40.0% vs. 25.9%, *p* > 0.30) [35]. The systematic review conducted by Alsubaie et al. showed that CAP may reduce the risk of recurrent symptomatic UTI in children who have had more than one previous UTI, but the benefit was not statistically significant (RR, 0.75; 95% CI, 0.28–1.98). A 2.5-fold higher threat of developing an antibiotic-resistant infection was observed in children receiving CAP [36]. However, the systematic review presented a low methodological quality.

According to guidelines by SINePe and NICE, antibiotic prophylaxis may be considered for children with a history of recurrent UTIs only after other management options have been unsuccessful (behavioral and personal hygiene measures, managing any triggers, and using non-antimicrobial treatments) and/or when more than three episodes occur within 12 months [32,37] [Table S7]. However, patients with these clinical criteria also require further radiological evaluations (e.g., void cystourethrography) to exclude urinary tract anomalies, as suggested by main urological guidelines [38,39]. The panel of experts concluded that during the diagnostic workup, a short bridge antibiotic prophylaxis may be considered until exclusion of obstructive uropathies.

### 3.3. Should Continuous Antibiotic Prophylaxis Be Used in All Children with VUR of Any Grade?

*Recommendation 3*

*Continuous antibiotic prophylaxis is not recommended for children with low-grade (I–II) or non-dilating VUR (strong recommendation against the intervention; quality of evidence: moderate).*

*Close surveillance based on early diagnosis (i.e., urinalysis and urine culture) and prompt antibiotic therapy in symptomatic/febrile children may be considered in children with VUR of any grade (weak recommendation; expert opinion).*

Pooled results from five randomized clinical trials showed a statistically significant reduction in UTI recurrences in children with VUR of any grade receiving CAP (pooled RR: 0.75; 95%CI: 0.62–0.90) [20,22,40,41,42] [Table 3].

The RIVUR trial was the only one reporting statistically significant results about this outcome, observing that prophylaxis reduced the risk of recurrences by 50% (HR: 0.50; 95% CI: 0.34 to 0.74), but it strongly affected the pooled results due to its large population [41]. Pooled results from two observational studies showed no benefits from CAP in terms of UTI recurrences [43,44] [Table 3]. Six systematic reviews characterized by high methodological quality confirmed the poor benefits of CAP in children with VUR of any grade [6,28,30,45,46,47] [Table S9]. In particular, Finnel et al. observed a significant effect of CAP only on asymptomatic bacteriuria [28]. De Bessa et al. reported a significant reduction in UTI recurrence only after including the results from the RIVUR trial [48]. No clinical studies or meta-analyses found a statistically significant reduction in the risk of new renal scarring in children with VUR of any grade receiving CAP [22,30,41,42,47] [Tables S8 and S9]. The RIVUR trial also investigated the risk of antimicrobial resistances due to CAP, reporting a strong association (RR: 2.78; 95%CI: 1.74–4.42) [41]. The meta-analysis conducted by Selekman et al. found that the probability of preventing a recurrent UTI while on prophylaxis is equal to risk of developing an antimicrobial-resistant UTI (NNT = 21 for both outcomes) [45]. Two Cochrane reviews concluded that CAP may reduce the risk of repeated symptomatic infections but the benefits are probably small and must be weighed against the risk of antimicrobial resistances that may be 2.5 times greater in children on antibiotics than for children on placebo or no treatment [6,47].

Guidelines from SINePe, KHA/CARI, American Academy of Pediatrics (AAP), and European Association of Urology/European Society for Pediatric Urology (EAU/ESPU) suggest against the use of CAP in children with low-grade or non-dilating VUR in absence of anatomical abnormalities of the urinary tract [32,34,38,49] [Table S10]. However, EAU/ESPU guidelines considered symptomatic toilet-trained children with low-grade VUR at moderate risk of fUTI. The impact of LUTS and bladder–bowel dysfunction (BBD) has been highlighted by the other guidelines, suggesting that CAP might be adopted in this particular group to prevent recurrent fUTI during bladder and bowel re-education. Only the guideline by the American Urology Association recommends CAP in children aged less than 1 year with VUR of any grade and a previous UTI [39].

### 3.4. Should Continuous Antibiotic Prophylaxis Be Used in All Children with High-Grade VUR (III–V)?

*Recommendation 4*

*Considering the lack of effect of the antibiotic prophylaxis on the risk of renal scarring, continuous antibiotic prophylaxis is not routinely recommended in children with high-grade (III–IV) or dilating VUR (weak recommendation against the intervention; quality of evidence: moderate).*

*Close surveillance based on early diagnosis (i.e., urinalysis and urine culture) and prompt antibiotic therapy in symptomatic/febrile children is recommended in children with VUR of any grade (weak recommendation; expert opinion).*

Pooled results from two randomized clinical trials showed a statistically significant reduction in UTI recurrences in children with high-grade VUR receiving CAP (pooled RR: 0.49; 95%CI: 0.30–0.81) [20,50,51] [Table 4].

The statistical significance of the results about UTI recurrence is due to data from the Swedish reflux trial that was characterized by a non-blind design and a serious risk of imprecision due to limited sample size [50,51]. The effect of CAP on the risk of new renal scarring was assessed only by the Swedish reflux trial and was found to be not statistically significant [51]. The meta-analysis conducted by de Bessa et al. confirmed that children with high-grade VUR receiving prophylaxis present a lower risk of UTI recurrence (absolute risk reduction was 8.23%; NNT was 12.15; *p* = 0.008) [48]. The risk of new antimicrobial resistances and the risk of drug-related adverse events were not analyzed by included studies.

The guidelines developed by SINePe, EAU/ESPU, and KHA/CARI and the Asian guideline suggest that CAP may be considered in children with high-grade or dilating VUR [32,34,38,52] [Table S13]. Moreover, the results of a cohort based on RIVUR and CUTIE studies proved that a top-down diagnostic approach significantly decreased the need for CAP without a clinically relevant increase in occurrence of fUTI [38]. This approach is another option that could reduce the administration of antibiotics in this group of patients. For this reason, the grade of these recommendations ranged from C to A, and EAU/ESPU points out the risk of increased antimicrobial resistance associated with CAP [38].

### 3.5. Should Antibiotic Prophylaxis Be Used in Children with Isolated Hydronephrosis?

*Recommendation 5*

*Continuous antibiotic prophylaxis is not routinely recommended in children with isolated antenatal or postnatal hydronephrosis or ureteropelvic junction obstruction (weak recommendation against the intervention; quality of evidence: low).*

The results from two observational studies failed to prove a decreased risk of UTI in children with antenatal isolated hydronephrosis receiving CAP [53,54] [Table 5].

Certainty of these results was very low due to significant inconsistency and imprecision. Multivariate analysis conducted by Herz et al. showed that only ureteral obstruction at the ureterovesical junction, ureteral dilation > 11 mm, and high-grade VUR were independent risk factors for febrile UTI [54].

A systematic review conducted by EAU/ESPU concluded that it is not possible to establish whether prophylaxis is superior to no prophylaxis in terms of decreasing UTI incidence. No conclusion could be drawn for the impact of VUR and no VUR and comparison of the different degrees of VUR because of a lack of data [55]. On the other hand, EAU/ESPU guidelines do not recommend routine screening for VUR in every child with isolated hydronephrosis [38]. Even though VUR is present in 25% of infants with isolated hydronephrosis, its clinical impact might be questionable. The studies and systematic reviews analyzed for this clinical question included children with hydronephrosis diagnosed antenatally or during the first months of life.

### 3.6. Should Antibiotic Prophylaxis Be Used in Children with Intravesical Obstructions (i.e., Urethral Valves)?

*Recommendation 6*

*There is no sufficient evidence to define efficacy and safety of continuous antibiotic prophylaxis in children with infravesical obstructions.*

*Continuous antibiotic prophylaxis may be considered until surgical correction (weak recommendation for the intervention; expert opinion).*

No clinical studies or systematic reviews investigating the role of CAP in children with urethral valves met the eligibility criteria. Since the urethral obstruction directly influences the bladder function, the evidence about this rare malformation might be derived from the management of other bladder dysfunctions. Updated guidelines published by EAU/ESPU suggest CAP in children with obstructive uropathies at risk of renal damage [38].

### 3.7. Should Antibiotic Prophylaxis Be Used in Children with Hydroureteronephrosis (i.e., Primary Obstructive Megaureter)?

*Recommendation 7*

*Continuous antibiotic prophylaxis may be considered in children with hydroureteronephrosis and ureteral dilation > 7 mm or primary obstructive megaureter (weak recommendation for the intervention; quality of evidence: low).*

Three observational studies conducted on children with primary obstructive megaureter showed that the risk of UTI was significantly lower in children receiving CAP (pooled RR: 0.49; 95% CI 0.35–0.69) [56,57,58] [Table 6].

The prospective study conducted for the Society of Fetal Urology prenatal hydronephrosis registry by Holzman et al. on 237 children reported that ureteral dilation > 7 mm was an independent risk factor for UTI, even after adjusting for VUR [57]. Gimpel et al. observed that CAP was particularly effective in the first 6 months of life, where an 83% reduction in UTI rate was found [56].

The systematic review and meta-analysis conducted by Rohner et al. concluded that the use of CAP should be taken into consideration for children with primary megaureter selected for primary non-surgical treatment. The calculated number NNT to prevent one single febrile UTI over the course of 1–2 years was 4.3 [59]. No included studies analyzed the effect of CAP on the risk of renal scarring and antimicrobial resistance.

### 3.8. Should Antibiotic Prophylaxis Be Used in Children with Neurogenic Bladder?

*Recommendation 8*

*Continuous antibiotic prophylaxis is not routinely recommended in children affected by neurogenic bladder (weak recommendation against the intervention; quality of evidence: low).*

*Proper execution of clean intermittent catheterization and close surveillance, based on early diagnosis (i.e., urinalysis and urine culture) and prompt antibiotic therapy in symptomatic/febrile children, may be considered in children with neurogenic bladder (weak recommendation; expert opinion).*

Two randomized clinical trials investigated the effect of CAP in terms of UTI recurrence in children with spina bifida undergoing clean intermittent catheterization. Pooled results showed a significant increase in UTI recurrences in children receiving CAP (pooled RR: 2.91 95% CI: 1.29–6.53) [Table 7].

This finding was particularly influenced by the clinical trial conducted by Clarke et al. [60]. On the other hand, Zegers et al. observed that discontinuation of CAP resulted in higher rates of UTI; however, the trial was characterized by very few events (six UTIs) and a high risk of bias [61]. An outdated crossover randomized clinical trial reported that CAP with nitrofurantoin significantly reduced the risk of UTI recurrence in patients with neurogenic bladder; however, the study enrolled only 15 children [62]. A large observational study conducted on 121 children with neurogenic bladder showed no significant differences in terms of UTI recurrence between children receiving or not receiving CAP [63]. In the randomized clinical trial by Zegers et al., CAP was associated with a significantly increased risk of antimicrobial resistances (RR: 1.57; 95% CI: 1.31–1.89) [64]. Two systematic reviews, although characterized by low methodological quality, showed no significant benefits from CAP in terms of UTI recurrence [29,31] [Table S20]. No studies investigated the effect of CAP on the risk of new renal scarring in children with neurogenic bladder.

The guideline published by EAU/ESPU considered only intravesical application of gentamicin but not systemic prophylaxis in patients with incomplete emptying of the bladder undergoing appropriately performed clean intermittent catheterization, but still suffering from recurrent UTIs [38].

### 3.9. Which Antibiotic Should Be Preferred for Long-Term Prophylaxis of UTI in Children?

*Recommendation 9*

*There is insufficient evidence to recommend trimethoprim–sulfamethoxazole rather than nitrofurantoin as the first-choice prophylactic antibiotic.*

*There is no evidence on the efficacy and safety of amoxicillin-clavulanic acid as a prophylactic antibiotic to prevent UTIs.*

*The prophylactic use of oral cephalosporins is not suggested due to the high risk of new antimicrobial resistances (weak recommendation against the intervention; quality of evidence: low).*

A randomized crossover trial and a large retrospective study failed to prove significant differences between the prophylactic use of trimethoprim/sulfamethoxazole or oral cephalosporins in terms of UTI recurrence [65,66] [Table 8].

However, the retrospective study indicated that children receiving trimethoprim/sulfamethoxazole presented a significantly lower risk of new antimicrobial resistances (RR: 0.12; 95% CI: 0.04–0.32) [66]. The retrospective study conducted by Lloyd et al. showed no significant superiority of nitrofurantoin or trimethoprim/sulfamethoxazole in terms of UTI recurrence [67] [Table 9].

In an updated Cochrane systematic review, it was observed that treatment with nitrofurantoin may lead to a lower risk of a UTI caused by a pathogen resistant to the treatment drug compared to treatment with trimethoprim–sulfamethoxazole (RR: 0.54, 95% CI: 0.31 to 0.92). However, patients receiving nitrofurantoin were twice as likely to experience side effects than patients receiving trimethoprim (RR: 2.18; 95% CI: 1.39–3.41). This suggests that the side effects of nitrofurantoin (number needed to harm = 3; 95% CI: 2–6) are similar to the possible benefits (NNT = 5; 95% CI: 3.0–33.0) compared with trimethoprim [6].

Italian guidelines suggested amoxicillin–clavulanic acid as a first-choice prophylactic agent, while ceftibuten or nitrofurantoin should be regarded as a secondary option, keeping in mind that nitrofurantoin may cause gastrointestinal intolerance and is inactive against most strains of *Proteus* spp. [32]. These recommendations are based on expert opinions due to the lack of data about the role of amoxicillin–clavulanic acid [Table S24].

### 3.10. Should the Prophylactic Antibiotic Be Changed after a Breakthrough UTI in Children already on Prophylaxis?

*Recommendation 10*

*There is insufficient evidence to recommend changing the prophylactic antibiotic after a breakthrough UTI in children already on prophylaxis (weak recommendation; quality of evidence: very low).*

A single retrospective study met the eligibility criteria for this clinical question. Shish et al. evaluated the risk of subsequent infections in children already receiving CAP for whom the drug was changed after a breakthrough UTI compared with children who continued the same antibiotic. The study presented a moderate methodological quality [Table 10]. The relative risk of a second BT-UTI when CAP was changed (versus unchanged) was 0.86 (*p* = 0.55), not statistically significant [68].

No recommendations from included guidelines were found for this clinical question.

### 3.11. Which Dosage Should Be Preferred for Continuous Antibiotic Prophylaxis?

*Recommendation 11*

*There is insufficient evidence to recommend a specific dose for continuous antibiotic prophylaxis.*

*Doses from one-quarter to one-third of the standard treatment dosage may be appropriate (weak recommendation; expert opinion).*

No clinical studies investigating the dosage of CAP in children met the eligibility criteria. In a Cochrane systematic review, no conclusions were reported about antibiotic dosages [6].

Guidelines from SINePe suggest one-quarter to one-third of the treatment dose, given once per day, as appropriate for CAP; however, there is no evidence to recommend a specific dose [32]. The KHA/CARI guidelines suggest CAP with co-trimoxazole at a dose of 2 mg of trimethoprim plus 10 mg of sulfamethoxazole per kilogram of body weight per day. This recommendation is based on the drugs used in PRIVENT and RIVUR trials, although data comparing different dosages are not available [34].

### 3.12. Should Antibiotic Prophylaxis Be Continued in Children Undergoing Pyeloplasty?

*Recommendation 12*

*In the absence of other persistent risk factors, antibiotic prophylaxis may be discontinued after pyeloplasty (weak recommendation against the intervention; quality of evidence: very low).*

Pooled results from three observational studies showed no significant benefits from continuing antibiotic prophylaxis after pyeloplasty in terms of recurrent UTIs (pooled RR: 1.22; 95% CI: 0.74–2.00) [69,70,71] [Table 11].

Two of the included studies enrolled more than 800 children undergoing pyeloplasty, and all of these children had a stent placed after surgery [69,70]. According to Vidovic et al., only female gender, diaper use, and positive intraoperative urine culture were significant risk factors for stent UTI [69]. An RCT aimed to analyze the effect of CAP in children with urinary stents after surgery, including pyeloplasty. Even though the sample size was limited, the trial showed a decreased rate of fUTI in patients with CAP, especially in those patients suffering from LUTS or previous UTI before surgery [70]. No systematic reviews or guidelines were included for this clinical question.

### 3.13. How Long Should Antibiotic Prophylaxis Be Continued in Children Undergoing Ablation of Posterior Urethral Valves?

*Recommendation 13*

*There is insufficient evidence to recommend how long antibiotic prophylaxis should be continued after ablation of posterior urethral valves.*

No clinical studies or systematic reviews were included for this clinical question. No recommendations from included guidelines concerned this clinical question. The CIRCUP study resulted in a lower rate of fUTI in the group of children with circumcision and CAP [38]. Based on these findings, EAU/ESPU guidelines suggest a strict follow-up in order to treat bladder dysfunction, which is the main risk factor for recurrent fUTI and long-term renal impairment [38].

### 3.14. How Long Should Antibiotic Prophylaxis Be Continued in Children Undergoing Ureteral Reimplantation?

*Recommendation 14*

*There is insufficient evidence to recommend how long antibiotic prophylaxis should be continued after ureteral reimplantation.*

No clinical studies or systematic reviews were included for this clinical question. No recommendations from included guidelines concerned this clinical question. However, CAP might be considered in patients with urinary stents after ureteral reimplantation.

### 3.15. How Long Should Antibiotic Prophylaxis Be Continued in Children Undergoing Endoscopic Treatment of VUR?

*Recommendation 15*

*There is insufficient evidence to recommend how long antibiotic prophylaxis should be continued in children undergoing endoscopic treatment of VUR. According to recommendations 3 and 4, antibiotic prophylaxis is not recommended in children with VUR of any grade.*

No clinical studies or systematic reviews were included for this clinical question. No recommendations from included guidelines concerned this clinical question.

## 4. Discussion

This systematic review shows the limited role of CAP in preventing UTI and its complications in children. To develop comprehensive recommendations, the efficacy and safety of CAP have been evaluated not just by the risk of UTI recurrence but also in terms of new renal scarring, new antimicrobial resistances, and drug-related adverse events.

Evidence-based benefits of CAP are limited to children with significant obstructive uropathies. Nonetheless, obstructive uropathies are extremely heterogeneous, and every different malformation presents a different risk of infection. Short-term prophylaxis may be considered also in children with a history of recurrent UTIs until the exclusion of urological anomalies.

As previously observed by Williams et al., CAP may reduce the recurrence of symptomatic infections in children with VUR, but the benefits are probably small and must be weighed against the likelihood of antimicrobial resistances and the lack of effect on the risk of renal scarring [6]. These findings seem to also be confirmed in children with high-grade (III–V) or dilating VUR. The risk of renal scarring and the possible loss of renal function led to the common use of CAP in children with VUR. To prevent the long-term complications of renal scarring, some guidelines supported the role of CAP, particularly in children with high-grade VUR. However, available evidence shows that CAP is not effective in preventing new renal scars, as reported by RiVUR and Swedish reflux trials [42,52]. The results of the RiVUR trial were further analyzed to examine the relationship between VUR and renal scarring. In a post hoc analysis by Shaikh et al., the risk of renal scarring increased substantially with a second febrile UTI [72].

Some authors suggested that reducing the risk of UTI recurrence may affect the risk of renal scarring, although there is still no direct evidence [73]. Bandari et al. showed that the high grades of antenatal hydroureteronephrosis, febrile UTI, younger age, and *E. coli* UTI were more associated with recurrent attacks of UTI and renal scarring [74]. Our results show that CAP significantly increases the risk of breakthrough infections caused by resistant uropathogens leading to the ineffectiveness of empiric antibiotics and increased risk of acute and chronic complications, including renal scarring. Weighing the limited benefits against the proved risks, we suggest limiting the use of CAP in children with VUR. Close surveillance aimed at early diagnosis of UTIs and prompt antibiotic treatment seems to be a reasonable approach for reducing the risk of renal scarring and avoiding the misuse of antibiotics and consequent risks of antimicrobial resistances.

Finally, since several factors account for the occurrence of UTI in patients affected by VUR, EAU/ESPU guidelines suggested a risk assessment in order to guide the caregivers in the clinical management and to avoid overtreatment consisting of unnecessarily prolonged administration of antibiotics and surgical procedures. A greater benefit resulted from the use of CAP in children with significant obstructive uropathies, particularly primary obstructive megaureter. In these children, short-term prophylaxis is suggested by several guidelines until surgical correction is performed. Nevertheless, it is relevant to emphasize there is a lack of evidence about the prolongation of CAP after ureteral reimplantation and endoscopic procedures and for patients with urinary stents after surgery. These aspects are considered a hot topic by most pediatric urologists and scientific societies. A systematic review dealing with the need for CAP after pediatric stented pyeloplasty has been registered on PROSPERO (https://www.crd.york.ac.uk/prospero/display_record.php?ID=CRD42022371212, accessed on 30 May 2023).

Since the current evidence might limit the impact of CAP, non-pharmacological strategies should be taken into account for the prevention of UTI recurrency in children affected by congenital uropathies and VUR. First, BBD worsens the risk of UTI in this category of children. A correct micturition pattern to restore bladder function together with regular bowel function should be a primary goal for clinicians and caregivers. This aspect is crucial. BBD might play a significant role in the pathogenesis of recurrent UTIs and could undermine the outcomes of the operative treatment. Second, even though prophylactic circumcision might raise controversies and ethical concerns, it is relevant to highlight that circumcision significantly decreased the risk of UTI in children affected by posterior urethral valves.

The effects of CAP in children with neurogenic bladder are controversial. Available studies mainly concern patients with spina bifida undergoing clean intermittent catheterization. In this population, the correct management of catheterization seems more effective than CAP in preventing UTIs. Moreover, the risk of infections caused by resistant uropathogens is substantial for these patients and may further limit the effectiveness of available antibiotics.

There is insufficient evidence to recommend specific drugs and dosages as first-choice prophylactic regimens. Available studies compared only trimethoprim–sulfamethoxazole, nitrofurantoin, and oral cephalosporins, showing an increased risk of antimicrobial resistances using the latter without a clear superiority between the other drugs. Amoxicillin–clavulanic acid has been suggested only by the Italian guidelines, without strong supporting evidence. This recommendation was probably based on the effectiveness of this drug as an empiric antibiotic to treat UTIs; however, its use may increase the emergence of resistant uropathogens [75].

The main limitations of this systematic review are the lack of a meta-analysis and the unavailability of evidence for some outcomes of the proposed clinical questions. Therefore, some recommendations are still based on expert opinions. Moreover, patient values and preferences were assessed only by a few of the included studies. Most of the included clinical trials presented a low risk of bias; however, certainty of results was often downgraded due to imprecision. The main reason was the limited size of study populations, particularly when considering outcomes different from UTI recurrence. In addition, this article is focused on high-income countries [7,8,9]. Few studies have been conducted in low-income countries, and it is possible that the findings could be different in some of these countries.

## 5. Conclusions

Our systematic review shows that CAP plays a limited role in preventing recurrences of UTI in children and has no effect on its complications. On the other hand, the emergence of new antimicrobial resistances is a proven risk, and antimicrobial stewardship is one of the measures that has shown the highest efficacy in reducing antibiotic abuse and misuse in order to fight this public health emergency [76]. The benefits from CAP seem to overcome the risks only in children with significant obstructive uropathies, and only until the surgical correction. Moreover, correct bladder and bowel training should be emphasized in children affected by VUR and other congenital uropathies. Our study provides evidence-based recommendations to guide clinicians on the correct use of CAP for the prevention of UTI and its complications in children (Table 12). The misuse of antibiotics is a leading cause of the spread of resistant uropathogens in community-acquired pediatric UTIs; thus, prescriptions should be limited to contexts of proven efficacy and safety.

## Figures and Tables

**Table 2:** Pooled results and certainty assessment of randomized clinical trials and observational studies involving children with recurrent UTIs (second clinical question).

Certainty Assessment							No. of Patients		Effect		Certainty
No. of Studies	Study Design	Risk of Bias	Inconsistency	Indirectness	Imprecision	Other Considerations	Antibiotic Prophylaxis	No Prophylaxis	Relative (95% CI)	Absolute (95% CI)	
**Risk of UTI recurrence (follow-up: mean 12 months; assessed as rates of recurrence)**											
1 [20]	randomized trials	not serious ^a^	not serious	not serious	very serious ^b^	none	15/54 (27.8%)	16/44 (36.4%)	**RR 0.76** (0.43 to 1.37)	**87 fewer per 1000** (207 fewer–135 more)	⨁⨁◯◯ Low
**Risk of new antimicrobial resistances (assessed as rates of infections resistant to empiric antibiotics)**											
1 [35]	observational studies	not serious ^c^	not serious	not serious	very serious ^d^	none	4/10 (40.0%)	7/27 (25.9%)	**RR 1.54** (0.57 to 4.16)	**140 more per 1000** (111 fewer–819 more)	⨁◯◯◯ Very low

CI: confidence interval; HR: hazard ratio; OR: odds ratio; RR: risk ratio. ^a^: complete risk of bias analysis with results for single domains of the RoB2 tool is presented in Supplementary Materials. ^b^: OIS criterion is not met and 95% CI overlaps no effect. ^c^: complete risk of bias analysis with results for Newcastle–Ottawa Scale tool is presented in Supplementary Tables and Figures. ^d^: OIS criterion is not met.

**Table 3:** Pooled results and certainty assessment of randomized clinical trials and observational studies involving children with VUR of any grade (third clinical question).

Certainty Assessment							No. of Patients		Effect		Certainty
No. of Studies	Study Design	Risk of Bias	Inconsistency	Indirectness	Imprecision	Other Considerations	Antibiotic Prophylaxis	No Prophylaxis	Relative (95% CI)	Absolute (95% CI)	
**Risk of UTI recurrence (follow-up: mean 18 months; assessed as rates of recurrence)**											
5 [20,22,40,41,42]	randomized trials	not serious ^a^	not serious	not serious	not serious	none	140/632 (22.2%)	195/656 (29.7%)	**RR 0.75** (0.62 to 0.90)	**74 fewer per 1000** (113 fewer–30 fewer)	⨁⨁⨁⨁ High
**Risk of UTI recurrence (follow-up: mean 44 months; assessed as rates of recurrence)**											
2 [43,44]	observational studies	not serious ^b^	not serious	not serious	serious ^c^	none	80/449 (17.8%)	53/368 (14.4%)	**RR 1.11** (0.96 to 1.30)	**16 more per 1000** (6 fewer–43 more)	⨁◯◯◯ Very low
**Risk of new renal scars (follow-up: mean 20 months; assessed as rates of new renal scars on DMSA scan)**											
3 [22,41,42]	randomized trials	not serious	not serious	not serious	serious ^c^	none	23/325 (7.1%)	21/335 (6.3%)	**RR 1.13** (0.64 to 2.00)	**8 more per 1000** (23 fewer–63 more)	⨁⨁⨁◯ Moderate
**Risk of new antimicrobial resistances (follow-up: mean 24 months; assessed as rates of infections resistant to empiric antibiotics)**											
1 [41]	randomized trials	not serious	not serious	not serious	serious ^d^	strong association	26/38 (68.4%)	17/69 (24.6%)	**RR 2.78** (1.74 to 4.42)	**439 more per 1000** (182 more–843 more)	⨁⨁⨁⨁ High
**Risk of drug-related adverse events (follow-up: mean 24 months; assessed as rates of drug-related adverse event)**											
1 [41]	randomized trials	not serious	not serious	not serious	serious ^e^	none	153/302 (50.7%)	165/305 (54.1%)	**RR 0.94** (0.80 to 1.09)	**32 fewer per 1000** (108 fewer–49 more)	⨁⨁⨁◯ Moderate

CI: confidence interval; RR: risk ratio. ^a^: complete risk of bias analysis with results for single domains of the RoB2 tool is presented in Supplementary Tables and Figures. ^b^: complete risk of bias analysis with results for Newcastle–Ottawa Scale tool is presented in Supplementary Tables and Figures. ^c^: OIS criterion is not met and 95% CI overlaps no effect. ^d^: OIS criterion is not met. ^e^: 95% CI overlaps no effect.

**Table 4:** Pooled results and certainty assessment of randomized clinical trials and observational studies involving children with high-grade VUR (fourth clinical question).

Certainty Assessment							No. of Patients		Effect		Certainty
No. of Studies	Study Design	Risk of Bias	Inconsistency	Indirectness	Imprecision	Other Considerations	Antibiotic Prophylaxis	No Prophylaxis	Relative (95% CI)	Absolute (95% CI)	
**Risk of UTI recurrence (follow-up: mean 18 months; assessed as rates of recurrence)**											
2 [20,50]	randomized trials	not serious ^a^	not serious	not serious	serious ^b^	strong association	19/134 (14.2%)	38/132 (28.8%)	**RR 0.49** (0.30 to 0.81)	**147 fewer per 1000** (202 fewer–55 fewer)	⨁⨁⨁⨁ High
**Risk of new renal scars (follow-up: mean 24 months; assessed as rates of new renal scars on DMSA scan)**											
1 [51]	randomized trials	not serious	not serious	not serious	very serious ^c^	none	4/68 (5.9%)	12/68 (17.6%)	**RR 0.47** (0.20 to 1.11)	**94 fewer per 1000** (141 fewer–19 more)	⨁⨁◯◯ Low

CI: confidence interval; RR: risk ratio. ^a^: complete risk of bias analysis with results for single domains of the RoB2 tool is presented in Supplementary Materials. ^b^: OIS criterion is not met. ^c^: OIS criterion is not met and 95% CI overlaps no effect.

**Table 5:** Pooled results and certainty assessment of randomized clinical trials and observational studies involving children with isolated hydronephrosis (fifth clinical question).

Certainty Assessment							No. of Patients		Effect		Certainty
No. of Studies	Study Design	Risk of Bias	Inconsistency	Indirectness	Imprecision	Other Considerations	Antibiotic Prophylaxis	No Prophylaxis	Relative (95% CI)	Absolute (95% CI)	
**Risk of UTI (follow-up: mean 12.5 months; assessed as rates of recurrence)**											
2 [53,54]	observational studies	not serious ^a^	serious ^b^	not serious	serious ^c^	none	69/435 (15.9%)	72/464 (15.5%)	**RR 1.01** (0.84 to 1.22)	**2 more per 1000** (25 fewer–34 more)	⨁◯◯◯ Very low

CI: confidence interval; RR: risk ratio. ^a^: complete risk of bias analysis with results for Newcastle–Ottawa Scale tool is presented in Supplementary Materials. ^b^: event rates are significantly different between studies. ^c^: OIS criterion is not met and 95% CI overlaps no effect.

**Table 6:** Pooled results and certainty assessment of randomized clinical trials and observational studies involving children with hydroureteronephrosis (seventh clinical question).

Certainty Assessment							No. of Patients		Effect		Certainty
No. of Studies	Study Design	Risk of Bias	Inconsistency	Indirectness	Imprecision	Other Considerations	Antibiotic Prophylaxis	No Prophylaxis	Relative (95% CI)	Absolute (95% CI)	
**Risk of UTI (follow-up: mean 33 months; assessed as rates of recurrence)**											
3 [56,57,58]	observational studies	Serious ^a^	not serious	not serious	not serious	strong association	44/219 (20.1%)	47/115 (40.9%)	**RR 0.49** (0.35 to 0.69)	**208 fewer per 1000** (266 fewer–127 fewer)	⨁⨁◯◯ Low

CI: confidence interval; RR: risk ratio. ^a^: complete risk of bias analysis with results for Newcastle–Ottawa Scale tool is presented in Supplementary Tables and Figures.

**Table 7:** Pooled results and certainty assessment of randomized clinical trials and observational studies involving children with neurogenic bladder (eighth clinical question).

Certainty Assessment							No. of Patients		Effect		Certainty
No. of Studies	Study Design	Risk of Bias	Inconsistency	Indirectness	Imprecision	Other Considerations	Antibiotic Prophylaxis	No Prophylaxis	Relative (95% CI)	Absolute (95% CI)	
**Risk of UTI recurrence (follow-up: mean 11 months; assessed as rates of recurrence)**											
2 [60,61]	randomized trials	serious ^a^	serious ^b^	not serious	serious ^c^	strong association	22/119 (18.5%)	7/110 (6.4%)	**RR 2.91** (1.29 to 6.53)	**122 more per 1000** (18 more–352 more)	⨁⨁◯◯ Low
**Risk of UTI recurrence (follow-up: mean 24 months; assessed as rates of recurrence)**											
1 [62,63]	observational studies	not serious ^d^	not serious	not serious	not serious	none	43/85 (50.6%)	23/36 (63.9%)	**RR 0.79** (0.57 to 1.09)	**134 fewer per 1000** (275 fewer–58 more)	⨁⨁◯◯ Low
**Risk of new antimicrobial resistances (follow-up: mean 18 months; assessed as rates of infections resistant to empiric antibiotics)**											
1 [64]	randomized trials	not serious ^a^	not serious	Serious ^e^	not serious	none	248/343 (72.3%)	197/370 (53.2%)	**RR 1.57** (1.31 to 1.89)	**303 more per 1000** (165 more–474 more)	⨁⨁⨁◯ Moderate

CI: confidence interval; RR: risk ratio. ^a^: complete risk of bias analysis with results for single domains of the RoB2 tool is presented in Supplementary Tables and Figures. ^b^: event rates are significantly different between studies. ^c^: OIS criterion is not met. ^d^: complete risk of bias analysis with results for Newcastle–Ottawa Scale tool is presented in Supplementary Tables and Figures. ^e^: outcome measured as rate of resistant isolates in positive urine samples from each group, not as prevalence of resistant UTIs in patients.

**Table 8:** Pooled results and certainty assessment of randomized clinical trials and observational studies involving children receiving different prophylactic antibiotics (ninth clinical question).

Certainty Assessment							No. of Patients		Effect		Certainty
No. of Studies	Study Design	Risk of Bias	Inconsistency	Indirectness	Imprecision	Other Considerations	Antibiotic Prophylaxis with Co-Trimoxazole	Oral Cephalosporins	Relative (95% CI)	Absolute (95% CI)	
**Risk of UTI recurrence (follow-up: mean 12 months; assessed as rates of recurrence)**											
1 [65]	randomized trials	not serious ^a^	not serious	not serious	Serious ^b^	none	10/75 (13.3%)	8/78 (10.3%)	**RR 1.30** (0.54 to 3.12)	**31 more per 1000** (47 fewer–217 more)	⨁⨁⨁◯ Moderate
**Risk of UTI recurrence (follow-up: mean 25 months; assessed as rates of recurrence)**											
1 [66]	observational studies	not serious ^c^	not serious	not serious	very serious ^b^	none	66/205 (32.2%)	36/144 (25.0%)	**RR 1.29** (0.91 to 1.82)	**73 more per 1000** (22 fewer–205 more)	⨁◯◯◯ Very low
**Risk of new antimicrobial resistances (follow-up: mean 25 months; assessed as rates of infections resistant to empiric antibiotics)**											
1 [66]	observational studies	not serious ^c^	not serious	not serious	very serious ^b^	very strong association	4/66 (6.1%)	17/33 (51.5%)	**RR 0.12** (0.04 to 0.32)	**453 fewer per 1000** (495 fewer–350 fewer)	⨁⨁◯◯ Low

CI: confidence interval; HR: hazard ratio; OR: odds ratio; RR: risk ratio. ^a^: complete risk of bias analysis with results for single domains of the RoB2 tool is presented in Supplementary Materials. ^b^: OIS criterion is not met and 95% CI overlaps no effect. ^c^: complete risk of bias analysis with results for Newcastle–Ottawa Scale tool is presented in Supplementary Tables and Figures.

**Table 9:** Pooled results and certainty assessment of randomized clinical trials and observational studies involving children receiving different prophylactic antibiotics (ninth clinical question).

Certainty Assessment							No. of Patients		Effect		Certainty
No. of Studies	Study Design	Risk of Bias	Inconsistency	Indirectness	Imprecision	Other Considerations	Antibiotic Prophylaxis with Co-Trimoxazole	Nitrofurantoine	Relative (95% CI)	Absolute (95% CI)	
**Risk of UTI recurrence (assessed as rates of recurrence)**											
1 [67]	observational studies	not serious ^a^	serious ^b^	not serious	serious ^c^	none	10/170 (5.9%)	1/13 (7.7%)	**RR 0.76** (0.11 to 5.52)	**18 fewer per 1000** (68 fewer–348 more)	⨁◯◯◯ Very low

CI: confidence interval; RR: risk ratio. ^a^: complete risk of bias analysis with results for Newcastle–Ottawa Scale tool is presented in Supplementary Tables and Figures. ^b^: sizes of groups are significantly different. ^c^: OIS criterion is not met and 95% CI overlaps no effect.

**Table 10:** Pooled results and certainty assessment of randomized clinical trials and observational studies involving children who continued or changed the antibiotic after a breakthrough infection (tenth clinical question).

Certainty Assessment							No. of Patients		Effect		Certainty
No. of Studies	Study Design	Risk of Bias	Inconsistency	Indirectness	Imprecision	Other Considerations	A Different Antibiotic	The Same Antibiotic	Relative (95% CI)	Absolute (95% CI)	
**Risk of new UTI (follow-up: mean 24 months; assessed with rate of new UTI with positive urine culture)**											
1 [68]	observational studies	not serious ^a^	serious ^b^	not serious	not serious	none	12/24 (50.0%)	22/38 (57.9%)	**RR 0.82** (0.44 to 1.54)	**104 fewer per 1000** (324 fewer–313 more)	⨁◯◯◯ Very low

CI: confidence interval; HR: hazard ratio; OR: odds ratio; RR: risk ratio. ^a^: complete risk of bias analysis with results for Newcastle–Ottawa Scale tool is presented in Supplementary Tables and Figures. ^b^: OIS criterion is not met and 95% CI overlaps no effect.

**Table 11:** Pooled results and certainty assessment of randomized clinical trials and observational studies involving children who underwent pyeloplasty.

Certainty Assessment							No. of Patients		Effect		Certainty
No. of Studies	Study Design	Risk of Bias	Inconsistency	Indirectness	Imprecision	Other Considerations	Antibiotic Prophylaxis	No Prophylaxis	Relative (95% CI)	Absolute (95% CI)	
**Risk of UTI recurrence (follow-up: mean 2 months; assessed with rate of recurrences with positive urine culture)**											
3 [69,70,71]	observational studies	not serious ^a^	not serious	not serious	serious ^b^	none	36/520 (6.9%)	25/441 (5.7%)	**RR 1.22** (0.74 to 2.00)	**12 more per 1000** (15 fewer–57 more)	⨁◯◯◯ Very low

CI: confidence interval; HR: hazard ratio; OR: odds ratio; RR: risk ratio. ^a^: complete risk of bias analysis with results for Newcastle–Ottawa Scale tool is presented in Supplementary Tables and Figures. ^b^: OIS criterion is not met and 95% CI overlaps no effect.

**Table 12:** Summary of recommendations with strength of recommendations and quality of evidence. According to the Grading of Recommendations, Assessment, Development, and Evaluations (GRADE) methodology.

Clinical Questions	Recommendations	Strength and Quality
Should continuous antibiotic prophylaxis be used in all children with a previous UTI?	Continuous antibiotic prophylaxis is not routinely indicated in all children after the first episode of UTI.	Strong recommendation against the intervention. Evidence quality: B
Should continuous antibiotic prophylaxis be used in all children with a history of recurrent UTIs?	A history of recurrent UTIs without underlying urological anomalies does not constitute a sufficient indication for continuous antibiotic prophylaxis.	Weak recommendation against the intervention. Evidence quality: C
	Short-term prophylaxis may be considered until the exclusion of urological anomalies.	Weak recommendation for the intervention. Expert opinion
Should continuous antibiotic prophylaxis be used in all children with VUR of any grade?	Continuous antibiotic prophylaxis is not recommended for children with low-grade (I–II) or non-dilating VUR.	Strong recommendation against the intervention. Evidence quality: B
	Close surveillance based on early diagnosis (i.e., urinalysis and urine culture) and prompt antibiotic therapy in symptomatic/febrile children may be considered in children with VUR of any grade.	Weak recommendation. Expert opinion
Should continuous antibiotic prophylaxis be used in all children with high-grade VUR (III–V)?	Considering the lack of effect of antibiotic prophylaxis on the risk of renal scarring, continuous antibiotic prophylaxis is not routinely recommended in children with high-grade (III–IV) or dilating VUR.	Weak recommendation against the intervention. Evidence quality: B
	Close surveillance based on early diagnosis (i.e., urinalysis and urine culture) and prompt antibiotic therapy in symptomatic/febrile children is recommended in children with VUR of any grade.	Weak recommendation. Expert opinion
Should antibiotic prophylaxis be used in children with isolated hydronephrosis?	Continuous antibiotic prophylaxis is not routinely recommended in children with isolated antenatal or postnatal hydronephrosis or ureteropelvic junction obstruction.	Weak recommendation against the intervention. Evidence quality: C
Should antibiotic prophylaxis be used in children with infravesical obstructions (i.e., urethral valves)?	There is no sufficient evidence to define the efficacy and safety of continuous antibiotic prophylaxis in children with infravesical obstructions. Continuous antibiotic prophylaxis may be considered until surgical correction.	Weak recommendation for the intervention. Expert opinion
Should antibiotic prophylaxis be used in children with hydroureteronephrosis (i.e., primary obstructive megaureter)?	Continuous antibiotic prophylaxis may be considered in children with hydroureteronephrosis and ureteral dilation > 7 mm or primary obstructive megaureter.	Weak recommendation for the intervention. Evidence quality: C
Should antibiotic prophylaxis be used in children with neurogenic bladder?	Continuous antibiotic prophylaxis is not routinely recommended in children affected by neurogenic bladder.	Weak recommendation against the intervention. Evidence quality: C
	Proper execution of clean intermittent catheterization and close surveillance, based on early diagnosis (i.e., urinalysis and urine culture) and prompt antibiotic therapy in symptomatic/febrile children, may be considered in children with neurogenic bladder.	Weak recommendation. Expert opinion
Which antibiotic should be preferred for long-term prophylaxis of UTI in children?	There is insufficient evidence to recommend trimethoprim–sulfamethoxazole rather than nitrofurantoin as the first-choice prophylactic antibiotic. There is no evidence on the efficacy and safety of amoxicillin–clavulanic acid as a prophylactic antibiotic to prevent UTIs. The prophylactic use of oral cephalosporins is not suggested due to the high risk of new antimicrobial resistances	Weak recommendation against the intervention. Evidence quality: C
Should the prophylactic antibiotic be changed after a breakthrough UTI in children already on prophylaxis?	There is insufficient evidence to recommend changing the prophylactic antibiotic after a breakthrough UTI in children already on prophylaxis.	Weak recommendation. Evidence quality: D
Which dosage should be preferred for continuous antibiotic prophylaxis?	There is insufficient evidence to recommend a specific dose for continuous antibiotic prophylaxis. Doses from one-quarter to one-third of the standard treatment dosage may be appropriate	Weak recommendation. Expert opinion
Should antibiotic prophylaxis be continued in children undergoing pyeloplasty?	In the absence of other persistent risk factors, antibiotic prophylaxis may be discontinued after pyeloplasty.	Weak recommendation against the intervention. Evidence quality: C
How long should antibiotic prophylaxis be continued in children undergoing ablation of posterior urethral valves?	There is insufficient evidence to recommend how long antibiotic prophylaxis should be continued after ablation of posterior urethral valves.	
How long should antibiotic prophylaxis be continued in children undergoing ureteral reimplantation?	There is insufficient evidence to recommend how long antibiotic prophylaxis should be continued after ureteral reimplantation.	
How long should antibiotic prophylaxis be continued in children undergoing endoscopic treatment of VUR?	There is insufficient evidence to recommend how long antibiotic prophylaxis should be continued in children undergoing endoscopic treatment of VUR. According to recommendations 3 and 4, antibiotic prophylaxis is not routinely recommended in children with VUR of any grade.	

## Data Availability

All the data are included in the manuscript.

## Supplementary Materials

The following supporting information can be downloaded at: https://www.mdpi.com/article/10.3390/antibiotics12061040/s1, Supplementary Material S1: Clinical questions and PICO items.
