# Supplementary material for: Antibiotic Prophylaxis for the Prevention of Urinary Tract Infections in Children: Guideline and Recommendations from the Emilia-Romagna Pediatric Urinary Tract Infections (UTI-Ped-ER) Study Group
Source: Antibiotics (Basel). 2023 Jun 12;12(6):1040. doi: 10.3390/antibiotics12061040 (PMC10295299; doi:10.3390/antibiotics12061040)
Supplement: Supplementary file 1 [file antibiotics-12-01040-s001.zip › antibiotics-2430639-supplementary.pdf]

Supplementary Material S1

Clinical questions and PICO items

Should antibiotic prophylaxis be used in all children with a previous UTI?
P: children aged <18 years with a single previous UTI
I: long-term antibiotic prophylaxis with any molecule
C: no prophylaxis
O1: risk of UTI recurrence
O2: risk of new renal scars
O3: risk of new antimicrobial resistances
O4: risk of drug-related adverse events

Should antibiotic prophylaxis be used in all children with recurrent UTIs?
P: children aged <18 years with recurrent UTIs
I: long-term antibiotic prophylaxis with any molecule
C: no prophylaxis
O1: risk of UTI recurrence
O2: risk of new renal scars
O3: risk of new antimicrobial resistances
O4: risk of drug-related adverse events

Should antibiotic prophylaxis be used in children with VUR (any grade)?
P: children aged <18 years with VUR (any grade)
I: long-term antibiotic prophylaxis with any molecule
C: no prophylaxis
O1: risk of UTI recurrence
O2: risk of new renal scars
O3: risk of new antimicrobial resistances
O4: risk of drug-related adverse events

Should antibiotic prophylaxis be used in children with high-grade VUR (III-V)?
P: children aged <18 years with VUR (grade III-V)
I: long-term antibiotic prophylaxis with any molecule
C: no prophylaxis
O1: risk of UTI recurrence
O2: risk of new renal scars
O3: risk of new antimicrobial resistances
O4: risk of drug-related adverse events

Should antibiotic prophylaxis be used in children with isolated hydronephrosis?
P: children aged <18 years with isolated hydronephrosis
I: long-term antibiotic prophylaxis with any molecule
C: no prophylaxis
O1: risk of UTI recurrence
O2: risk of new renal scars

O3: risk of new antimicrobial resistances
O4: risk of drug-related adverse events

Should antibiotic prophylaxis be used in children with infravesical obstructions (urethral valves)?
P: children aged <18 years with infravesical obstruction (urethral valves)
I: long-term antibiotic prophylaxis with any molecule
C: no prophylaxis
O1: risk of UTI recurrence
O2: risk of new renal scars
O3: risk of new antimicrobial resistances
O4: risk of drug-related adverse events

Should antibiotic prophylaxis be used in children with hydroureteronephrosis/ureteral dilation (primary obstructive megaureter)?
P: children aged <18 years with hydroureteronephrosis/ureteral dilation (primary obstructive megaureter)
I: long-term antibiotic prophylaxis with any molecule
C: no prophylaxis
O1: risk of UTI recurrence
O2: risk of new renal scars
O3: risk of new antimicrobial resistances
O4: risk of drug-related adverse events

Should antibiotic prophylaxis be used in children with neurogenic bladder?
P: children aged <18 years with neurogenic bladder
I: long-term antibiotic prophylaxis with any molecule
C: no prophylaxis
O1: risk of UTI recurrence
O2: risk of new renal scars
O3: risk of new antimicrobial resistances
O4: risk of drug-related adverse events

Which antibiotic should be preferred for long-term prophylaxis of UTI in children?
P: children aged <18 years at risk of UTI
I1: oral cephalosporins
I2: trimethoprim-sulfamethoxazole
I3: nitrofurantoin
C: amoxicillin-clavulanic acid
O1: risk of UTI recurrence
O2: risk of new renal scars
O3: risk of new antimicrobial resistances
O4: risk of drug-related adverse events

After a breakthrough UTI in children already on prophylaxis, which antibiotic should be preferred to continue the prophylaxis?
P: children aged <18 years on antibiotic prophylaxis who experience a breakthrough UTI
I: to continue with the same antibiotic

C: to change with a different antibiotic
O1: risk of UTI recurrence
O2: risk of new renal scars
O3: risk of new antimicrobial resistances
O4: risk of drug-related adverse events

Which dosage should be preferred for long-term antibiotic prophylaxis?
P: children aged <18 years at risk of UTI
I: ⅓ of the standard dosage
C: ½ of the standard dosage
O1: risk of UTI recurrence
O2: risk of new renal scars
O3: risk of new antimicrobial resistances
O4: risk of drug-related adverse events

How long should antibiotic prophylaxis be continued in children undergoing pyeloplasty?
P: children aged <18 years who underwent pyeloplasty
I: to continue prophylaxis after pyeloplasty
C: to discontinue prophylaxis after pyeloplasty
O1: risk of UTI recurrence
O2: risk of new renal scars
O3: risk of new antimicrobial resistances
O4: risk of drug-related adverse events
How long should antibiotic prophylaxis be continued in children undergoing ablation of posterior urethral valves?
P: children aged <18 years who underwent surgery for posterior urethral valves
I: to continue prophylaxis after surgery
C: to discontinue prophylaxis after surgery
O1: risk of UTI recurrence
O2: risk of new renal scars
O3: risk of new antimicrobial resistances
O4: risk of drug-related adverse events

How long should antibiotic prophylaxis be continued in children undergoing ureteral reimplantation?
P: children aged <18 years who underwent ureteral reimplantation
I: to continue prophylaxis after surgery
C: to discontinue prophylaxis after surgery
O1: risk of UTI recurrence
O2: risk of new renal scars
O3: risk of new antimicrobial resistances
O4: risk of drug-related adverse events

How long should antibiotic prophylaxis be continued in children undergoing endoscopic treatment of VUR?
P: children aged <18 years who underwent endoscopic treatment of VUR
I: to continue prophylaxis after endoscopy

C: to discontinue prophylaxis after endoscopy
O1: risk of UTI recurrence
O2: risk of new renal scars
O3: risk of new antimicrobial resistances
O4: risk of drug-related adverse events

SEARCH QUERIES

(newborn* OR neonat* OR infan* OR toddler* OR pre-schooler* OR preschooler* OR child* OR children OR adolescen* OR pediatr* OR paediatr* OR youth* OR teen* OR kid OR baby OR babies)
AND
(
(UTI OR urinary tract infection* OR pyelonephritis OR urinary infection* OR renal abscess OR kidney abscess OR kidney infection* OR nephritis OR pyelitis)
OR
(vesicoureteral reflux OR VUR OR urine reflux OR renal reflux OR urinary reflux OR ureteral reflux)
OR
(hydronephrosis OR kidney swelling OR renal swelling) OR (urethral obstruction* OR urethral valve* OR PUV OR obstructive uropat*)
OR
(ureteral obstruction* OR megaureter OR ureteropelvic junction stenosis OR pyeloureteral junction stenosis OR ureteropelvic junction obstruction* OR pyeloureteral junction obstruction* OR ureteral swelling OR pyeloureteral swelling OR ureteral stenosis OR ureteral obstruction* OR vesicoureteral stenosis OR vesicoureteral obstruction* OR UPJO)
OR
(neurogenic bladder OR neurogenic urinary tract dysfunction* OR neurological bladder OR neurological urinary tract disorder* OR neuro* incontinence OR hyperactive bladder OR detrusor overactivity)
OR
(urinary surger* OR urinary tract surger* OR urolog* surger* OR urolog* endoscop* OR cystoscop* OR ureteroscop* OR ureteral reimplant* OR pyeloplast* OR urethral valve* ablation* OR urethral valve* resection* OR urethral valve* fulguration OR vesicoureteral reflux treatment* OR vesicoureteral reflux correction* OR vesicoureteral reflux surger* OR endoscopic injection therap* OR ureteral stent* OR urinary diversion* OR deflux)
)
AND
(antibiotic prophylaxis OR prophylaxis OR antibiotic premedication* OR antimicrobial prophylaxis OR antimicrobial premedication* OR premedication* OR prevention OR secondary prevention OR antimicrobial prevention OR antimicrobial secondary prevention OR antibiotic prevention OR antibiotic secondary prevention)

AND
(
(posolog* OR dose* OR dosage*)
OR
(duration OR duration administration OR time administration OR length administration OR days therapy OR days treatment OR DOT OR length therapy OR length treatment OR LOT OR duration prophylaxis OR length prophylaxis OR time prophylaxis OR duration prevention OR length prevention OR time prevention OR length OR time)
OR
(first-line OR first line OR first-line therap* OR first line therap* OR first line management* OR first-line management OR first line treatment* OR first-line treatment* OR first-choice therap* OR first choice therap* OR first choice management* OR first-choice management OR first choice treatment OR first-choice treatment)
OR
(second-line OR second line OR second-line therap* OR second line therap* OR second line management* OR second-line management OR second line treatment* OR second-line treatment* OR second-choice therap* OR second choice therap* OR second choice management* OR second-choice management OR second choice treatment OR second-choice treatment)
OR
(relapse* OR recurrence* OR recurrent OR recurrent infection* OR recrudescence* OR recurrent UTI)
OR
(scar* OR renal scar* OR kidney scar* OR kidney injur* OR renal injur* OR parenchymal defect* OR renal damage* OR kidney damage* OR parenchymal scar* OR cicatrix)
OR
(drug resistan* OR antimicrobial resistan* OR antibacterial resistan* OR antibiotic resistan*)
OR
(drug-related side effect* OR drug-related adverse reaction* OR drug-related toxicity OR adverse effect* OR adverse drug reaction* OR drug side effect* OR adverse drug event*)
)

EXCLUDED ARTICLES

Table S1. Characteristics of excluded articles and reasons for exclusion.

TITLE	DESIGN	REASON FOR EXCLUSION
Interventions for primary vesicoureteric reflux (Wheeler et al, 2004)	Systematic review	Previous version of the same systematic review already included in our review.

Interventions for primary vesicoureteric reflux (Hodson et al, 2007).	Systematic review	Previous version of the same systematic review already included in our review.
Interventions for primary vesicoureteric reflux (Nagler et al, 2010).	Systematic review	Previous version of the same systematic review already included in our review.
Long-term antibiotics for preventing recurrent urinary tract infection in children (Williams et al, 2011)	Systematic review	Previous version of the same systematic review already included in our review.
Urinary tract infections in children: EAU/ESPU guidelines (Stein et al, 2015)	Guideline	Previous version of the guideline by EAU/ESPU already included in our review.
Outcome at 10 years of severe vesicoureteric reflux managed medically: Report of the International Reflux Study in Children (Smellie et al, 2001)	Randomized clinical trial	The trial investigated the rates of VUR resolution in children treated with antibiotic prophylaxis or endoscopic surgery. This outcome was not included in our review.
Outcomes of Targeted Treatment for Vesicoureteral Reflux in Children with Nonneurogenic Lower Urinary Tract Dysfunction (Fast et al,2013)	Observational study	The study evaluated a cohort of children with VUR associated with non-neurogenic lower urinary tract dysfunctions. No control group was included. No comparison was made with alternative interventions.
The outcome of stopping prophylactic antibiotics in older children with vesicoureteral reflux (Cooper et al, 2000)	Observational study	The study evaluated the rate of UTI recurrence in a group of children with VUR who discontinued prophylaxis. No control group was included. No comparison was made with alternative interventions.
The Swedish infant high-grade reflux trial: Study presentation and vesicoureteral reflux outcome (Nordenström et al, 2016)	Clinical Trial	The trial investigated the rates of VUR resolution in children treated with antibiotic prophylaxis or endoscopic surgery. This outcome was not included in our review.
Antibiotic prophylaxis by low-dose cefaclor in children with vesicoureteral reflux (Kaneko et al, 2003)	Observational study	The study evaluated the rate of UTI recurrence in a group of children with VUR treated with Cefaclor. No control group was included. No comparison was made with alternative interventions.
Summary of the AUA Guideline on Management of Primary Vesicoureteral Reflux in Children (Peters et al, 2010)	Commentary	The study is a summary of the AUA guideline on management of primary vesicoureteral reflux in children. The original guideline was already included in our review.
Ten-year results of randomized treatment of children with severe vesicoureteral reflux. Final report of the International Reflux Study in Children (Jodal et al, 2006)	Randomized clinical trial	The trial compared the medical and surgical treatment of VUR in terms of reflux resolution and renal scarring. Our review is not aimed to compare medical and surgical treatment of VUR.
Pilot Randomized, Placebo Controlled Trial to Investigate the Effect of Antibiotic Prophylaxis on the Rate of Urinary Tract Infection in Infants with Prenatal Hydronephrosis (Braga et al,2014)	Pilot study	The article is a pilot study related to an ongoing clinical trial. No results are available.
Pediatric Vesicoureteral Reflux Guidelines Panel Summary Report on the Management of Primary Vesicoureteral Reflux in Children (Elder et al, 1997)	Guidelines Summary	The article is an old version of AUA guideline on the management and screening of primary vesicoureteral reflux in children. The updated version was already included in our review.

Prophylactic cefdinir for pediatric cases of complicated urinary tract infection (Oishi et al, 2011)	Cohort observational study	The study investigated the pharmacokinetics and the efficacy of cefdinir in a small cohort of children with VUR or ureteropelvic junction stenosis. No control group was included. No comparison was made with alternative interventions.
Retrospective Study of Children with Acute Pyelonephritis (Ghiro et al, 2002)	Cohort observational study	The study analyzed a cohort of children who received antibiotic prophylaxis after an episode of acute pyelonephritis. No control group was included. No comparison was made with alternative interventions.
Efficacy and tolerability of long-term oral cefaclor therapy in the prevention of urinary tract infections in infants and children (Canepa et al, 1998)	Cohort observational study	The study analyzed a cohort of children who received antibiotic prophylaxis. No control group was included. No comparison was made with alternative interventions.
Outcome After Discontinuing Prophylactic Antibiotics in Children With Persistent Vesicoureteral Reflux (Kitchens et al, 2010)	Cross-sectional observational study	The study analyzed a cohort of children affected by VUR who discontinued antibiotic prophylaxis. No control group was included. No comparison was made with alternative interventions.
Outcome of antibiotic prophylaxis discontinuation in patients with persistent vescicoureteral reflux initially presenting with febrile urinary tract infeciton: time to event analysis (Leslie et al,2010)	Cross-sectional observational study	The study analyzed a cohort of children affected by VUR who discontinued antibiotic prophylaxis. No control group was included. No comparison was made with alternative interventions.
Renal damage in children randomized to prophylaxis, endoscopic injection, or surveillance results from the swedish reflux trial (Brandstrom et al, 2010)	Randomized clinical trial	The article analyzes the same data presented by the Swedish reflux trial that is already included in our review.
Selecting Children with Vesicoureteral Reflux Who are Most Likely to Benefit from Antibiotic Prophylaxis: Application of Machine Learning to RIVUR (Bertsimas et al, 2021)	Randomized clinical trial	The article analyzes the same data presented by the RIVUR trial that is already included in our review.
Renal Scarring in the Randomized Intervention for Children with Vesicoureteral Reflux (RIVUR) Trial (Mattoo et al, 2015)	Randomized clinical trial	The article analyzes the same data presented by the RIVUR trial that is already included in our review.
Prophylactic antibiotics in children at risk for urinary tract infection (Hellerstein et al, 2002)	Cohort observational study	The study analyzed a cohort of children who received antibiotic prophylaxis. No control group was included. No comparison was made with alternative interventions.
A Reanalysis of the RIVUR Trial Using a Risk Classification System. (Wang et al, 2018)	randomized clinical trial	The article analyzes the same data presented by the RIVUR trial that is already included in our review.
Cost-effectiveness of antimicrobial prophylaxis for children in the RIVUR trial. (Palmer et al, 2018)	randomized clinical trial	The article analyzes the same data presented by the RIVUR trial that is already included in our review.
The Swedish Reflux Trial in Children I Study Design and Study Population Characteristics (Brandstrom et al, 2010)	Randomized clinical trial	The article analyzes the same data presented by the Swedish reflux trial that is already included in our review.

Antimicrobial Resistance and Urinary Tract Infection Recurrence (Nelson et al, 2016)	Randomized clinical trial	The article analyzes the same data presented by the RIVUR trial that is already included in our review.
Intermittent trimethoprim-sulfamethoxazole in children with vesicoureteral reflux (Hori et al,1996)	Cohort observational study	The study analyzed a cohort of children who received antibiotic prophylaxis. No control group was included. No comparison was made with alternative interventions.
Can prophylactic antibiotics safely be discontinued in children with vescicoureteral reflux? (Al-Sayyad et al, 2005)	Cross-sectional observational study	The study analyzed a cohort of children affected by VUR who discontinued antibiotic prophylaxis. No control group was included. No comparison was made with alternative interventions.
Guidelines for management of children with urinary tract infection and and VUR, Recommendations from a Swedish state of-the art conference (Jodal et al, 1999)	Guidelines	Low methodological quality
Meta-analyses in prevention and treatment of urinary tract infections. (Masson et al, 2009)	Review of meta-analyses	The article reported the results of different systematic reviews and meta-analyses mainly involving adult women. The only study including children was already included in our study and individually analyzed.
Urinary tract infection in children (Larcombe et al, 2010)	Systematic review	Previous version of a systematic review already included in our review.
Urinary tract infection in children (Larcombe et al, 2007)	Systematic review	Previous version of a systematic review already included in our review.

CHARACTERISTICS AND RESULTS OF INCLUDED OBSERVATIONAL STUDIES, RANDOMIZED CLINICAL TRIALS, SYSTEMATIC REVIEWS, AND GUIDELINES WITH RESULTS OF RISK OF BIAS AND QUALITY ASSESSMENT

Should antibiotic prophylaxis be used in all children with a previous UTI?
P: children aged <18 years with a single previous UTI
I: long-term antibiotic prophylaxis with any molecule
C: no prophylaxis
O1: risk of UTI recurrence
O2: risk of new renal scars
O3: risk of new antimicrobial resistances
O4: risk of drug-related adverse events

Table S2. Characteristics and results of included studies

TITLE	DESIGN	POPULATION	INTERVENTIONS	OUTCOMES	RESULTS	RISK OF BIAS ASSESSMENT (RoB2 or NOS)
Recurrent urinary tract infections in children: risk factors and association with prophylactic antimicrobials (Conway et al, 2007)	Observational retrospective cohort study<br><br>Mean follow-up: 13 months	775 children aged 6 years or younger who were diagnosed with first UTI.<br><br>Data were extracted from a network of 27 primary care pediatric practices in the USA.<br><br>Children aged 6 years or younger who experienced a first UTI and with at least 2 clinic visits between July 1, 2001, and May 31, 2006 were included. Any child with	Age at first UTI, sex, race, VCUG results (categorized as "not performed," "normal," "VUR grade 1-3," or "VUR grade 4-5"), antibiotic prophylaxis or recent antibiotic exposure on a daily basis were investigated as possible risk factors for recurrent UTIs.<br><br>Each identified prescription,	Time to recurrent UTI.<br><br>Risk factors for recurrent UTI<br><br>Association between antimicrobial prophylaxis and recurrent UTI.<br><br>Risk factors for antimicrobial resistance.	In multivariable Cox time-to-event models, factors associated with increased risk of recurrent UTI included white race (0.17 per person-year; HR: 1.97; 95% CI: 1.22-3.16), age 3 to 4 years (0.22 per person-year; HR: 2.75; 95% CI: 1.37-5.51), age 4 to 5 years (0.19 per person-year; HR: 2.47; 95% CI: 1.19-5.12), and grade 4-5 VUR (0.60 per person-year; HR: 4.38; 95% CI: 1.26-15.29).<br><br>Recurrent UTI occurred in 19/128 (14.8%) children on prophylaxis and 64/483 (13.3%) children not exposed to prophylaxis. Antimicrobial resistance occurred more frequently in children receiving prophylaxis (89.5% vs 53.1%)	NOS: 8

		a history of previous UTIs was excluded.	blinded to the patient's outcome, was manually reviewed.		In multivariate analysis, antimicrobial prophylaxis was not associated with decreased risk of re-current UTI (HR: 1.01; 95% CI: 0.50-2.02), even after adjusting for propensity to receive prophylaxis, but it was a risk factor for antimicrobial resistance (HR: 7.50; 95% CI: 1.60-35.17).	
Long-term resistance trends of uropathogens and association with antimicrobial prophylaxis (Bitsori et al, 2014)	Observational retrospective cross-sectional study	638 children aged <15 years hospitalized for UTI during a study period of 12 consecutive years.<br><br>Surgical, oncology or intensive care patients were excluded.	Data on age, gender, preceding UTI episodes, VUR or other urological abnormalities, and current prophylaxis were recorded.<br><br>Prophylactic antimicrobials included cotrimoxazole (80 children, 61.5 %), cefaclor (27, 20.8 %), nitrofurantoin (16, 12.3 %), and amoxicillin (7, 5.4 %).	To identify long-term resistance trends of uropathogens.<br><br>To investigate the effect of antibiotic prophylaxis on the risk of new antimicrobial resistances.	Independent risk factors for resistance to each antibiotic, as confirmed by logistic regression analysis, were the use of any prophylaxis for resistance to cotrimoxazole ($p < 0.0001$) and nitrofurantoin ($p < 0.044$), the use of agent other than cotrimoxazole for resistance to cefuroxime ($p < 0.0007$), ceftriaxone ($p < 0.0004$), and gentamicin ($p <$ 0.0007), the male gender for resistance to amoxicillin ($p <0.007$), and non-E. coli pathogens for resistance to amoxicillin (p 0.0006), cefuroxime ($p <0.0001$), cotrimoxazole ($p <0.0001$), and nitrofurantoin ($p <0.0001$).	NOS: 7
Antibiotic resistance patterns in children hospitalized for urinary tract infections.<br>(Lutter et al, 2005)	Observational retrospective cohort study<br><br>Mean follow-up: 2.5 years	361 patients aged <18 years with a previous UTI including children with VUR.<br><br>Patients were excluded if they had received outpatient antibiotics (other than antibiotic prophylaxis) prior to admis- sion, had urologic stents in place, performed regular self-catheterization for spina bifida or other causes of neurogenic bladder	Data on age, gender, preceding UTI episodes, VUR or other urological abnormalities, and current prophylaxis were recorded.<br><br>Prophylactic antimicrobials included cotrimoxazole,, nitrofurantoin, and amoxicillin.	Risk factor for resistance to third generation-cephalosporins and aminoglycosides.	Resistance to cefotaxime sodium was 3% in the patients not receiving antibiotic prophylaxis, but was 27% in the children receiving prophylactic antibiotics (relative risk, 9.9; 95% confidence interval, 4.0-24.5; P<0.001).<br><br>Resistance to aminoglycoside antibiotics was 1% in the children not receiving prophylaxis and 5% in the children receiving prophylactic antibiotics.	NOS: 7

Antibiotic prophylaxis and recurrent urinary tract infection in children<br>(Craig et al, 2009)	Randomized clinical trial<br><br>Mean follow-up: 12 months	576 children aged <18 years who had had at least one microbiologically proven urinary tract infection were recruited in 4 centers in Australia.<br><br>Children with a known neurologic, skeletal, or urologic predisposing cause or with a known contraindication to trimethoprim–sulfamethoxazole therapy were ineligible.	Patients were randomly assigned to receive either daily trimethoprim–sulfamethoxazole suspension (as 2 mg of trimethoprim plus 10 mg of sulfamethoxazole per kg of body weight) or placebo for 12 months.	Recurrence of symptomatic or febrile UTI.<br><br>Antimicrobial resistance of breakthrough UTIs.<br><br>Deterioration in cortical scintigraphy at 12 months.<br><br>Drug-related adverse events.	During the study, urinary tract infection developed in 36 of 288 patients (13%) in the group receiving trimethoprim-sulfamethoxazole (antibiotic group) and in 55 of 288 patients (19%) in the placebo group (HR: 0.61; 95% CI: 0.40-0.93; p=0.02 by the log-rank test).<br><br>In the antibiotic group, the reduction in the absolute risk of urinary tract infection (6 percentage points) appeared to be consistent but not statistically significant across all subgroups of patients (P≥0.20 for all interactions).<br><br>The progression of abnormal results on renal scanning from baseline to follow-up did not differ significantly between the antibiotic group and the placebo group.<br><br>Fewer hospitalizations and adverse drug reactions occurred in the antibiotic group than in the placebo group, but the differences were not significant.	RoB2:<br>-Risk of UTI recurrence: low<br>-Risk of new renal scars: low<br>-Risk of antimicrobial resistances: low<br>-Risk of drug-related adverse events: some concerns
Prophylaxis after first febrile urinary tract infection) in children? a multicenter, randomized, controlled, noninferiority trial<br>(Montini et al, 2007)	Randomized clinical trial<br><br>Mean follow-up: 12 months	338 children aged <7 years were enrolled after a first episode of febrile UTI. 309 with a confirmed pyelonephritis on a technetium ^{99m}Tc-DMSA scan with or without VUR and 27 with a clinical pyelonephritis.<br><br>Patients were enrolled in different centers in Italy.<br><br>Exclusion criteria were complex urologic	Patients were randomly assigned to receive either prophylaxis (co-trimoxazole 15 mg/kg per day or co-amoxiclav 15 mg/kg per day) or no prophylaxis for 12 months.	Recurrence of febrile UTI.<br><br>Renal scarring on technetium 99m DMSA scan after 12 months.<br><br>Drug-related adverse events.	Intention-to-treat analysis showed no significant differences in the rate of UTI recurrence between no prophylaxis (12/127; 9.45%) vs. prophylaxis (15/211; 7.11%).<br><br>In the subgroup of children with reflux, the recurrence of febrile urinary tract infections was 9 (19.6%) of 46 on no prophylaxis and 10 (12.1%) of 82 on prophylaxis.<br><br>No significant difference was found in the secondary outcome (renal scarring): 2 (1.9%) of 108 children not on prophylaxis vs. 2 (1.1%) of 187 children on prophylaxis.	RoB2:<br>-Risk of UTI recurrence: low<br>-Risk of new renal scars: low<br>-Risk of drug-related adverse events: some concerns

		malformations and/or severe renal damage			Bivariate analysis and Cox proportional hazard model showed that grade III reflux was a risk factor for recurrent febrile urinary tract infections. The lack of prophylaxis was not a risk factor.<br><br>Twenty-five (7.3%) children experienced minor adverse effects during the 12 months of follow-up. All patients were on prophylaxis: 15 on co-amoxiclav and 10 on co-trimoxazole.	
Clinical significance of primary vesicoureteral reflux and urinary antibiotic prophylaxis after acute pyelonephritis: a multicenter, randomized, controlled study.<br>(Garin et al, 2006)	Randomized clinical trial<br><br>Mean follow-up: 12 months	218 children aged <18 years enrolled in the USA, Chile, and Spain after a first episode of acute pyelonephritis confirmed through a ^{99m}Tc-DMSA scan, with or without low-grade VUR.<br><br>Exclusion criteria were the presence of grade IV or V VUR, neurogenic bladder, posterior urethral valves, uri- nary diversion, bladder diverticulum, ureterocele, renal failure, and pregnancy.<br>.	Patients were randomly assigned to receive either prophylaxis (sulfamethoxazole/trimethoprim 1–2 mg/kg of trimethoprim or nitrofurantoin 1.5 mg/kg once daily) or no prophylaxis for 12 months.	Recurrence of febrile UTI.<br><br>Renal scarring on technetium 99m DMSA scan after 12 months.	The overall incidence of recurrent UTI was 20.1%.<br><br>Among 100 children in prophylaxis, 17 (17%) experienced UTI recurrences versus 27/118 (22.9%) children not on prophylaxis (p 0.28).<br><br>Among patients not receiving urinary antibiotic prophylaxis, the incidence of 22.4% for those with VUR was not significantly different from the 23.3% for those without VUR (p 0.9999). Among children receiving urinary antibiotic prophylaxis, the recurrence rate of 8.8% for patients without VUR was not significantly different from the recurrence rate of 23.6% for those with VUR (p 0.0633).<br><br>Only 13 (5.9%) of the 218 patients developed renal scars during the 1 year of follow-up monitoring. Similar rates of scarring were found for patients who received prophylaxis and those who did not (7% vs 5.1%).	RoB2:<br>-Risk of UTI recurrence: some concerns<br>-Risk of new renal scars: some concerns

Figure S1. Risk of bias 2 results for randomized clinical trials investigating the risk of UTI recurrence.

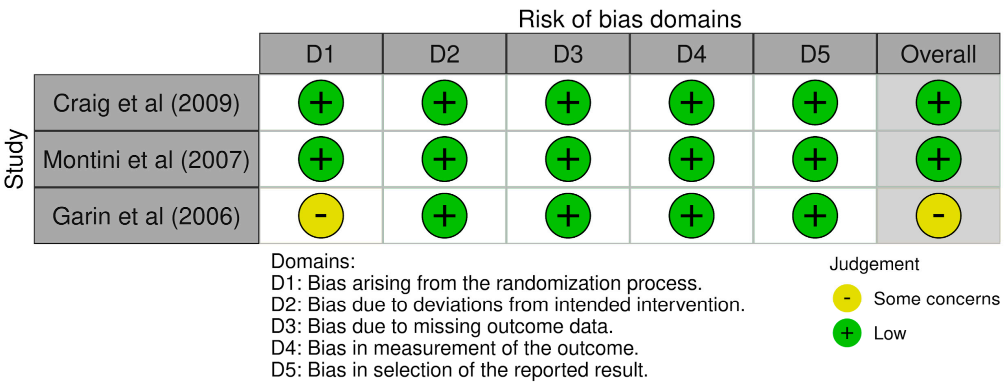

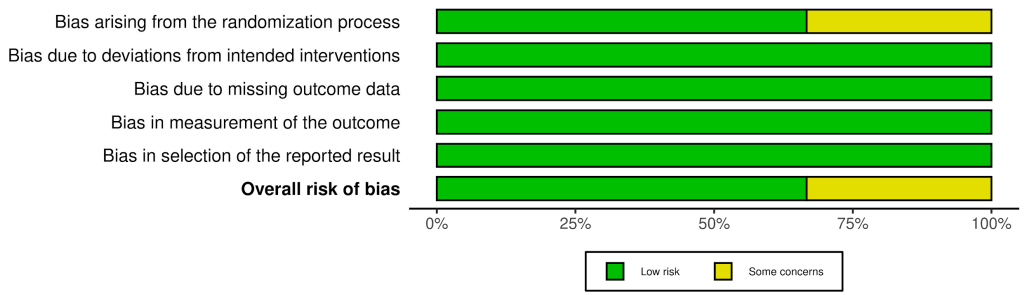

Figure S2. Risk of bias 2 results for randomized clinical trials investigating the risk of new renal scars.

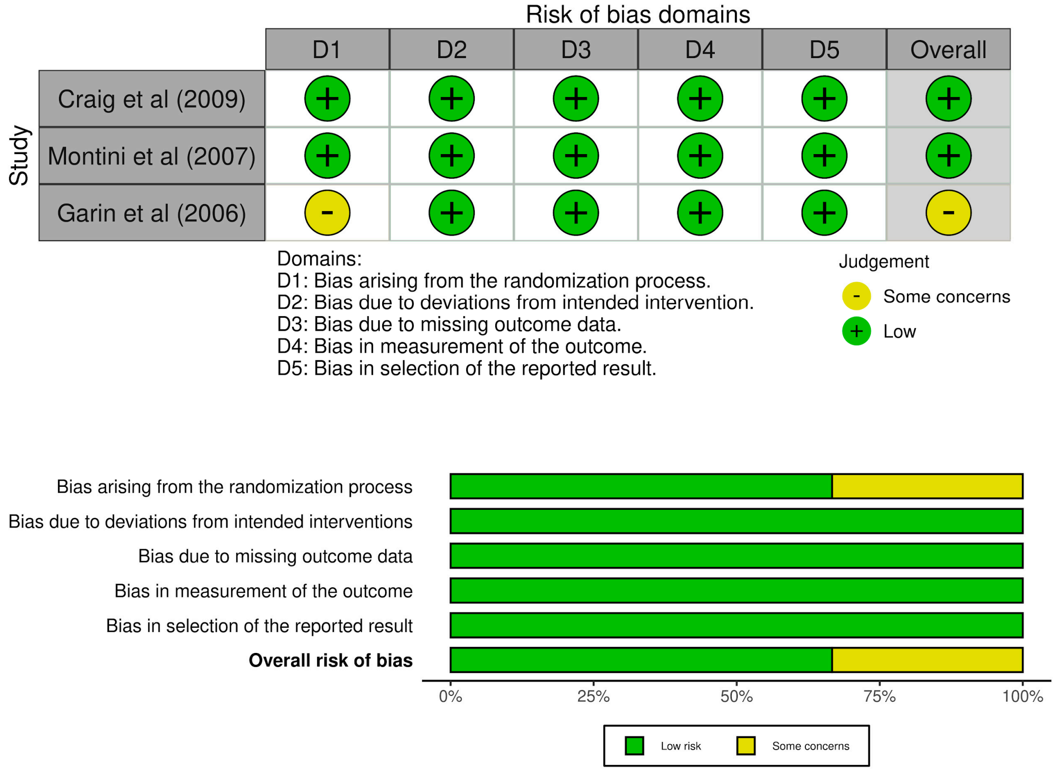

Figure S3. Risk of bias 2 results for randomized clinical trials investigating the risk of new antimicrobial resistances.

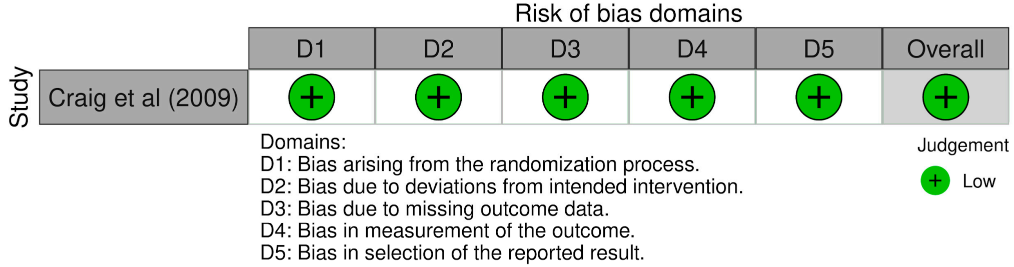

Figure S4. Risk of bias 2 results for randomized clinical trials investigating the risk of drug-related adverse events.

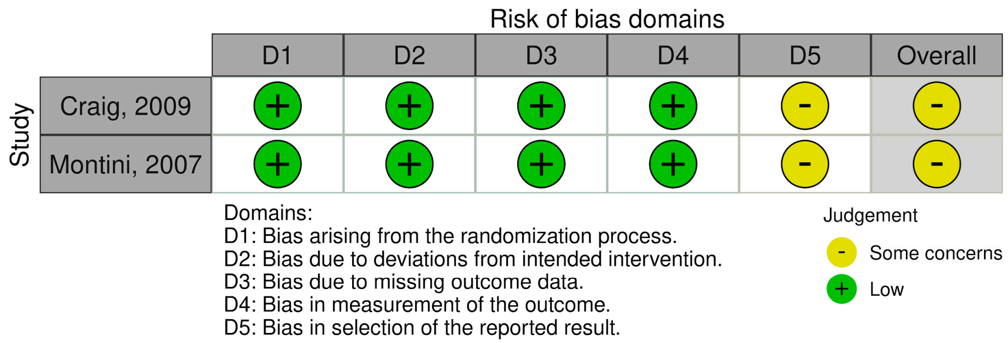

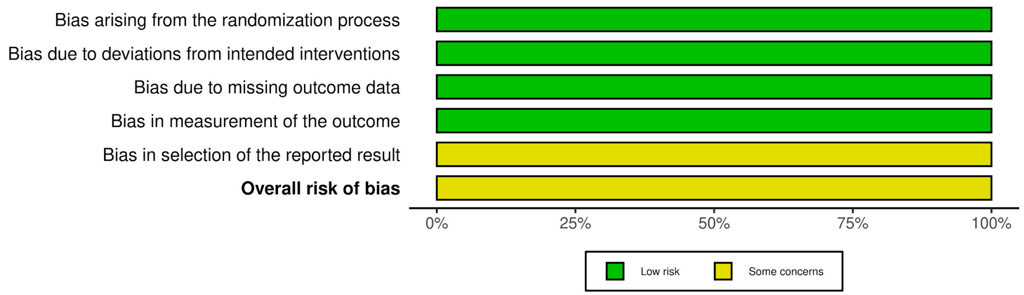

Table S3. Characteristics and results of included systematic reviews.

TITLE	POPULATION, INTERVENTIONS AND OUTCOMES	QUALITY ASSESSMENT (AMSTAR2)	RESULTS	CONCLUSIONS
Antibiotic prophylaxis following urinary tract infection in children: a systematic review of randomized controlled trials (Mathew et al, 2010)	1475 children (< 18 years of age) from 5 randomized clinical trials. Two trials included only children with VUR; one enrolled all participants after an episode of acute pyelonephritis.<br><br>All studies used trimethoprim-sulfamethoxazole; three studies also included amoxicillin-clavulanic acid or	Moderate	- Meta-analysis showed that risk of UTI recurrence was reduced with antibiotic prophylaxis when all children (with VUR, without VUR and unknown status) were considered together (RR=0.73; CI=0.56-0.95; 3 trials; 1132 participants. I^2=0%).<br><br>- However, there was no benefit of prophylaxis when children without VUR (RR=0.72; CI=0.43-1.20; 3 trials; 549 participants: I^2=0%) were examined separately.	- Current evidence is unable to identify subgroup(s) of children who may benefit from antibiotic prophylaxis.<br><br>- It is possible that the balance between benefit and harm of antimicrobial prophylaxis in children at greater risk of complications is different from those included in

	nitrofurantoin. Only one trial was placebo-controlled.<br><br>Main outcomes were UTI recurrence, renal scarring, long-term complications, and antimicrobial resistances.		- Antibiotic prophylaxis did not prevent new or worsening renal scarring in children with VUR (RR=2.64; CI=0.53-13.03; 1 trial; 113 participants), without VUR (RR=0.67; CI=0.13-3.48; 1 trial; 105 participants) and both groups combined (RR=1.00; CI=0.49-2.03; 3 trials; 667 participants; I^2=0%).<br><br>- The increased risk of adverse events with antibiotics was not statistically significant (RR=3.08; CI=0.02-549.95; 2 trials; 914 participants; I^2=92%).<br><br>- The higher risk of antimicrobial resistances with antibiotic prophylaxis was not statistically significant (RR=8.60; CI=0.86-85.81; 3 trials; 190 participants; I^2=82%).	clinical trials, necessitating individualized decisions.<br><br>- Lack of compliance could apparently reduce the beneficial effect of prophylaxis. Whereas, better compliance during clinical trials could suggest greater benefit than in real life (efficacy versus effectiveness).
Antibiotic prophylaxis for children at risk of developing urinary tract infection: a systematic review. (Mori et al, 2009)	The study population included 656 children aged 0–18 years who had had a UTI. A total of 8 trials were included in the review. The trials were stratified by the four population categories:<br><br>- Children who had symptomatic UTI including those with and without VUR (2 trials).<br><br>- Children who had symptomatic UTI without VUR (2 trials).<br><br>- Children who had symptomatic UTI with VUR (3 trials).<br><br>- Children with VUR (1 trial).<br><br>Intervention and comparison were antibiotic prophylaxis vs no prophylaxis. The primary outcomes were incidence of new or progressive renal scarring or recurrence of pyelonephritis.	Critically low	- There was no evidence of difference in recurrence of symptomatic UTI in the overall population nor in any of the subgroup analyses between the intervention and control groups. The summary RR of all the groups was 0.96 (four trials, 95% CI: 0.69–1.32).<br><br>- There was no evidence of a difference in incidence of new or progressive renal scarring in the overall population nor in any of the subgroups. The summary RR was 1.15 (three trials, 95% CI: 0.75–1.78).	- There is no evidence of a reduction in the incidence of symptomatic UTI nor in the prevalence of renal scarring.<br><br>- Prophylaxis is inconvenient for the patient, adherence is poor, it carries the risks associated with any medication and patients may become colonized with resistant organisms.
Long-term antibiotics for the prevention of recurrent urinary tract infection in children: a systematic review and meta-analysis.	Eleven clinical trials with 2046 patients were included. All but one of the studies included patients with VUR. The treatment regimen was usually trimethoprim-sulfamethoxazole or nitrofurantoin, but the dose and duration varied among studies.	Low	- Recurrent symptomatic UTI was reported by seven of the 11 studies including 1717 patients. Recurrent symptomatic UTI was not significantly reduced by antibiotic prophylaxis (RR 0.83, 95% CI 0.66 to 1.05, p=0.13).<br><br>- The duration of treatment did not affect prophylactic efficacy (RR for trials with prophylactic time <12 months 1.93,	- The long-term antibiotic prophylaxis in children was not associated with significant prevention of recurrent symptomatic UTI or new renal damage.

(Dai et al, 2010)	To evaluate the effectiveness and safety of long-term prophylactic antibiotics in terms of recurrent UTI and renal scars.		95% CI 0.63 to 5.92, p=0.25 vs RR for trials with prophylactic time ≥12 months 0.81, 95% CI 0.64 to 1.04, p=0.09; test for subgroup differences p=0.14, I2=54%).<br><br>- The rate of new or deteriorated renal scars was reported by seven studies and showed no significant difference between antibiotic prophylaxis and placebo/no treatment (RR 0.95, 95% CI 0.51 to 1.78, p=0.87).<br><br>- Among studies included, the incidence of bacterial resistance ranged from 67% to 100% in the prophylaxis groups and from 0% to 39% in the non-prophylaxis groups.	- Long-term antibiotics promote the development of resistant bacteria.
Technical report—diagnosis and management of an initial uti in febrile infants and young children<br>(Finnell et al, 2011)	8 randomized clinical trials including children aged <18 years with and without VUR. 6 studies compared antimicrobial prophylaxis with no prophylaxis.<br><br>The grade of VUR among the enrolled children varied from 0 to V.<br><br>The antimicrobial agents used were trimethoprim-sulfamethoxazole, amoxicillin-clavulanic acid or nitrofurantoin.	High	- Among all studies, there was no significant difference in rates of recurrence of pyelonephritis for children who received antimicrobial therapy and those who did not (RR of 0.77; 95% CI: 0.47-1.24).<br><br>- When considering only children without VUR, there was no significant difference in rates of recurrence of pyelonephritis with RR of 0.62 (95% CI: 0.30-1.27).<br><br>- There was no significant difference in rates of recurrence of any type of UTI for children without VUR who received antimicrobial agents and those who did not with RR of 0.72 (95% CI: 0.43-1.20).<br><br>- The antimicrobial resistance patterns of the pathogens isolated during UTI recurrences were assessed in 5 of the RCTs included in the meta-analysis. The proportions of resistant bacteria ranged from 0% to 39%; in the antimicrobial prophylaxis groups, the proportions of resistant bacteria ranged from 53% to 100%.	- Antimicrobial prophylaxis does not seem to significantly reduce the rates of recurrence of pyelonephritis.<br><br>- Even if additional studies were to show a statistically significant effect of prophylaxis for pyelonephritis, the RR would be 0.80, corresponding to a reduction in RR of 20%. If we consider the prevalence of VUR, the risk of recurrent UTI in those children, and this modest potential effect, we can determine that 100 children would need to undergo VCUG for prevention of 1 UTI in the first year.<br><br>- UTI recurrences with resistant bacteria were more common in the groups of children assigned randomly to receive antimicrobial prophylaxis.
Urinary tract infection in children: recurrent infections<br>(Larcombe et al, 2013)	Two systematic reviews and one RCT were included in this updated systematic review.<br><br>- A: The first systematic review included studies if the majority of children (>50%) did not have a renal tract abnormality, or a major neurological, urological, or muscular disease. It included six RCTs (1069	Critically low	A<br>- Antibiotics did not appear to reduce the risk of symptomatic UTI compared to placebo/no treatment (4 RCTs,1024 children, RR 0.75, 95% CI 0.36-1.53, P = 0.43; significant heterogeneity, I^2= 62%, P = 0.05).<br><br>- Prophylactic antibiotic did not appear to reduce the risk of symptomatic UTI in children with VUR (2 RCTs, 371	- Recent, well-conducted RCTs suggest a limited benefit of prophylaxis: 12 to 13 children need to be treated for 1 year to prevent one symptomatic UTI.<br><br>- Prophylactic antibiotics increase antimicrobial resistance, and many

	children) comparing prophylactic antibiotics with placebo or no treatment.<br><br>- B: The second systematic review included only children with primary VUR. It involved eight RCTs (1039 children), six comparing antibiotic prophylaxis with no treatment and two versus placebo.<br><br>- C: One RCT, involving 176 children with spina bifida undergoing clean intermittent catheterisation and continuing or discontinuing low-dose prophylactic antibiotics.		children, RR 0.65, 95% CI 0.39-1.07, P = 0.088) compared to those without VUR (3 RCTs, 491 children, RR 0.56, 95% CI 0.15-2.12, P = 0.40; heterogeneity, I^2 = 62%, P = 0.07).<br><br>- No difference was found between antibiotics and placebo/no treatment (2 RCTs, 914 children, RR 2.31, 95% CI 0.03-170.67, P = 0.70; significant heterogeneity, I^2 = 88%, P = 0.004).	breakthrough UTIs are caused by resistant organisms.<br><br>- There is no evidence comparing different molecules or different durations of antibiotics.
Antibiotic prophylaxis for urinary tract infection-related renal scarring: a systematic review. (Hewitt et al, 2017)	Seven randomized clinical trials (1427 subjects) were included in the meta-analysis that studied the effect on UTI-related renal scarring of antibiotic prophylaxis versus no prophylaxis or placebo.<br><br>Overall population included children aged <18 years with a previous symptomatic or febrile UTI with or without VUR. 99mTc dimercaptosuccinic acid scans were performed at entry into the studies and at the end of follow-up (12-24 months) to detect new scar formation.	High	- Meta-analyses did not show differences in the incidence of new scarring between the prophylaxis and no prophylaxis groups in the overall population (pooled RR, 0.83; 95% CI, 0.55-1.26).	- The lack of influence of prophylaxis on scarring is confirmed by this meta-analysis, which did not demonstrate any benefit, despite the combined studies documenting 1068 patient-years of antibiotic prophylaxis.
Antibiotics for the prevention of urinary tract infection in children: a systematic review of randomized controlled trials. (Williams et al, 2001)	Five trials involving 463 children aged <18 years with or without VUR and who did not have a major predisposing cause such as a major neurologic or obstructive disease.<br><br>The objective was to evaluate the effectiveness of low-dose, long-term antibiotics versus no prophylaxis for the prevention of symptomatic UTI.<br><br>Antibiotics were given for 2 to 12 months and included trimethoprim-sulfamethoxazole, cotrimoxazole, and nitrofurantoin.	Critically Low	- Long-term antibiotic administration reduced the risk of UTI in the overall population (RR 0.31, 95% CI 0.10-1.00), but there was significant heterogeneity (Q = 13.45, P < .01), and there was no sustained benefit once antibiotics had ceased (relative risk 0.79, 0.61 to 1.02).	- There is considerable uncertainty about whether long-term, low-dose antibiotic administration prevents UTI in children.

Evaluating the benefits of antimicrobial prophylaxis to prevent urinary tract infections in children: a systematic review (Le Saux et al, 2001)	6 trials included children aged <18 years with at least one previous UTI. 3 trials dealt with children who had normal urinary tracts, and 3 included children with VUR or neurogenic bladder.<br><br>Interventions were antibiotic prophylaxis compared to no prophylaxis or placebo. The outcome of interest was the recurrence of UTI.	Low	- The rate of infections for patients with normal urinary tracts ranged from 0 to 4.0 per 10 patient-years for the treatment groups and from 4.0 to 16.7 for the control groups.	- Because the magnitude of benefit of prophylactic antimicrobials may be small and a potential for harboring resistant bacteria may exist, they should be used only after careful consideration and only after attempts have been made to correct conditions that predispose to urinary stasis (e.g., voiding dysfunction or constipation).
Long-term antibiotics for preventing recurrent urinary tract infection in children.<br>(Williams et al, 2019)	In this Cochrane updated systematic review, 16 studies (2036 children randomized in clinical trials and 1977 not randomized) were included and .<br><br>Children less than 18 years of age who were at risk of recurrence due to prior infection were included. Studies were included if the majority of participants (> 50%) did not have a predisposing cause such as a renal tract abnormality, including VUR, or a major neurological, urological or muscular disease.<br><br>To assess whether long-term antibiotic prophylaxis was more effective than placebo/no treatment in preventing recurrence of UTI in children, and if so which antibiotic in clinical use was the most effective.	High	- Compared to placebo/no treatment, antibiotics lead to a modest decrease in the number of repeat symptomatic UTI in children; however, the estimate from combining all studies was not certain and the confidence interval indicates low precision indicating that antibiotics may make little or no difference to risk of repeat infection (RR 0.75, 95% CI 0.28-1.98).<br><br>- The estimated reduction in risk of repeat symptomatic UTI for children taking antibiotics was similar and not statistically significant in children with VUR (RR 0.65, 95% CI 0.39 to 1.07) compared to those without VUR (RR 0.56, 95% CI 0.15 to 2.12).<br><br>- Data for antibiotic resistance with the analysis estimating the risk of a UTI caused by a bacteria resistant to the prophylactic antibiotic being almost 2.5 times greater in children on antibiotics than for children on placebo or no treatment (RR 2.40, 95% CI 0.62 to 9.26). However, the confidence interval is wide, showing imprecision.	- This review found that long-term antibiotics may reduce the risk of repeat symptomatic infections but the benefits are probably small and must be weighed against the likelihood of antimicrobial resistances.<br><br>- Long-term, low dose antibiotics to prevent repeat UTI should be reserved for those children at higher risk of repeat infection, such as young infants, and children's clinicians would strongly want to reduce the risk of further infections, such as children with renal abnormalities.

Table S4. Characteristics and recommendations of included guidelines

GUIDELINES			RECOMMENDATIONS	GRADING
2019	Updated Italian recommendations for the diagnosis, treatment and follow-up of the first febrile urinary tract infection in young children	Italian Society for pediatric nephrology	a. Antibiotic prophylaxis is not routinely recommended in infants and children after the first febrile UTI.<br>b. Prophylaxis does not reduce the appearance and progression of permanent renal damage.	a. grade A<br>b. not specified

2020	Swiss consensus recommendations on urinary tract infections in children	Buettcher et al	a. In general, antibiotic prophylaxis is not recommended.<br>b. In the following circumstances, antibiotic prophylaxis may be indicated (planned duration should be documented):<br>– Children with complex CAKUT or with underlying bladder dysfunction (only after interdisciplinary—pediatric nephrology/urology/infectious diseases—review)<br>– Children with high-grade VUR (WHO grades IV and V)<br>– If micturition cystourethrogram is indicated, antibiotic prophylaxis may be started and continued until the time of the examination	a. Grade A<br>b. Not specified
2015	KHA-CARI guideline: Diagnosis and treatment of urinary tract infection in children	KHA/CARI	a. We do not recommend the routine use of prophylactic antibiotics for children after a first UTI.<br>b. Some children at high risk of morbidity relating to further UTI may benefit from the use of prophylactic antibiotics. (ungraded)	a. Grade 1A
2021	Update of the EAU/ESPU guidelines on urinary tract infections in children	EAU/ESPU	a. Offer long-term antibacterial prophylaxis in case of high susceptibility to UTI and risk of acquired renal damage and lower urinary tract symptoms<br><br>With increasing resistance rates, one should carefully consider which patients should receive antibacterial prophylaxis, since long-term use has been associated with increased microbial resistance. Its use causes a reduction in the number of recurrent UTIs, but it did not reduce newly acquired renal damage in children with first and second UTI. However, when used in children with anatomic abnormalities of the urinary tracts a reduction in UTI and subsequent renal scarring was shown.	a. Grade 1b; strong

Should antibiotic prophylaxis be used in all children with recurrent UTIs?
P: children aged <18 years with recurrent UTIs
I: long-term antibiotic prophylaxis with any molecule
C: no prophylaxis
O1: risk of UTI recurrence
O2: risk of new renal scars
O3: risk of new antimicrobial resistances
O4: risk of drug-related adverse events

Table S5. Characteristics and results of included studies

TITLE	DESIGN	POPULATION	INTERVENTIONS	OUTCOMES	RESULTS	RISK OF BIAS ASSESSMENT (RoB2 or NOS)
Antimicrobial Resistance of Breakthrough urinary tract infections (Nomura et al, 2017)	Observational retrospective case-control study.	37 children (41 cases) aged <5 years treated for recurrent UTI and requiring hospitalization, with or without VUR<br><br>Recurrent UTI was defined as contracting UTI, recovering completely, then contracting UTI again.<br><br>Exclusion criteria were the use of multiple prophylactic agents and surgical treatments.	31 cases had recurrent UTIs treated without prophylaxis, whereas 10 received prophylaxis (5 received trimethoprim-sulfamethoxazole and 5 received cefaclor).	Rate of antimicrobial resistance to empiric treatments in breakthrough infections.	Resistance rates to empiric treatments in breakthrough infections were higher but not statistically significant for children with prophylaxis than for those who did not receive prophylaxis (40.0% vs 25.9%, p >0.30).<br><br>The inappropriate treatment rate for children who received prophylaxis with cefaclor was the worst among the three groups (60.0%).	NOS: 7
Antibiotic prophylaxis and recurrent urinary	Randomized clinical trial	576 children aged <18 years who had had at least one	Patients were randomly assigned to receive either daily trimethoprim–	Recurrence of symptomatic or febrile UTI.	Among the subgroup of children with recurrent UTIs, a breakthrough infection occurred in 15/54 (28%) of children on	RoB2:<br>-Risk of UTI

tract infection in children (Craig et al, 2009)	Mean follow-up: 12 months	microbiologically proven urinary tract infection were recruited in 4 centers in Australia. 98 children with recurrent UTIs.<br><br>Children with a known neurologic, skeletal, or urologic predisposing cause or with a known contraindication to trimethoprim–sulfamethoxazole therapy were ineligible.	sulfamethoxazole suspension (as 2 mg of trimethoprim plus 10 mg of sulfamethoxazole per kg of body weight) or placebo for 12 months.		prophylaxis and in 16/44 (38%) of children not on prophylaxis (p 0.59; HR: 0.65, 95% CI: 0.32-1.32). Differences were not statistically significant.	recurrence : low

Figure S5. Risk of bias 2 results for randomized clinical trials investigating the risk of UTI recurrence.

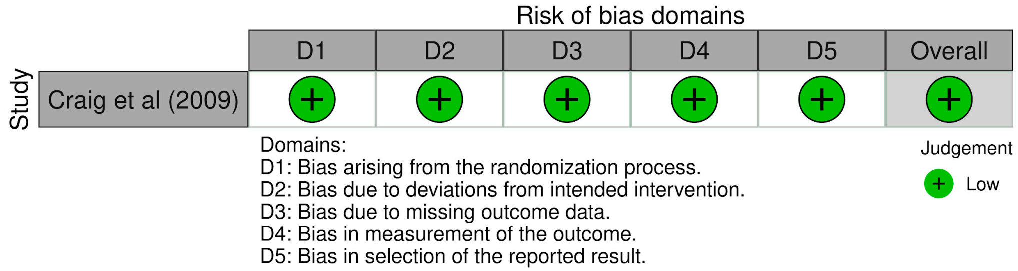

Table S6. Characteristics and results of included systematic reviews.

TITLE	POPULATION, INTERVENTIONS AND OUTCOMES	QUALITY ASSESSMENT (AMSTAR2)	RESULTS	CONCLUSIONS
Current status of long-term antibiotic prophylaxis for urinary tract infections in children: an antibiotic stewardship challenge (Alsubaie et al, 2019)	A total of 34 RCTs, 9 systematic reviews, and 3 guidelines describing the efficacy of antibiotic prophylaxis for preventing recurrences in pediatric patients with more than one previous UTI.	Critically Low	- Results from a systematic review and meta-analysis revealed that antibiotic prophylaxis may reduce the risk of recurrent symptomatic UTI in children who have had one or more previous UTIs, but the benefit is not statistically significant (RR, 0.75; 95% CI, 0.28-1.98).<br><br>- Additionally, a 2.5-fold higher threat of developing an antibiotic-resistant infection was observed in children receiving antibiotic prophylaxis (RR, 2.40; 95% CI, 0.62-9.26) was reported, although not statistically significant.	- The efficacy of prophylaxis for preventing recurrent UTI remains unclear due to non-generalizability of results obtained from sub optimally designed clinical trials.

Table S7. Characteristics and recommendations of included guidelines

GUIDELINES			RECOMMENDATIONS	GRADING
2019	Updated Italian recommendations for the diagnosis, treatment and follow-up of the first febrile urinary tract infection in young children	Italian Society for pediatric nephrology	a. Antibiotic prophylaxis may be considered in infants and children with recurrent febrile UTIs, defined as >3 febrile UTIs within 12 months.	a. Grade C
2018	Urinary tract infection (recurrent): antimicrobial prescribing	NICE	a. Taking account of the benefits and harms of antibiotic prophylaxis and the need to minimize antimicrobial resistance, the committee agreed that antibiotic prophylaxis could be considered in people aged 16 years and over with recurrent UTI, but only after other management options had been unsuccessful (behavioural and personal hygiene measures, managing any triggers and using non-antimicrobial treatments), if appropriate.<br>b. Taking account of the uncertainty in the evidence and the need to minimise antimicrobial resistance from long-term antibiotic use, the committee agreed that antibiotic prophylaxis could be considered in children and young people under 16 years, but only under specialist advice when other management options have been unsuccessful. This would be an individualised decision following an assessment of underlying causes, taking into account the severity and frequency of previous symptoms and the risk of developing complications.	a. not specified<br>b. not specified

2021	Update of the EAU/ESPU guidelines on urinary tract infections in children	EAU/ESPU	a. Offer long-term antibacterial prophylaxis in case of high susceptibility to UTI and risk of acquired renal damage and lower urinary tract symptoms<br><br>With increasing resistance rates, one should carefully consider which patients should receive antibacterial prophylaxis, since long-term use has been associated with increased microbial resistance. Its use causes a reduction in the number of recurrent UTIs, but it did not reduce newly acquired renal damage in children with first and second UTI. However, when used in children with anatomic abnormalities of the urinary tracts a reduction in UTI and subsequent renal scarring was shown.	a. Grade 1b; strong

Should antibiotic prophylaxis be used in children with VUR (any grade)?
P: children aged <18 years with VUR (any grade)
I: long-term antibiotic prophylaxis with any molecule
C: no prophylaxis
O1: risk of UTI recurrence
O2: risk of new renal scars
O3: risk of new antimicrobial resistances
O4: risk of drug-related adverse events

Table S8. Characteristics and results of included studies

TITLE	DESIGN	POPULATION	INTERVENTIONS	OUTCOMES	RESULTS	RISK OF BIAS ASSESSMENT (RoB2 or NOS)
Observation of patients with vesicoureteral reflux off antibiotic prophylaxis: physician bias on patient selection and risk factors for recurrent febrile urinary tract infection (Drzewiecki et al, 2012)	Observational retrospective cohort study.	529 children aged <18 years with primary VUR.<br><br>Patients with prior surgical correction were excluded from analysis.	Patients on antibiotic prophylaxis were compared with patients off continuous prophylaxis.	Risk factors for UTI recurrence.	Patients off continuous antibiotic prophylaxis tended to be older ($p < 0.001$), to be older at diagnosis ($p < 0.001$), to have an initial presentation other than febrile urinary tract infection ($p = 0.05$), to have non-dilating vesicoureteral reflux on most recent cystogram ($p < 0.001$) and to have lower bladder/bowel dysfunction scores if toilet trained ($p < 0.001$).	NOS: 9

					A recurrent UTI occurred in 19/224 (8.5%) children off prophylaxis vs 60/305 (19.5%) in children still on prophylaxis (p <0.001)	
Girls and renal scarring as risk factors for febrile urinary tract infection after stopping antibiotic prophylaxis in children with vesicoureteral reflux.<br>(Nakamura et al, 2020)	Observational retrospective before and after study.	144 children aged <10 years with primary VUR.<br><br>Patients with prior surgical correction were excluded from analysis.	Patients with persistent VUR were studied before and after discontinuing antibiotic prophylaxis.	Risk factors for UTI recurrence.	Among 144 children (99 boys and 45 girls), UTI developed in 34.<br><br>Recurrent UTI before stopping prophylaxis occurred in 20/144 (13.9%). After stopping prophylaxis, UTI occurred in 34/144 (23.6%; p 0.03)<br><br>The 5-year UTI-free rate after discontinuation of CAP was 69.4%.<br><br>On multivariate analyses, girls (p=0.008) and abnormalities on nuclear renal scans (p=0.0019), were the only significant factors for fUTI.	NOS: 7
Antibiotic prophylaxis for the prevention of recurrent urinary tract infection in children with low grade vesicoureteral reflux- results from a prospective randomized study (Roussey-Kesler et al, 2008)	Randomized clinical trial<br><br>Mean follow-up: 18 months	225 children aged <3 years with VUR enrolled in different centers in France.<br><br>Exclusion criteria were abnormal renal echography, obstructive uropathy, and allergy to sulfonamide.	Patients were randomly assigned to receive daily co-trimoxazole or no treatment and were followed for 18 months	Risk of UTI recurrence.	There was no significant difference in the occurrence of UTI between the two groups (17% vs 26%, p=0.2).<br><br>Prophylaxis significantly reduced urinary tract infection only in boys (p=0.013), most notably in boys with grade III vesicoureteral reflux (p=0.042).	RoB2:<br>-Risk of UTI recurrence: some concerns
Antimicrobial prophylaxis for children with vesicoureteral reflux<br>(Hoberman et al, 2014)	Randomized clinical trial<br><br>Mean follow-up: 24 months	607 children aged 2-71 months with VUR (grade I-IV) diagnosed after a febrile or symptomatic UTI. Children were enrolled in different centers in the USA.<br><br>Children with coexisting urologic anomalies, contraindications for the use of trimethoprim–	Children were randomly assigned to receive trimethoprim–sulfamethoxazole or placebo with double-blind administration.	Recurrence of symptomatic or febrile UTI.<br><br>Antimicrobial resistance of breakthrough UTIs.<br><br>Deterioration in cortical scintigraphy.<br><br>Drug-related adverse events.	Recurrent urinary tract infection developed in 39 of 302 children who received prophylaxis as compared with 72 of 305 children who received placebo (relative risk, 0.55; 95% confidence interval [CI], 0.38 to 0.78).<br><br>Prophylaxis reduced the risk of recurrences by 50% (HR: 0.50; 95% CI: 0.34 to 0.74) and was particularly effective in children whose index infection was febrile (HR: 0.41; 95% CI: 0.26 to 0.64) and in those with baseline	RoB2:<br>-Risk of UTI recurrence: low<br>-Risk of new renal scars: low<br>-Risk of antimicrobial

		sulfamethoxazole, or certain medical conditions were excluded.			bladder and bowel dysfunction (HR: 0.21; 95% CI: 0.08 to 0.58).<br><br>The occurrence of renal scarring did not differ significantly between the prophylaxis and placebo groups (11.9% vs 10.2%).<br><br>Among 87 children with a first recurrence the proportion of isolates that were resistant to trimethoprim–sulfamethoxazole was 63% in the prophylaxis group and 19% in the placebo group (p <0.0001).<br><br>Rates of drug-related adverse events were not significantly different between groups (50.7% vs 54.1%).	resistances: low<br>-Risk of drug-related adverse events: low
Antibiotic prophylaxis and recurrent urinary tract infection in children<br>(Craig et al, 2009)	Randomized clinical trial<br><br>Mean follow-up: 12 months	576 children aged <18 years who had had at least one microbiologically proven urinary tract infection were recruited in 4 centers in Australia.<br><br>243 children with any grade VUR.<br><br>Children with a known neurologic, skeletal, or urologic predisposing cause or with a known contraindication to trimethoprim–sulfamethoxazole therapy were ineligible	Patients were randomly assigned to receive either daily trimethoprim–sulfamethoxazole suspension or placebo for 12 months.	Recurrence of symptomatic or febrile UTI.	Among the subgroup of children with VUR, a breakthrough infection occurred in 14/122 (11.5%) of children on prophylaxis and in 21/121 (17.3%) of children not on prophylaxis (p 0.70; HR: 0.65, 95% CI: 0.28-1.52). Differences were not statistically significant.	RoB2:<br>-Risk of UTI recurrence: low
Is antibiotic prophylaxis in children with vesicoureteral reflux effective in preventing pyelonephritis and renal scars? A randomized,	Randomized clinical trial<br><br>Mean follow-up: 24 months	100 patients with vesicoureteral reflux (grade II, III, or IV) diagnosed with cystourethrography after a first episode of acute	Patients were randomly assigned to receive antibiotic prophylaxis with sulfamethoxazole/trimethoprim or not for 2 years.	Recurrence of symptomatic or febrile UTI.<br><br>Deterioration in cortical scintigraphy.	There were no differences in the risk for having at least 1 pyelonephritis episode between the intervention and control groups.<br><br>33 (33%) children presented at least 1 pyelonephritis recurrence in the first 2 years	RoB2:<br>-Risk of UTI recurrence: low

controlled trial (Pennesi et al, 2008)		pyelonephritis, in different centers in Italy.<br><br>Exclusion criteria were the presence of previous episodes of pyelonephritis; VUR grade I; VUR grade V, concerned by the high incidence of associated renal dysplasia; or recurrence of acute pyelonephritis before the first DMSA renal scan.			of follow-up, 18 (36%) in the intervention group and 15 (30%) in the control group. The total number of recurrences was 42 and 35, respectively. The risk for having at least 1 pyelonephritis recurrence was slightly higher but not statistically significant in the intervention than in the control group (RR: 1.2; 95% CI: 0.68 –2.11).<br>At the end of the 4-year follow- up, a worse DMSA was detected for 10 patients, all grade IV: 6 worsened without any recurrence.<br><br>The presence of renal scars was the same in children with or without antibiotic prophylaxis (RR: 1.22; 95% CI: 0.75–1.98).	-Risk of new renal scars: low
Clinical significance of primary vesicoureteral reflux and urinary antibiotic prophylaxis after acute pyelonephritis: a multicenter, randomized, controlled study.<br>(Garin et al, 2006)	Randomized clinical trial<br><br>Mean follow-up: 12 months	218 children aged <18 years enrolled in the USA, Chile, and Spain after a first episode of acute pyelonephritis confirmed through a ^{99m}Tc-DMSA scan, with or without low-grade VUR.<br><br>113 children with any grade VUR.<br><br>Exclusion criteria were the presence of grade IV or V VUR, neurogenic bladder, posterior urethral valves, uri- nary diversion, bladder diverticulum, ureterocele, renal failure, and pregnancy.	Patients were randomly assigned to receive either prophylaxis (sulfamethoxazole/trimethoprim 1–2 mg/kg of trimethoprim or nitrofurantoin 1.5 mg/kg once daily) or no prophylaxis for 12 months.	Recurrence of symptomatic or febrile UTI.<br><br>Deterioration in cortical scintigraphy.	No statistically significant differences were found between the subgroups in terms of recurrences and new renal scars.<br><br>Among the subgroup of 113 children with VUR, recurrent UTI occurred in 13/55 (23.6%) patients on prophylaxis and in 13/58 (22.4%) children not on prophylaxis (p 0.9).<br><br>New renal scars were found in 5/55 (9%) patients on prophylaxis and in 2/58 (3.4%) children not on prophylaxis (p 0.2).	RoB2:<br>-Risk of UTI recurrence: some concerns<br>-Risk of new renal scars: some concerns

Figure S6. Risk of bias 2 results for randomized clinical trials investigating the risk of UTI recurrence.

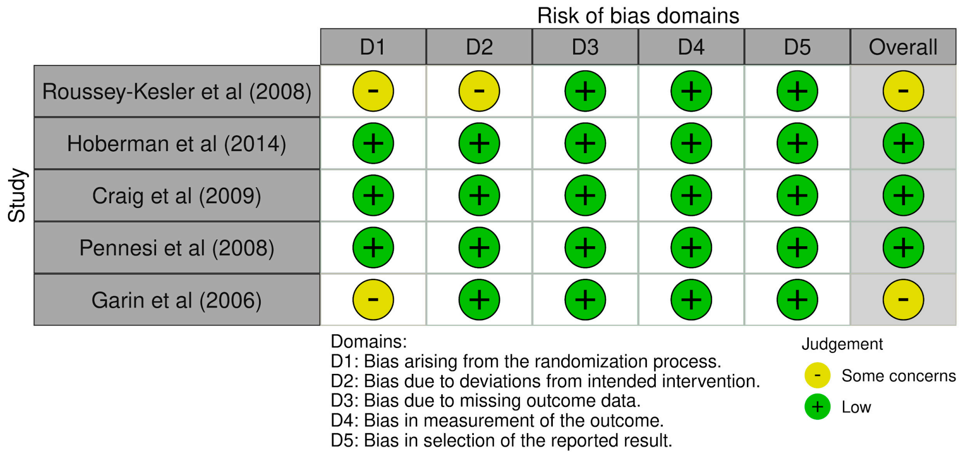

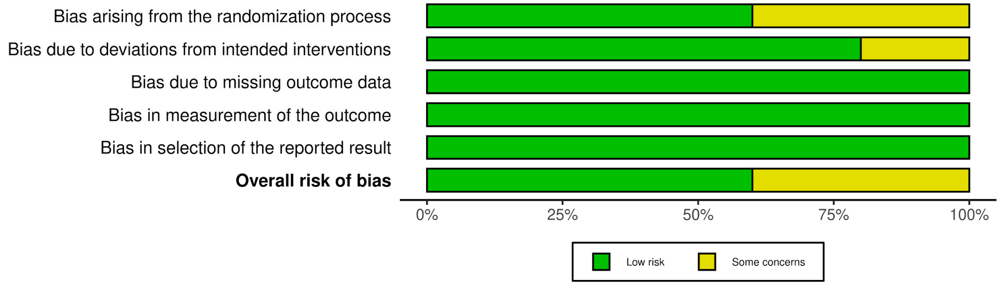

Figure S7. Risk of bias 2 results for randomized clinical trials investigating the risk of new renal scars.

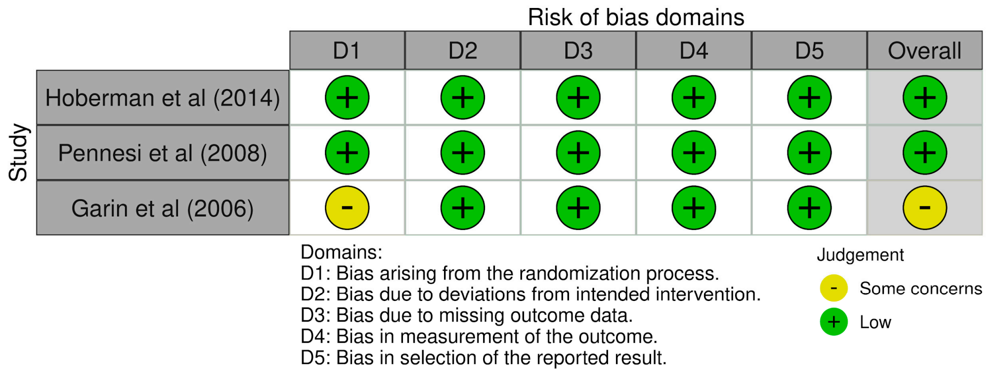

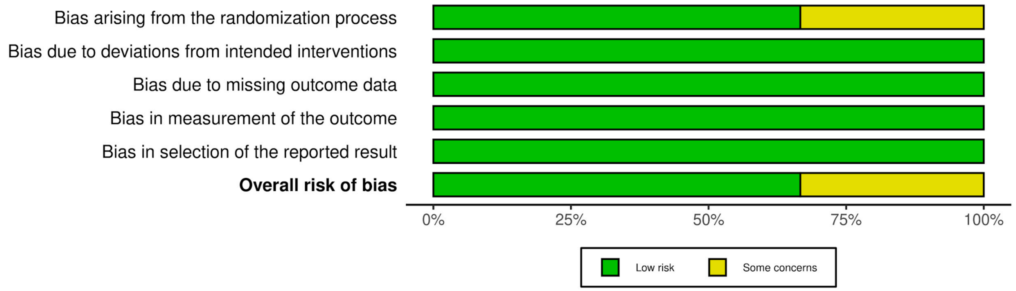

Figure S8. Risk of bias 2 results for randomized clinical trials investigating the risk of new antimicrobial resistances.

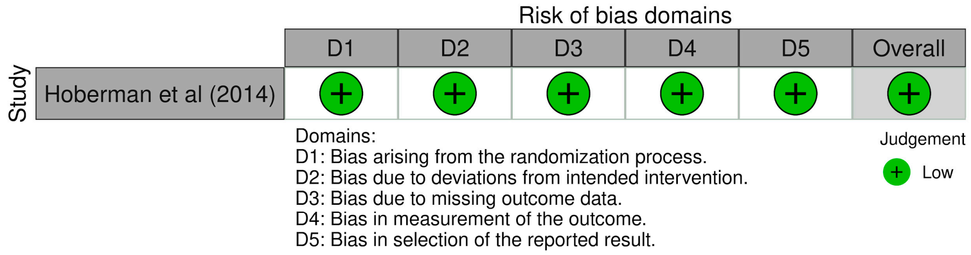

Table S9. Characteristics and results of included systematic reviews.

TITLE	POPULATION, INTERVENTIONS AND OUTCOMES	QUALITY ASSESSMENT (AMSTAR2)	RESULTS	CONCLUSIONS
Antibiotic prophylaxis for children at risk of developing urinary tract infection: a systematic review. (Mori et al, 2009)	The study population included 656 children aged 0–18 years who had had a UTI. A total of 8 trials were included in the review. The trials were stratified by the four population categories:<br>- Children who had symptomatic UTI including those with and without VUR (2 trials).<br>- Children who had symptomatic UTI without VUR (2 trials).<br>- Children who had symptomatic UTI with VUR (3 trials).<br>- Children with VUR (1 trial).<br>Intervention and comparison were antibiotic prophylaxis vs no prophylaxis. The primary outcomes were incidence of new or progressive renal scarring or recurrence of pyelonephritis.	Critically low	- There was no evidence of difference in recurrence of symptomatic UTI in the subgroup of children with VUR between the intervention and control groups (RR 1.27, two trials, 95% CI: 0.81-1.98).<br><br>- There was no evidence of a difference in incidence of new or progressive renal scarring in the subgroup of children with VUR between the intervention and control groups (RR 1.36, two trials, 95% CI: 0.85-2.17).	- There is no evidence of a reduction in the incidence of symptomatic UTI nor in the prevalence of renal scarring.
Long-term antibiotics for the prevention of recurrent urinary tract infection in children: a systematic review and meta-analysis. (Dai et al, 2010)	Eleven clinical trials with 2046 patients were included. All but one of the studies included patients with VUR. The treatment regimen was usually trimethoprim-sulfamethoxazole or nitrofurantoin, but the dose and duration varied among studies.<br><br>To evaluate the effectiveness and safety of long-term prophylactic antibiotics in terms of recurrent UTI and renal scars.	Low	- Antibiotic prophylaxis failed to reduce the risk of recurrent symptomatic UTI (RR 0.94, 95% CI 0.70 to 1.27, $p=0.70$) and repeat urine culture (RR 0.82, 95% CI 0.61 to 1.09, $p=0.17$; test for subgroup differences $p=0.11$, $I2=61.8\%$) in children with VUR.<br><br>- The duration of treatment did not affect prophylactic efficacy (RR for trials with prophylactic time <12 months 1.93, 95% CI 0.63 to 5.92, $p=0.25$ vs RR for trials with prophylactic time ≥12 months 0.81, 95% CI 0.64 to 1.04, $p=0.09$; test for subgroup differences $p=0.14$, $I2=54\%$).<br><br>- The rate of new or deteriorated renal scars was reported by seven studies and showed no significant difference between	- The long-term antibiotic prophylaxis in children was not associated with significant prevention of recurrent symptomatic UTI or new renal damage.<br><br>- Long-term antibiotics promote the development of resistant bacteria.

			antibiotic prophylaxis and placebo/no treatment (RR 0.95, 95% CI 0.51 to 1.78, p=0.87).<br><br>- Among studies included, the incidence of bacterial resistance ranged from 67% to 100% in the prophylaxis groups and from 0% to 39% in the non-prophylaxis groups.	
Technical report—diagnosis and management of an initial uti in febrile infants and young children.<br>(Finnell et al, 2011)	8 randomized clinical trials including children aged <18 years with and without VUR. 6 studies compared antimicrobial prophylaxis with no prophylaxis.<br><br>The grade of VUR among the enrolled children varied from 0 to V.<br><br>The antimicrobial agents used were trimethoprim-sulfamethoxazole, amoxicillin-clavulanic acid or nitrofurantoin.	High	- There was no significant difference in rates of recurrence of pyelonephritis for children 2 to 24 months of age with VUR who received antimicrobial agents and those who did not with RR of 0.78 (95% CI: 0.48 –1.26).<br><br>- When results were analyzed according to VUR grade, there was no significant difference in rates of recurrence of pyelonephritis for children 2 to 24 months of age who received antimicrobial agents and those who did not.<br><br>- There was a statistically significant difference in rates of recurrence of any type of UTI for children with VUR who received antimicrobial agents and those who did not with RR of 0.70 (95% CI: 0.51– 0.96).<br><br>- The antimicrobial resistance patterns of the pathogens isolated during UTI recurrences were assessed in 5 of the RCTs included in the meta-analysis. The proportions of resistant bacteria ranged from 0% to 39%; in the antimicrobial prophylaxis groups, the proportions of resistant bacteria ranged from 53% to 100%.	- The evidence does not support antimicrobial prophylaxis to prevent UTI when VUR is found through VCUG. The only statistically significant effect of antimicrobial prophylaxis was in preventing UTI that included cystitis and asymptomatic bacteriuria.<br><br>- All authors concluded that UTI recurrences with antimicrobial-resistant bacteria were more common in the groups of children assigned randomly to receive antimicrobial prophylaxis.
Antibiotic prophylaxis for urinary tract infection-related renal scarring: a systematic review<br>(Hewitt et al, 2017)	Seven randomized clinical trials (1427 subjects) were included in the meta-analysis that studied the effect on UTI-related renal scarring of antibiotic prophylaxis versus no prophylaxis or placebo.<br><br>Overall population included children aged <18 years with a previous symptomatic or febrile UTI with or without VUR. 99mTc dimercaptosuccinic acid scans were performed at entry into the studies and at the end of follow-up (12-24 months) to detect new scar formation.	High	- Meta-analyses did not show differences in the incidence of new scarring between the prophylaxis and no prophylaxis groups in children with VUR (renal scarring in subgroup with VUR: pooled RR, 0.82; 95% CI 0.51–1.31).	- The lack of influence of prophylaxis on scarring is confirmed by this meta-analysis, which did not demonstrate any benefit, despite the combined studies documenting 1068 patient-years of antibiotic prophylaxis.

Urinary tract infection in children: recurrent infections (Larcombe et al, 2013)	Two systematic reviews and one RCT were included in this updated systematic review.<br><br>- A: The first systematic review included studies if the majority of children (>50%) did not have a renal tract abnormality, or a major neurological, urological, or muscular disease. It included six RCTs (1069 children) comparing prophylactic antibiotics with placebo or no treatment.<br><br>- B: The second systematic review included only children with primary VUR. It involved eight RCTs (1039 children), six comparing antibiotic prophylaxis with no treatment and two versus placebo.<br><br>- C: One RCT, involving 176 children with spina bifida undergoing clean intermittent catheterisation and continuing or discontinuing low-dose prophylactic antibiotics.	Critically low	A<br>- Prophylactic antibiotic did not appear to reduce the risk of symptomatic UTI in children with VUR (2 RCTs, 371 children, RR 0.65, 95% CI 0.39-1.07, P = 0.088) compared to those without VUR (3 RCTs, 491 children, RR 0.56, 95% CI 0.15-2.12, P = 0.40; heterogeneity, I^2 = 62%, P = 0.07).<br><br>- No difference was found between antibiotics and placebo/no treatment (2 RCTs, 914 children, RR 2.31, 95% CI 0.03-170.67, P = 0.70; significant heterogeneity, I^2 = 88%, P = 0.004).<br><br>B<br>- The duration of antibiotic prophylaxis varied from 1 to 3 years. Antibiotic prophylaxis compared to no treatment/placebo did not significantly reduce repeat symptomatic UTI (5 RCTs, 846 children, RR 0.68, 95% CI 0.39-1.17, P = 0.16; significant heterogeneity, I^2 = 57%, P = 0.05) at 1 to 2 years.<br><br>- At 1 to 3 years, antibiotic prophylaxis reduced the risk of the combined outcome of new or progressive renal damage on DMSA scan (3 RCTs, 446 children, RR 0.35, 95% CI 0.15-0.80, P = 0.014). However, there was no significant difference in either of these outcomes alone (new renal abnormality RR 0.27, 95% CI 0.06-1.23, P = 0.089; deterioration of existing abnormality RR 0.68, 95% CI 0.27-1.73, P = 0.42).<br><br>- Risk of microbial resistance to prophylactic drug significantly increased in the prophylactic antibiotic group (4 RCTs, 134 children, RR 2.94 95% CI 1.39-6.25, P = 0.005; significant heterogeneity, I^2 = 60%, P = 0.06).<br><br>- Nitrofurantoin showed a significantly lower risk of resistance than cotrimoxazole (RR 0.54, 95% CI 0.31-0.92). Patients receiving nitrofurantoin were twice as likely to experience side effects (RR 2.18, 95% CI 1.39-3.41).	- Recent, well-conducted RCTs suggest a limited benefit of prophylaxis: 12 to 13 children need to be treated for 1 year to prevent one symptomatic UTI.<br><br>- Prophylactic antibiotics may be more effective than placebo at reducing renal parenchymal scarring in boys aged under 3 years, particularly those with moderate and severe VUR. There is no convincing evidence to support the routine use of prophylactic antibiotics in children after their first episode of pyelonephritis, or with non-dilating VUR.<br><br>- Prophylactic antibiotics increase antimicrobial resistance, and many breakthrough UTIs are caused by resistant organisms.<br><br>- Nitrofurantoin is less liable to cause resistance; unfortunately, this is balanced by more adverse effects and treatment dropouts.<br><br>- There is no evidence comparing different durations of antibiotics.
Uropathogen resistance and antibiotic prophylaxis: a meta-analysis.	6 studies that fulfilled the inclusion criteria with 1290 patients with VUR (any grade).	High	- Prophylaxis reduced the risk of recurrent UTI (18.3% vs 23.1%, RR 0.8, 95% CI 0.6-1.0) but increased the prevalence of multidrug-resistant infections (6.4% vs 1.5%, RR 4.2, 95% CI 2.1-8.3).	- Treating VUR patients with continuous antibiotic prophylaxis decreases the risk of developing a recurrent UTI, but when a recurrent UTI develops, there is an increased

(Selekman et al, 2018)	Interventions included long-term antibiotic prophylaxis versus no prophylaxis or placebo.<br><br>The main outcome was the prevalence of multidrug-resistant recurrent UTIs.<br><br>Of all patients with a first recurrent UTI, 27% had non dilating VUR (grades 1 and 2), and 73% had dilating VUR (grades 3–5), which was not statistically different between the control and prophylaxis groups (P = .62).		- After adjusting for age at study enrollment, sex, VUR grade, and history of previous UTI, individuals receiving prophylaxis had 6.4 times the odds (95% confidence interval: 2.7-15.6) of developing a multidrug-resistant infection.<br><br>- Every 21 reflux patients treated with prophylaxis, 1 UTI was prevented but also 1 multidrug-resistant UTI occurred (NNT=21 for both outcomes).	risk of multidrug resistance. The probability of preventing a recurrent UTI while on prophylaxis is equal to that of developing a resistant UTI while on prophylaxis.
Evaluation and management of recurrent urinary tract infections in children: state of the art.<br>(Awais et al, 2015)	7 randomized clinical trials involving children aged <18 years with VUR and 4 meta-analyses were included. Studies compared antibiotic prophylaxis versus no prophylaxis or placebo.<br><br>Main outcome was the rate of UTI recurrences.	Critically Low	- The meta-analysis including 7 trials showed a significant lower rate of UTIs among children receiving prophylactic antibiotics (1087 children) versus those receiving placebo (total number 1052 children) (high ratio 0.69 [0.52, 0.90]; heterogeneity: Tauz = 0.07; Chiz = 12.03, df = 6, p = 0.06; Iz = 50%; Test for overall effect: Z = 2.67, p = 0.008).<br><br>- Among 4 meta-analyses and systematic reviews, only one showed a significant benefit of antibiotic prophylaxis.<br><br>- No evidence of lower renal scarring due to prophylaxis was found.	- Some sub-groups of children are likely to derive a worthwhile benefit from antibiotic prophylaxis. However, all published randomized trials to date were underpowered to perform sub-group analysis.<br><br>- Watchful waiting of children with low-grade VUR and at low risk of UTI is prudent. Antibiotic prophylaxis should be instituted for children with recurrent UTI or at high risk of renal damage.<br><br>- Evidence from well conducted, large-scale randomized trials suggests that TMP–SMX prophylaxis may reduce the frequency of recurrent UTI modestly, though the incidence of renal scarring remains unchanged. Furthermore, 16 patient-years of TMP–SMX need to be administered to prevent a single UTI.
Long-term antibiotics for preventing recurrent urinary tract infection in children.<br>(Williams et al, 2019)	In this Cochrane updated systematic review, 16 studies (2036 children randomized in clinical trials and 1977 not randomized) were included and .	High	- The estimated reduction in risk of repeat symptomatic UTI for children taking antibiotics was similar and not statistically significant in children with VUR (RR 0.65, 95% CI 0.39 to 1.07) compared to those without VUR (RR 0.56, 95% CI 0.15 to 2.12).	- This review found that long-term antibiotics may reduce the risk of repeat symptomatic infections but the benefits are probably small and must be weighed against the likelihood of antimicrobial resistances.

	Children less than 18 years of age who were at risk of recurrence due to prior infection were included. Studies were included if the majority of participants (> 50%) did not have a predisposing cause such as a renal tract abnormality, including VUR, or a major neurological, urological or muscular disease.<br><br>To assess whether long-term antibiotic prophylaxis was more effective than placebo/no treatment in preventing recurrence of UTI in children, and if so which antibiotic in clinical use was the most effective.		- Data for antibiotic resistance with the analysis estimating the risk of a UTI caused by a bacteria resistant to the prophylactic antibiotic being almost 2.5 times greater in children on antibiotics than for children on placebo or no treatment (RR 2.40, 95% CI 0.62 to 9.26). However, the confidence interval is wide, showing imprecision.	- Long-term, low dose antibiotics to prevent repeat UTI should be reserved for those children at higher risk of repeat infection, such as young infants, and children's clinicians would strongly want to reduce the risk of further infections, such as children with renal abnormalities.
Efficacy of antibiotic prophylaxis in children with vesicoureteral reflux: systematic review and meta-analysis (Wang et al, 2015)	8 RCTs were included in the meta-analysis. Inclusion criteria consisted of age 18 years or younger and history of VUR treated with CAP. Study patients were compared to children with VUR undergoing no treatment or receiving placebo.<br><br>Primary outcome was the odds ratio of having febrile or symptomatic UTI. Secondary outcomes included new renal scarring, antibiotic resistance and any adverse effects related to CAP.	High	- Pooled results demonstrated that CAP significantly reduced the risk of febrile or symptomatic UTI in children with VUR (pooled OR 0.63, 95% CI 0.42–0.96, p = 0.03).<br><br>- CAP was associated with an increased risk of resistant bacteria (pooled OR 8.75, 95% CI 3.52–21.73, p <0.0001).<br><br>- CAP failed to demonstrate any significant impact on new renal scarring or antibiotic related adverse events, because event rates were similar between the groups.	- Pooled RCT results reveal that CAP was associated with a 37% decrease in the odds of febrile or symptomatic urinary tract infection in children with reflux.<br><br>- Rates of new renal scarring were not significantly associated with CAP.<br><br>- Long-term CAP was associated with a higher rate of UTIs due to antibiotic resistant bacteria, there was no increase in associated adverse events reported in the CAP group compared to controls.
Antibiotic prophylaxis for prevention of febrile urinary tract infections in children with vesicoureteral reflux: a meta-analysis of randomized, controlled trials comparing dilated to	6 randomized, controlled trials included a total of 986 children. Dilating and non dilating VUR was observed in 471 (47.7%) and 515 patients (52.3%), respectively.<br><br>When data from the RIVUR trial were included in the meta-analysis, the total number of patients increased to 1593. 751 (47.29%) with dilating VURand 837 (52.71%) with non dilating VUR.	Low	- In children with high-grade VUR the risk of recurrent febrile UTI was 20.84% in those who received antibiotics vs 29.03% in those who did not receive prophylaxis. The relative risk of prophylaxis failure was 0.72 (95% CI 0.56-0.92) and the ARR of UTI recurrence was 8.23%. NNT was 12.15 (p= 0.008).<br><br>- In patients with low-grade VUR the risk of recurrent UTI was 6.44% while on prophylaxis vs 12.94% in those not receiving prophylaxis. The relative risk of prophylaxis failure was 0.51 (95% CI 0.32-0.79) and the ARR of UTI recurrence was 6.51%. NNT was 15.36 (p=0.002).	- After including the RIVUR patients, prophylactic antibiotics seemed helpful to prevent recurrent UTIs in children with VUR of any grade. We also noted that low vs high grade VUR carried a similar relative risk of treatment failure (0.51 vs 0.72), absolute risk reduction of febrile UTI (6.51% vs 8.23%) and NNT (15.36 vs 12.15).

nondilated vesicoureteral reflux. (de Bessa et al, 2015)	Aims were comparing antibiotics to placebo/no treatment or comparing 2 or more antibiotics administered daily for at least 2 months to prevent recurrent UTIs in children with VUR.		- Results were statistically significant only after including data from the RIVUR trial.	
Interventions for primary vesicoureteral reflux (Williams et al, 2019)	Thirty four studies involving 4001 children with VUR were included.<br><br>The aim of this review was to evaluate the available evidence for both benefits and harms of the currently available treatment options for primary VUR: operative, non-operative or no intervention.<br><br>.	High	- Low-dose antibiotic prophylaxis compared to no treatment/placebo may make little or no difference to the risk of repeat symptomatic UTI (9 studies, 1667 children: RR 0.77, 95% CI 0.54 to 1.09; low certainty evidence) and febrile UTI (RR 0.83, 95% CI 0.56 to 1.21; low certainty evidence) at one to two years.<br><br>- At one to three years, antibiotic prophylaxis made little or no difference to the risk of new or progressive renal damage on DMSA scan (8 studies, 1503 children: RR 0.73, 95% CI 0.33 to 1.61; low certainty evidence).<br><br>- Adverse events were reported in four studies with little or no difference between treatment groups (1056 children: RR 0.94, 95% CI 0.81 to 1.08).<br><br>- Antibiotics increased the likelihood of bacterial drug resistance threefold (187 UTIs: RR 2.97, 95% CI 1.54 to 5.74; moderate certainty evidence).	- Compared with no treatment, the use of long-term, low-dose antibiotics may make little or no difference to the number of repeat symptomatic and febrile UTIs and in terms of new renal scarring in children with VUR (low certainty evidence).

Table S10. Characteristics and recommendations of included guidelines

GUIDELINES			RECOMMENDATIONS	GRADING
2019	Updated Italian recommendations for the diagnosis, treatment and follow-up of the first febrile urinary tract infection in young children	Italian Society for pediatric nephrology	a. Antibiotic prophylaxis may be considered in infants and children after treatment of the acute episode until VCUG is performed and with reflux grades IV and V. The analysis of the data regarding recurrent infections does not stand in favour of the use of antibiotic prophylaxis, at least in children with low-grade reflux.	a. Grade C
2010	AUA Guideline: Management and Screening of Primary Vesicoureteral Reflux in Children	AUA	a. Continuous low dose antibiotic prophylaxis (CAP) is recommended for the child less than one year of age with VUR with a history of a febrile UTI. This approach is based on the greater morbidity from recurrent UTI found in this population.<br>b. In the absence of a history of febrile UTI, CAP is recommended for the child less than one year of age with VUR grades III–V who is identified through screening.<br>c. In the absence of a history of febrile UTI, the child less than one year of age with VUR grades I–II who is identified through screening may be offered CAP.	a. Not specified<br>b. Not specified<br>c. Not specified

			d. CAP is recommended for the child with bladder and bowel dysfunction (BBD) and VUR due to the increased risk of UTI while BBD is present and being treated.<br>e. CAP may be considered for the child over one year of age with a history of UTI and VUR in the absence of BBD.<br>f. Observational management without CAP, with prompt initiation of antibiotic therapy for UTI, may be considered for the child over one year of age with VUR in the absence of BBD, recurrent febrile UTIs, or renal cortical abnormalities.	d. Not specified<br>e. Not specified<br>f. Not specified
2015	KHA-CARI guideline: Diagnosis and treatment of urinary tract infection in children	KHA/CARI	a. We suggest that antibiotic prophylaxis be considered in young infants with a severe index UTI and for children with recurrent UTI and/or Grades III–V VUR. (2B)	a. Grade 2B
2011	Urinary tract infection: clinical practice guideline for the diagnosis and management of the initial UTI in febrile infants and children 2 to 24 months	AAP	a. The position of the current subcommittee reflects the new evidence demonstrating antimicrobial prophylaxis not to be effective as presumed previously. Moreover, prompt diagnosis and effective treatment of a febrile UTI recurrence may be of greater importance regardless of whether VUR is present or the child is receiving antimicrobial prophylaxis.	a. Not specified
2021	Update of the EAU/ESPU guidelines on urinary tract infections in children	EAU/ESPU	a. Offer long-term antibacterial prophylaxis in case of high susceptibility to UTI and risk of acquired renal damage and lower urinary tract symptoms<br><br>With increasing resistance rates, one should carefully consider which patients should receive antibacterial prophylaxis, since long-term use has been associated with increased microbial resistance. Its use causes a reduction in the number of recurrent UTIs, but it did not reduce newly acquired renal damage in children with first and second UTI. However, when used in children with anatomic abnormalities of the urinary tracts a reduction in UTI and subsequent renal scarring was shown.	a. Grade 1b; strong

Should antibiotic prophylaxis be used in children with high-grade VUR (III-V)?
P: children aged <18 years with VUR (grade III-V)
I: long-term antibiotic prophylaxis with any molecule
C: no prophylaxis
O1: risk of UTI recurrence
O2: risk of new renal scars
O3: risk of new antimicrobial resistances
O4: risk of drug-related adverse events

Table S11. Characteristics and results of included studies

TITLE	DESIGN	POPULATION	INTERVENTIONS	OUTCOMES	RESULTS	RISK OF BIAS ASSESSMENT (RoB2 or NOS)
The Swedish reflux trial in children: III. Urinary tract infection pattern<br><br>The Swedish reflux trial in children: IV. Renal damage<br><br>(Drzewiecki et al, 2012)	Randomized clinical trial<br><br>Mean follow-up: 24 months	203 children aged <2 years with VUR grade III or IV enrolled in Sweden.<br><br>Study exclusion criteria were previous urogenital surgery, malformation (except dupli- cation), known neurological disease, stone disease, glomerular filtration rate less than 70 ml per minute per 1.73 m^2, split renal function less than 15% or suspected non compliance.	Patients were randomly assigned to antibiotic prophylaxis (n =69), endoscopic injection (n = 66) or surveillance (n =68).	Recurrence of symptomatic or febrile UTI.<br><br>Deterioration in cortical scintigraphy.	10/69 (14.4%) children in the prophylaxis group and 25/68 (36.7%) in the surveillance group experienced febrile UTIs (p 0.003). Differences between groups were especially significant for girls: 19% on prophylaxis, 23% with endoscopic treatment and 57% on surveillance (p = 0.0002).<br><br>New damage was seen in 4 of 68 children (6%) on prophylaxis, 8 of 65 (12%) with endoscopic therapy and 12 of 68 (18%) on surveillance. These differences were not statistically significant (p 0.11).<br><br>Significant differences between groups, in terms of renal damage, were seen for girls: 8 on surveillance, 5 in the endoscopic group and none on prophylaxis (p = 0.0155).	RoB2:<br>-Risk of UTI recurrence: low<br>-Risk of new renal scars: low

Antibiotic prophylaxis and recurrent urinary tract infection in children (Craig et al, 2009)	Randomized clinical trial<br><br>Mean follow-up: 12 months	576 children aged <18 years who had had at least one microbiologically proven urinary tract infection were recruited in 4 centers in Australia.<br><br>129 children with high-grade VUR.<br><br>Children with a known neurologic, skeletal, or urologic predisposing cause or with a known contraindication to trimethoprim–sulfamethoxazole therapy were ineligible.	Patients were randomly assigned to receive either daily trimethoprim–sulfamethoxazole suspension or placebo for 12 months.	Recurrence of symptomatic or febrile UTI.	Among the subgroup of children with high-grade VUR, a breakthrough infection occurred in 9/65 (14%) of children on prophylaxis and in 13/64 (21%) of children not on prophylaxis (p 0.70; HR: 0.65; 95% CI: 0.28-1.52). Differences were not statistically significant.	RoB2:<br>-Risk of UTI recurrence : low

Figure S9. Risk of bias 2 results for randomized clinical trials investigating the risk of UTI recurrence.

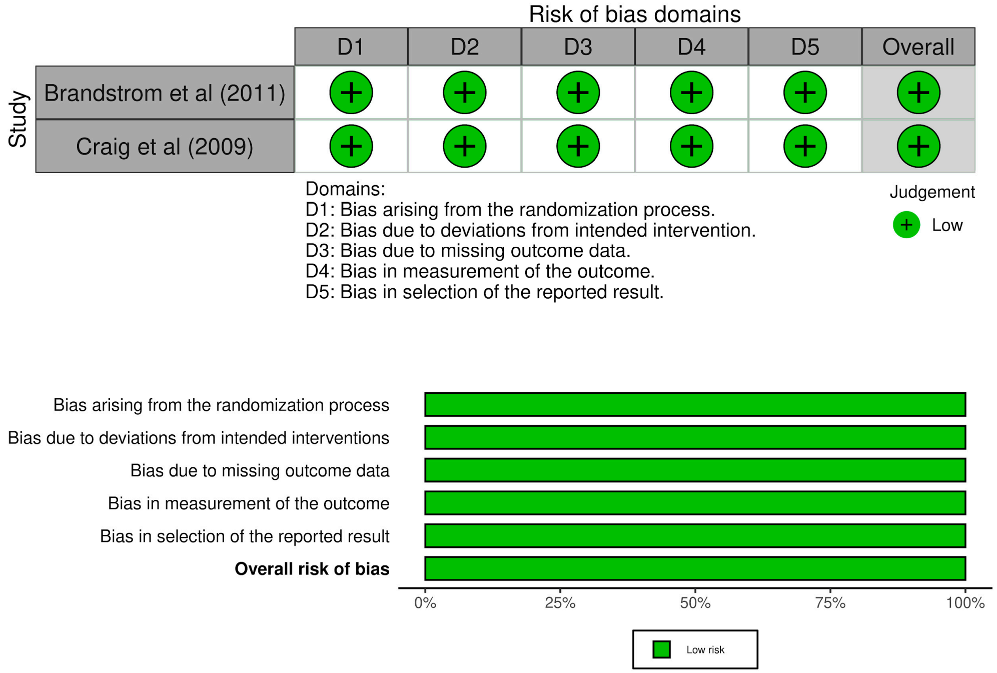

Figure S10. Risk of bias 2 results for randomized clinical trials investigating the risk of new renal scars.

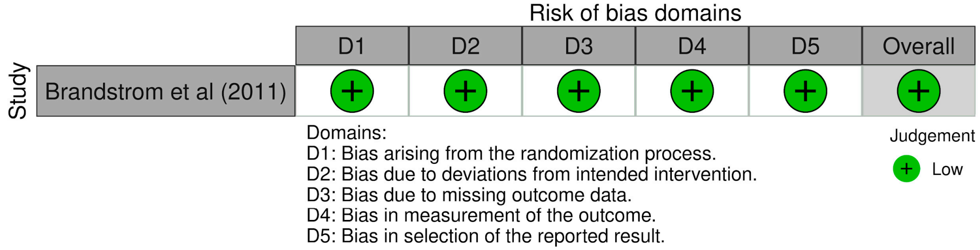

Table S12. Characteristics and results of included systematic reviews

TITLE	POPULATION, INTERVENTIONS AND OUTCOMES	QUALITY ASSESSMENT (AMSTAR2)	RESULTS	CONCLUSIONS
Antibiotic prophylaxis for prevention of febrile urinary tract infections in children with vesicoureteral reflux: a meta-analysis of randomized, controlled trials comparing dilated to nondilated vesicoureteral reflux. (de Bessa et al, 2015)	6 randomized, controlled trials included a total of 986 children. Dilating and non-dilating VUR was observed in 471 (47.7%) and 515 patients (52.3%), respectively.<br>When data from the RIVUR trial were included in the meta-analysis, the total number of patients increased to 1593. 751 (47.29%) with dilating VUR and 837 (52.71%) with non-dilating VUR.<br>Aims were comparing antibiotics to placebo/no treatment or comparing 2 or more antibiotics administered daily for at least 2 months to prevent recurrent UTIs in children with VUR.	Low	- In children with high-grade VUR the risk of recurrent febrile UTI was 20.84% in those who received antibiotics vs 29.03% in those who did not receive prophylaxis. The relative risk of prophylaxis failure was 0.72 (95% CI 0.56-0.92) and the ARR (absolute risk reduction) of UTI recurrence was 8.23%. NNT was 12.15 (p= 0.008).<br>- Results were statistically significant only after including data from the RIVUR trial.	- After including the RIVUR patients, prophylactic antibiotics seemed helpful to prevent recurrent UTIs in children with VUR of any grade. We also noted that low vs high grade VUR carried a similar relative risk of treatment failure (0.51 vs 0.72), absolute risk reduction of febrile UTI (6.51% vs 8.23%) and NNT (15.36 vs 12.15).

Table S13. Characteristics and recommendations of included guidelines

GUIDELINES			RECOMMENDATIONS	GRADING
2019	Updated Italian recommendations for the diagnosis, treatment and follow-up of the first febrile urinary tract infection in young children	Italian Society for pediatric nephrology	a. Antibiotic prophylaxis may be considered in infants and children after treatment of the acute episode until VCUG is performed and with reflux grades IV and V. The analysis of the data regarding recurrent infections does not stand in favour of the use of antibiotic prophylaxis, at least in children with low-grade reflux.	a. Grade C
2021	Asian guidelines for urinary tract infection in children	Yang SS et al	a. Antibiotic prophylaxis to prevent recurrent febrile UTI is indicated in children with moderate to high grade (III–V) VUR.	a. Grade A
2021	Update of the EAU/ESPU guidelines on urinary tract infections in children	EAU/ESPU	a. Offer long-term antibacterial prophylaxis in case of high susceptibility to UTI and risk of acquired renal damage and lower urinary tract symptoms<br><br>With increasing resistance rates, one should carefully consider which patients should receive antibacterial prophylaxis, since long-term use has been associated with increased microbial resistance. Its use causes a reduction in the number of recurrent UTIs, but it did not reduce newly acquired renal damage in children with first and second UTI. However, when used in children with anatomic abnormalities of the urinary tracts a reduction in UTI and subsequent renal scarring was shown.	a. Grade 1b; strong
2015	KHA-CARI guideline: Diagnosis and treatment of urinary tract infection in children	KHA/CARI	a. We suggest that antibiotic prophylaxis be considered in young infants with a severe index UTI and for children with recurrent UTI and/or Grades III–V VUR.	a. Grade 2B

Should antibiotic prophylaxis be used in children with isolated hydronephrosis?
P: children aged <18 years with isolated hydronephrosis
I: long-term antibiotic prophylaxis with any molecule
C: no prophylaxis
O1: risk of UTI recurrence
O2: risk of new renal scars
O3: risk of new antimicrobial resistances
O4: risk of drug-related adverse events

Table S14. Characteristics and results of included studies

TITLE	DESIGN	POPULATION	INTERVENTIONS	OUTCOMES	RESULTS	RISK OF BIAS ASSESSMENT (RoB2 or NOS)
The association between continuous antibiotic prophylaxis and UTI from birth until initial postnatal imaging evaluation among newborns with antenatal hydronephrosis. (Varda et al, 2018)	Observational retrospective cohort study<br><br>Mean follow-up: 1 month	494 included infants aged <3 months undergoing renal ultrasound for an indication of 'hydronephrosis' between 2012 and 2014, in a single center in the USA.<br><br>Exclusion criteria: severe congenital genitourinary anomalies; no clinical follow-up; a history of prenatal intervention or postnatal surgery prior to their initial renal ultrasound (RUS).	Any infant starting antibiotic prophylaxis within 7 days of life and continuing it through initial imaging evaluation was allocated to the CAP cohort. These infants were compared to the remaining cohort who did not receive CAP prior to imaging (or first UTI).<br><br>.	Risk factors for symptomatic or febrile UTI.	UTI prior to initial imaging occurred in seven infants (1.4%): six (1.8%) without CAP versus one (0.6%) with CAP (P=0.44).<br><br>Timing of postnatal imaging evaluation may be a factor in neonatal UTI risk: infants with UTI were significantly older at the time of initial imaging (median 9.4 weeks) compared to those without a UTI. So if imaging is performed early in the neonatal period, starting CAP from birth may not be necessary.	NOS 7
Continuous antibiotic prophylaxis reduces the risk of febrile UTI in children with asymptomatic antenatal hydronephrosis with	Observational retrospective cohort study<br><br>Mean follow-up: 24 months	405 children aged <6 months with antenatal or congenital hydronephrosis from 2001 to 2011 in different centers in the USA.	The first group was those children who had been maintained on continuous antibiotic prophylaxis (CAP) for a period no shorter than 3	Risk factors for symptomatic or febrile UTI.	The global incidence of febrile UTI during the follow-up period was 22.2%. The rates of febrile UTI between the YCAP and NCAP groups were significantly different (YCAP 7.9% vs NCAP 18.7%, p = 0.021).	NOS: 7

either ureteral dilation, high-grade vesicoureteral reflux, or ureterovesical junction obstruction. (Herz et al, 2014)		Children whose primary referral was for UTI, those with incomplete medical records, and those with <2 years of follow-up data were excluded.	months (YCAP, n 278, 68.6%)), and the second group were those who were not maintained on CAP (NCAP, n 127, 31.4%).		Multivariate analysis found that only ureteral obstruction at the ureterovesical junction, ureteral dilation >11 mm, and high-grade VUR were independent risk factors for febrile UTI in children with congenital hydronephrosis. Therefore CAP may have a significant role in reducing the risk of febrile UTI in children with those risk factors, but otherwise seems unnecessary.	

Table S15. Characteristics and results of included systematic reviews.

TITLE	POPULATION, INTERVENTIONS AND OUTCOMES	QUALITY ASSESSMENT (AMSTAR2)	RESULTS	CONCLUSIONS
Role of antibiotic prophylaxis in antenatal hydronephrosis: a systematic review from the European Association of Urology/European Society for Paediatric Urology Guidelines Panel. (Silay et al, 2017)	Children (< 18 years old) with hydronephrosis (all grades) diagnosed prenatally and confirmed postnatally or diagnosed postnatally within the first year of life. The presence/absence of vesico-ureteric reflux was not an exclusion criterion. Children with solitary kidney, posterior urethral valves, bladder exstrophy, and neurological abnormality were excluded.<br><br>The experimental intervention was administration of antibiotic prophylaxis in asymptomatic patients only. The control intervention was observation or no treatment.<br><br>The primary benefit outcome was the reduction in UTI recurrence. The secondary outcomes were reduction in UTI measured after 2 years of life and function of kidney, defined in the following ways:	Moderate	- Most of the studies had low-to-moderate quality of evidence and with high risk of bias.<br><br>The results of the forest plot tables demonstrate five important findings. First, it is not possible to establish whether prophylaxis was superior to no prophylaxis in terms of decreasing UTI. Second, uncircumcised infants, high-grade hydronephrosis, and hydro-ureteronephrosis may be at higher risk of developing UTI. Finally, there was no significant difference in UTI risk between males and females. No conclusion could be drawn for the impact of VUR and no VUR and comparison of the different degrees of VUR because of lack of data in the available literature.<br><br>- The best type of the antibiotic regimen and the adverse effects of the antibiotics could not be assessed either.	- It remains unclear whether CAP is superior to observation in decreasing UTIs. No conclusion could be drawn for drug-related adverse events and kidney function because of lack of data.<br><br>- Children who were not circumcised, with ureteral dilatation, and high-grade hydronephrosis may be more likely to develop UTI, and CAP may be warranted for these subgroups of patients.

	(1) renography; (2) renal scarring; (3) anatomical or morphological changes.			
Antibiotic prophylaxis for prevention of urinary tract infections in the first year of life in children with vesicoureteral reflux diagnosed in the workup of antenatal hydronephrosis: a systematic review. (Leigh et al, 2019)	18 studies selected, giving a total population of 829 children.<br>The primary outcome was to compare the rate of UTI among infants with prenatal hydronephrosis receiving CAP versus those who were not, in the first year of life.<br>Secondary analyses included rates of UTI stratified by gender, VUR grade, and circumcision status in males.	Critically Low	- 15.4% of children receiving CAP from birth developed at least one breakthrough UTI.<br>- In a combined population of 96 patients from two studies, 94.8% were on CAP. Rate of UTI while on CAP was 12.6% as compared to 33.4% in patients not on CAP.	- The limited data available showed no conclusive benefit of CAP, primarily due to lack of a strong comparator cohort.

Should antibiotic prophylaxis be used in children with infravesical obstructions (urethral valves)?
P: children aged <18 years with infravesical obstruction (urethral valves)
I: long-term antibiotic prophylaxis with any molecule
C: no prophylaxis
O1: risk of UTI recurrence
O2: risk of new renal scars
O3: risk of new antimicrobial resistances
O4: risk of drug-related adverse events

No studies nor systematic reviews were included.

Table S16. Characteristics and recommendations of included guidelines

GUIDELINES			RECOMMENDATIONS	GRADING
2021	Update of the EAU/ESPU guidelines on urinary tract infections in children	EAU/ESPU	a. Offer long-term antibacterial prophylaxis in case of high susceptibility to UTI and risk of acquired renal damage and lower urinary tract symptoms<br><br>With increasing resistance rates, one should carefully consider which patients should receive antibacterial prophylaxis, since long-term use has been associated with increased microbial resistance. Its use causes a reduction in the number of recurrent UTIs, but it did not reduce newly acquired renal damage in children with first and second UTI. However, when used in children with anatomic abnormalities of the urinary tracts a reduction in UTI and subsequent renal scarring was shown.	a. Grade 1b; strong

Should antibiotic prophylaxis be used in children with hydroureteronephrosis/ureteral dilation (primary obstructive megaureter)?
P: children aged <18 years with hydroureteronephrosis/ureteral dilation (primary obstructive megaureter)
I: long-term antibiotic prophylaxis with any molecule
C: no prophylaxis
O1: risk of UTI recurrence
O2: risk of new renal scars
O3: risk of new antimicrobial resistances
O4: risk of drug-related adverse events

Table S17. Characteristics and results of included studies

TITLE	DESIGN	POPULATION	INTERVENTIONS	OUTCOMES	RESULTS	RISK OF BIAS ASSESSMENT (RoB2 or NOS)
Complications and long-term outcome of primary obstructive megaureter in childhood (Gimpel et al, 2010)	Observational retrospective cohort study<br><br>Mean follow-up: 47 months	44 patients with primary obstructive megaureter consecutively diagnosed from 1994 to 2006 in a single center in Germany.<br><br>Authors excluded patients with secondary megaureters (e.g. urethral valves, Prune-Belly syndrome, megaureters with both obstruction and reflux and ureteroceles) as well as congenital megaureters that were not obstructive (i.e. only type A on diuresis renogram). Patients with duplicated kidneys or duplicated ureters were also excluded. Only patients	Out of 44 children studied during the first year of life, 30 received antibiotic prophylaxis.<br><br>About two-thirds of children with prophylaxis received a second-generation cephalosporin (cefaclor, 63%), while 32% received trimethoprim and 5% nitrofurantoin.	To evaluate the long-term prognosis of children with POM managed with a primarily conservative approach. Regression of a POM defined by a normal diuresis renogram (type A) on follow-up or disappearance of hydronephrosis and megaureter on ultrasound in the cases without follow-up renogram.<br><br>Adverse clinical outcomes included UTI, reduced global renal function, renal atrophy on ultrasound, reduced partial function on MAG3 renogram (<45%), loss of	7 UTIs occurred in 30 children during 199 patient-months with prophylaxis compared to 19 UTIs in 14 children during 244 patient-months without prophylaxis, corresponding to a reduction of UTI incidence by 55% attributable to prophylaxis (0.94 vs. 0.42 UTIs per year, $p<0.05$).<br><br>Prophylaxis appeared particularly effective in the<br>first 6 months of life, where an 83% reduction of UTI rate was found.	NOS 7

		who were followed-up for at least 1 year and who had at least one ultrasound and one renal isotope scan were analyzed.		the kidney by nephrectomy, or renal hypertension.<br>.		
Risk of urinary tract infection in patients with hydroureter: An analysis from the Society of Fetal Urology Prenatal Hydronephrosis Registry (Holzman et al, 2021)	Observational prospective cohort study<br><br>Mean follow-up: 2.2 years	237 patients with antenatal hydroureteronephrosis enrolled in 7 centers in the USA from 2008 to 2020.<br><br>Children with the following were excluded: ureterocele, bladder diverticulum, posterior urethral valves, urethral atresia, neurogenic bladder, prune belly syndrome, nephrolithiasis, horseshoe kidney, multicystic dysplastic kidney, solitary kidney, suspected ureteropelvic junction obstruction and/or history of pyeloplasty.	Clinical variables collected included imaging results, continuous antibiotic prophylaxis use, and the development of UTI based on urinalysis, urine culture and antibiotic treatment.	Risk factors for UTI.	CAP was significantly protective against UTI (HR Z 0.50 (95% CI: 0.28-0.87), p = 0.01).<br><br>Patients with ureters 7 mm or greater had nearly three times the risk of UTI adjusting for sex, circumcision status, antibiotic prophylaxis and hydronephrosis grade (HR Z 2.7, 95% CI: 1.1-6.5, p= 0.03).<br><br>Among patients who underwent VCUG and did not have vesicoureteral reflux, ureteral dilation 7 mm or greater corresponded with higher UTI risk compared to ureteral diameter less than 7 mm on multivariable analysis (HR Z 4.6, 95% CI: 1.1-19.5, p =0.04).	NOS: 7
The fate of primary non-refluxing megaureter: a prospective outcome analysis of the rate of urinary tract infections, surgical indications and time to resolution (Braga et al, 2016)	Observational retrospective cohort study<br><br>Mean follow-up: 47 months	80 consecutive patients aged <2 years with primary megaureter diagnosed between 2008 and 2015 in Canada.<br><br>Patients with simple duplex kidneys, ectopic ureter, ureterocele, posterior urethral valves, multicystic dysplastic kidney, horseshoe kidney, neurogenic bladder and	Potential risk factors for UTI were investigated and included gender, circumcision status, hydronephrosis grade (low vs high), continuous antibiotic prophylaxis use, ureteral dilatation (7 to 11 vs greater than 11 mm), and presence of ureteral tortuosity.	Risk factors for UTI.	Overall continuous antibiotic prophylaxis was prescribed to 34 patients (43%) and febrile urinary tract developed infection in 27 (34%) at a mean age of 5.8 months (median 3, range 1 to 24).<br><br>Cox regression identified uncircumcised male gender (HR 3.4, 95% CI 1.1-0.7, p = 0.04) and lack of continuous antibiotic prophylaxis (HR 4.1, 95% CI 1.3-12.7, p =0.01) as independent risk factors for febrile urinary tract infection.	NOS: 6

		prune belly syndrome as well as those with isolated renal pelvic dilatation (or ureteropelvic junction obstruction-like) and VUR were excluded.				

Table S18. Characteristics and results of included systematic reviews.

TITLE	POPULATION, INTERVENTIONS AND OUTCOMES	QUALITY ASSESSMENT (AMSTAR2)	RESULTS	CONCLUSIONS
Febrile urinary tract infections in children with primary non-refluxing megaureter: a systematic review and meta-analysis (Rohner et al, 2020)	16 studies (749 patients) including children less than 18 years of age with primary megaureter (defined as dilatation of the ureter >7 mm down to an abnormal uretero-vesical junction with or without concomitant pelvicalyceal dilatation). For all patients VUR was excluded.<br><br>Exclusion criteria included: secondary megaureter, associated other urinary tract anomalies or comorbidities which might affect the rate of fUTI, previous urinary tract surgery, and receiving antibiotic prophylaxis for any other reason.<br><br>The primary outcome was the prevalence of UTI. The secondary outcomes were quality of life and adverse effects associated with the use of antibiotic prophylaxis.	Moderate	- The overall pooled prevalence of UTI in patients with primary non-refluxing megaureter was 14.35 % (95 % CI: 8.8-22.6).<br><br>- The prevalence of UTI in patients on prophylaxis was 10.3 % (95 %CI: 4.8-20.8; I2=74%; 9 studies; 275 patients) compared to 33.0% without prophylaxis (95 %CI: 16.5-55.1; I2 =79 %; 4 studies; 134 patients).<br><br>- The calculated number needed to treat to prevent one single febrile urinary tract infection over the course of 1–2 years would be 4.3.	- The use of continuous antibiotic prophylaxis for children with primary megaureter selected for primary non-surgical treatment should be taken into consideration, at least in patients with urinary outflow impairment, higher grade of ureteral dilatation, and for children in the first months of life.

Should antibiotic prophylaxis be used in children with neurogenic bladder?
P: children aged <18 years with neurogenic bladder
I: long-term antibiotic prophylaxis with any molecule
C: no prophylaxis
O1: risk of UTI recurrence
O2: risk of new renal scars
O3: risk of new antimicrobial resistances
O4: risk of drug-related adverse events

Table S19. Characteristics and results of included studies

TITLE	DESIGN	POPULATION	INTERVENTIONS	OUTCOMES	RESULTS	RISK OF BIAS ASSESSMENT (RoB2 or NOS)
The impact of constant antibiotic prophylaxis in children affected by spinal dysraphism performing clean intermittent catheterization: a 2-year monocentric retrospective analysis (Mariani et al, 2021)	Observational retrospective cohort study<br><br>Mean follow-up: 24 months	121 children with spina bifida enrolled in a single center in Italy and treated with clean intermittent catheterization. 90 patients (74.4%) were affected by myelomeningocele and 31 (25.6%) had other forms of SD such as dermal sinus, lipomeningomyelocele, and tethered cord.	Antibiotic prophylaxis and type of prophylaxis (oral/in the bladder), age of starting prophylaxis and its duration, number of CIC/day, and presence and grade VUR were investigated as possible risk factors.<br><br>During the study period, 85 (70%) patients received antibiotic prophylaxis (ABP group) and 36 (30%) did not (NABP group)	Risk factors for UTI.<br>.	66 of 121 patients (54%) presented ≥1 episode of UTIs and 55 (46%) none.<br><br>No statistically significant difference in terms of UTI rate was observed between the ABP and NABP groups (p=0.17).<br><br>Compliance to the prophylaxis was further evaluated: 71 patients (59%) took antibiotic prophylaxis properly (CABP group) and 50 (41%) did not (NCABP group). A statistically significant difference was observed with a 2.2 times higher risk of development at least one episode of UTIs in NCABP group.	NOS 6

Nitrofurantoin prophylaxis for bacteriuria and urinary tract infection in children with neurogenic bladder on intermittent catheterization (Schlager et al, 1998)	Randomized crossover clinical trial.<br><br>Mean follow-up: 11 months	15 children aged <18 years with neurogenic bladder due to myelomeningocele and undergoing clean intermittent catheterization were enrolled in the USA.<br><br>Patients had a normal renal ultrasound and voiding cystourethrogram. All children continued to receive medical care from their primary physician; no therapies were withheld or altered	Patients were randomly assigned to receive nitrofurantoin prophylaxis or placebo for 5 months (330 total patient weeks), then 4 weeks of no drug (washout) for all patients, and then 5 months (330 total patient weeks) of the alternate study drug.<br><br>A "washout" period was provided to prevent a carryover effect of nitrofurantoin into the placebo period.	Risk of asymptomatic bacteriuria and symptomatic UTI.	203/274 (74%) samples from the placebo periods were positive compared with 165/252 (65%) samples from the nitrofurantoin periods.<br><br>Escherichia coli, the most common pathogen isolated during placebo, was replaced by resistant Klebsiella spp. and Pseudomonas spp. during nitrofurantoin. The carriage of these resistant organisms tripled during nitrofurantoin prophylaxis.<br><br>Rate of symptomatic infection dropped in half during prophylaxis. Despite an increased prevalence of resistant pathogens observed for asymptomatic bacteriuria during the nitrofurantoin prophylaxis, an increase in symptomatic UTIs caused by these resistant organisms did not occur.	RoB2 for crossover trials:<br>-Risk of UTI recurrence: some concerns<br>-Risk of antimicrobial resistances: some concerns
The influence of antibiotic prophylaxis on bacterial resistance in urinary tract infections in children with spina bifida.<br>(Zegers et al, 2017)	Randomized clinical trial.<br><br>Mean follow-up: 18 months	176 pediatric patients with spina bifida (myelomeningocele) enrolled in 2 centers in the Netherlands and Belgium. All children underwent clean intermittent catheterization and received antibiotic prophylaxis before enrollment.	Patients were randomly assigned (1:1) to continue or stop antibiotic prophylaxis.<br><br>The prophylactic regimens were allowed to differ between patients according to antimicrobial susceptibility in pre-study cultures.	Risk factors for antimicrobial resistances.	Microbial resistance against any antibiotic was present in 65.2% of UTIs, and significantly more prevalent in urine cultures taken in children on prophylaxis (72.2%) than in children without prophylaxis (53.3%).<br><br>Stopping prophylaxis decreased the percentage of resistance in E.coli UTIs against amoxicillin and piperacillin from 73.8% and 73.5% to 56.3% and 59.5%, respectively. Resistance against amoxicillin/clavulanic acid (29.7%) and piperacillin/tazobactam (7.8%) was less common and, when discontinuing AP, decreased to 22.7% and 5.5%, respectively.	RoB2:<br>-Risk of antimicrobial resistances: some concerns
Are prophylactic antibiotics necessary with clean intermittent	Randomized clinical trial.	53 children with spina bifida enrolled in a single center in Hong Kong and	The randomization allocated all patients into one of two groups: (A)	Risk of UTI recurrence.	The incidence of urinary tract infections was significantly increased in the group who continued to use antibiotics (n = 20)	RoB2:<br>-Risk of UTI

catheterization? A randomized controlled trial (Clarke et al, 2004)	Mean follow-up: 4 months	undergoing clean intermittent catheterization	continuing antibiotics or (B) discontinuing antibiotics.		when compared with the group who discontinued prophylaxis (n = 3).<br><br>There were 31 patients in group A that continued prophylaxis, of whom 11(36%) remained infection-free and 20 (64%) developed at least one UTI. 14/20 (70%) patients that developed a UTI in group A were not self-catheterizing. In the group who discontinued antibiotics, 19 (86%) remained infection-free and 3 (14%) developed at least one UTI. Patients who experienced UTIs in group B were all catheterized by either a parent or a caregiver.<br>The difference in UTI rates between the 2 groups proved significant ($P < .0001$).	recurrence : some concerns
Antibiotic prophylaxis for urinary tract infections in children with spina bifida on intermittent catheterization (Zegers, 2011)	Randomized clinical trial.<br><br>Mean follow-up: 18 months	176 pediatric patients with spina bifida (myelomeningocele) enrolled in 2 centers in the Netherlands and Belgium. All children underwent clean intermittent catheterization and received antibiotic prophylaxis before enrollment.	Patients were randomly assigned (1:1) to continue or stop antibiotic prophylaxis.<br><br>The prophylactic regimens were allowed to differ between patients according to antimicrobial susceptibility in pre-study cultures.	Risk of UTI recurrence and asymptomatic bacteriuria.	Discontinuation of low dose chemoprophylaxis resulted in higher rates of asymptomatic significant bacteriuria (incidence rate ratio 1.23, 95% CI 1.08–1.40, p =0.002) and urinary tract infection (IRR 1.44, 95% CI 1.13–1.83, p 0.003).<br><br>For UTI the number needed to harm was 2.2, that is if 2 patients discontinued low dose chemoprophylaxis for a year, 1 extra UTI would result.<br><br>Of 88 patients allocated to discontinuation of low dose chemoprophylaxis 38 (43%) switched back to chemoprophylaxis.	RoB2:<br>-Risk of UTI recurrence : high

Figure S11. Risk of bias 2 results for randomized clinical trials investigating the risk of UTI recurrence.

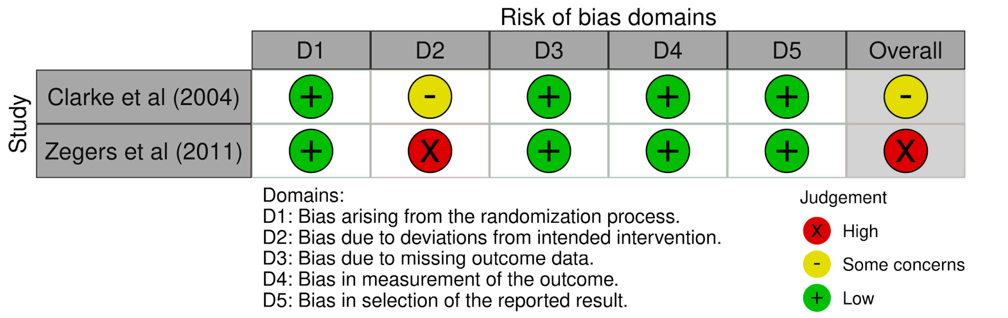

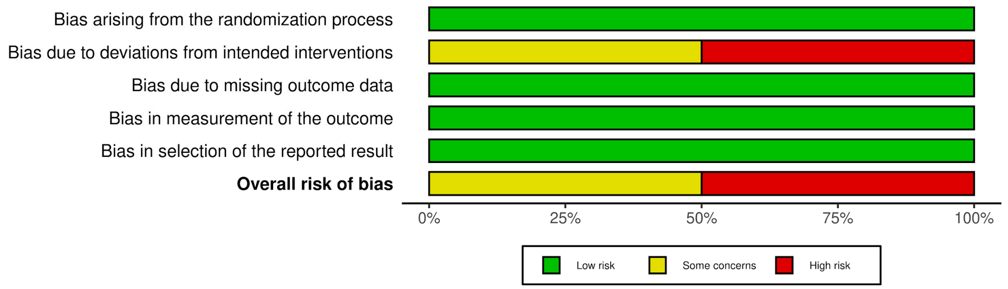

Figure S12. Risk of bias 2 results for randomized clinical trials investigating the risk of new antimicrobial resistances.

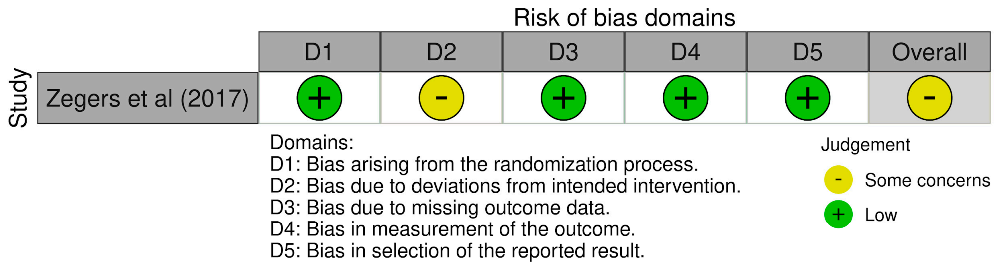

Table S20. Characteristics and results of included systematic reviews.

TITLE	POPULATION, INTERVENTIONS AND OUTCOMES	QUALITY ASSESSMENT (AMSTAR2)	RESULTS	CONCLUSIONS
Urinary tract infection in children: recurrent infections (Larcombe et al, 2013)	Two systematic reviews and one RCT were included in this updated systematic review.<br>- A: The first systematic review included studies if the majority of children (>50%) did not have a renal tract abnormality, or a major neurological, urological, or muscular disease. It included six RCTs (1069 children) comparing prophylactic antibiotics with placebo or no treatment.<br>- B: The second systematic review included only children with primary VUR. It involved eight RCTs (1039 children), six comparing antibiotic prophylaxis with no treatment and two versus placebo.<br>- C: One RCT, involving 176 children with spina bifida undergoing clean intermittent catheterisation and continuing or discontinuing low-dose prophylactic antibiotics.	Critically low	C<br>- Discontinuation of prophylaxis led to higher rates of asymptomatic bacteriuria (RR 1.25, 95% CI 1.08-1.40, P = 0.002) and afebrile UTIs (RR 1.44, 95% CI 1.13-1.83, P = 0.003), but there was no difference in the number of febrile UTIs (RR 2.0, 95% CI 0.38-10.6, P = 0.42).	- Recent, well-conducted RCTs suggest a limited benefit of prophylaxis in patients with spina bifida, often limited to reduction of asymptomatic bacteriuria.
Evaluating the benefits of antimicrobial prophylaxis to prevent urinary tract infections in children: a systematic review (Le Saux et al, 2001)	6 trials included children aged <18 years with at least one previous UTI. 3 trials dealt with children who had normal urinary tracts, and 3 included children with VUR or neurogenic bladder.<br><br>Interventions were antibiotic prophylaxis compared to no prophylaxis or placebo. The outcome of interest was the recurrence of UTI.	Low	- The recurrence rates for patients with neurogenic bladders in 2 trials were 2.9 and 17.1 per 10 patient-years for the treatment groups and 1.5 and 33.0 for the control groups.	- Because the magnitude of benefit of prophylactic antimicrobials may be small and a potential for harboring resistant bacteria may exist, they should be used only after careful consideration and only after attempts have been made to correct conditions that predispose to urinary stasis (e.g., voiding dysfunction or constipation).

Table S21. Characteristics and recommendations of included guidelines.

GUIDELINES			RECOMMENDATIONS	GRADING
2021	Update of the EAU/ESPU guidelines on urinary tract infections in children	EAU/ESPU	a. Offer long-term antibacterial prophylaxis in case of high susceptibility to UTI and risk of acquired renal damage and lower urinary tract symptoms.<br><br>Patients with incomplete emptying of the bladder appropriately performing CIC, but still suffering from recurrent UTIs the intravesical application of gentamicin has been proven effective	a. Grade 1b; strong

Which antibiotic should be preferred for long-term prophylaxis of UTI in children?

P: children aged <18 years at risk of UTI

I1: oral cephalosporins

I2: trimethoprim-sulfamethoxazole

I3: nitrofurantoin

C: amoxicillin-clavulanic acid

O1: risk of UTI recurrence

O2: risk of new renal scars

O3: risk of new antimicrobial resistances

O4: risk of drug-related adverse events

Table S22. Characteristics and results of included studies

TITLE	DESIGN	POPULATION	INTERVENTIONS	OUTCOMES	RESULTS	RISK OF BIAS ASSESSMENT (RoB2 or NOS)
Antibiotic resistance patterns of community acquired urinary tract infections in children	Observational retrospective cohort study	420 patients aged <15 years with VUR (any grade) and receiving antibiotic	Patients received co-trimoxazole, cephalexin, or cefaclor prophylaxis or prophylaxis with a	Risk factors for new antimicrobial resistances.	324 patients underwent antibiotic prophylaxis (109 with co-trimoxazole, 100 with cephalexin, 44 with cefaclor, and 71 with alternative monotherapy) in one	NOS 7

with vesicoureteral reflux receiving prophylactic antibiotic therapy.<br>(Cheng et al, 2007)	Mean follow-up: 5 years	prophylaxis for >3 months in 2 centers in Taiwan.<br><br>All children received Authors focused on children who developed break- through UTIs during the prophylaxis period. All patients diagnosed as having breakthrough UTIs were admitted with fever and positive urine culture results.<br><br>Authors excluded the isolates if the patients had received outpatient antibiotic treatment (other than prophylaxis) or if the isolates were obtained just after a previous course of treatment for any bacterial infection.	sequence of different antibiotics (alternative monotherapy).<br><br>Demographic data, degree of vesicoureteral reflux, prophylactic antibiotics prescribed, and antibiotic sensitivity results of first urinary tract infections and breakthrough urinary tract infections were recorded.		hospital and 96 children underwent co-trimoxazole prophylaxis in the other hospital.<br><br>Breakthrough urinary tract infections occurred in patients from both hospitals (20.4% and 25%, respectively).<br><br>Recurrent UTI occurred in 66/205 receiving co-trimoxazole, 22/100 cephalexin, 12/44 cefaclor, 22/71 alternative. New resistance to the prophylactic antibiotic occurred in 4/66 co-trimoxazole, 12/21 cephalexin, 5/12 cefaclor.<br><br>Children receiving cephalosporin prophylaxis were more likely to have an extended-spectrum lactamase-producing organism for breakthrough urinary tract infections, compared with children with co-trimoxazole prophylaxis. Antimicrobial susceptibilities to almost all antibiotics decreased with cephalosporin prophylaxis when recurrent urinary tract infections developed. The extent of decreased susceptibilities was also severe for prophylaxis with a sequence of different antibiotics. However, antimicrobial susceptibilities decreased minimally in cotrimoxazole prophylaxis groups.	
Incidence of breakthrough urinary tract infection in hospitalized infants receiving antibiotic prophylaxis<br>(Lloyd et al, 2016)	Observational retrospective cohort study<br><br>Mean follow-up: 10 days	631 infants discharged from 322 NICUs managed by the Pediatric Medical Group from 1997 to 2010, with a previous positive urine culture and treated with a course of prophylactic antibiotics.	Antibiotics considered as potential agents for prophylaxis included amoxicillin, cephalexin, nitrofurantoin, and trimethoprim-sulfamethoxazole.	Risk factors for UTI recurrence.	Breakthrough UTIs (BUTIs) occurred in 60/631 (9.5%) infants.<br><br>The median duration of all prophylactic courses was 10 days (5-20). Amoxicillin was the most commonly used prophylactic antibiotic (549/821, 67%), followed by trimethoprim/ sulfamethoxazole (170/821, 21%), cephalexin (89/821, 11%), and nitrofurantoin (13/821, 2%).	NOS: 7

					Of these prophylactic courses, 65 were complicated by a BUTI (7.9%).<br><br>Trimethoprim/sulfamethoxazole was associated with the lowest incidence of BUTI (5.9%, 10/170). The incidence of BUTI was 7.7% for nitrofurantoin (1/13), 8.0% for amoxicillin (44/549), and 11.2% for cephalexin (10/89). These differences did not reach statistical significance with the $\chi 2$ test (P = .51), nor was any single antibiotic seen to be more effective in preventing BUTI than any other when examined in a multivariable time-to-event analysis based on days of antibiotic given.	
Comparison of cotrimoxazole vs. second-generation cephalosporins for prevention of urinary tract infections in children. (Antachopoulos et al, 2016)	Randomized crossover clinical trial.<br><br>Mean follow-up: 10 months	97 children (44 % female) aged <4.5 years (mean age of 13.6 months) who were hospitalized in a single center in Greece for their first episode of febrile UTI and considered to be eligible for prophylaxis by the treating physicians.<br><br>Exclusion criteria for the study were glucose- 6-phosphate dehydrogenase deficiency, history of allergy to study drugs, congenital or acquired immunodeficiency and/ or already on prophylactic antimicrobial treatment for any reason.	Children recruited were randomized at a 1:1 ratio to receive either trimethoprim-sulfamethoxazole (SXT) or second generation-cephalosporins (2GC: cefuroxime axetil, cefprozil or cefaclor) as prophylaxis for UTI. All patients received both antimicrobial classes interchangeably for periods of 6 months. More specifically, patients initially assigned to receive SXT prophylaxis were switched after 6 months of treatment to 2GC and vice versa.<br><br>69 children received one course of antimicrobial prophylaxis, 15	Risk of breakthrough UTI.	Breakthrough UTIs occurred in 13.3 % (10/75) and 10.3 % (8/78) of children on SXT and 2GC prophylaxis courses, respectively (p = 0.62).<br><br>2GC failures occurred earlier than SXT failures (mean ± standard error: 0.81 ± 0.1 vs. 2.37 ± 0.36 months, respectively; p = 0.028).<br><br>At the end of the SXT courses 38 (92.6 %) isolates were resistant to SXT, while at the end of the 2GC courses 49 (92.4 %) isolates were resistant to 2GC. Based on comparison of the susceptibility patterns of microorganisms isolated before (urine culture confirming UTI) and after the first 6-month prophylaxis course (UOC), the administration of SXT significantly increased resistance to SXT (p = 0.0007) but not to 2GC (p = 0.35), whereas administration of 2GC significantly increased resistance to both SXT (p = 0.027) and 2GC (p = 0.0094)	RoB2 for crossover trials:<br>-Risk of UTI recurrence : some concerns

			received two courses, seven received three courses and six received four or more courses.			

Figure S13. Risk of bias 2 results for randomized clinical trials investigating the risk of UTI recurrence.

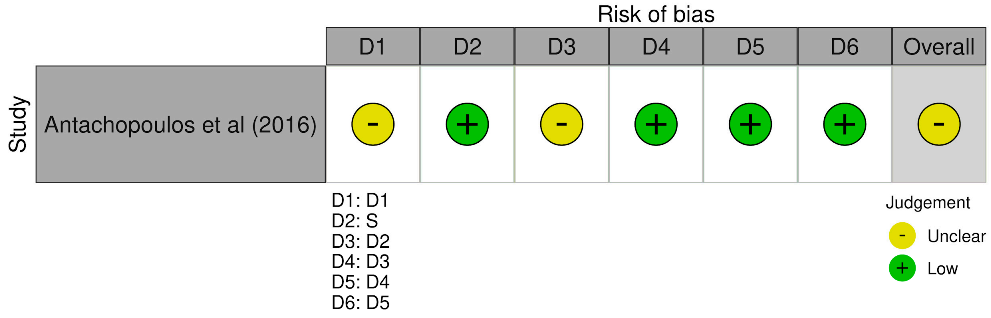

Table S23. Characteristics and results of included systematic reviews

TITLE	POPULATION, INTERVENTIONS AND OUTCOMES	QUALITY ASSESSMENT (AMSTAR2)	RESULTS	CONCLUSIONS
Urinary tract infection in children: recurrent infections (Larcombe et al, 2013)	Two systematic reviews and one RCT were included in this updated systematic review.<br><br>- A: The first systematic review included studies if the majority of children (>50%) did not have a renal tract abnormality, or a major neurological, urological, or muscular disease. It included six RCTs (1069 children) comparing prophylactic antibiotics with placebo or no treatment.<br><br>- B: The second systematic review included only children with primary VUR. It involved eight RCTs (1039 children), six comparing antibiotic prophylaxis with no treatment and two versus placebo.	Critically low	- Nitrofurantoin having a significantly lower risk of resistance than cotrimoxazole (RR 0.54, 95% CI 0.31 to 0.92). Patients receiving nitrofurantoin were twice as likely to experience side effects (nausea, vomiting, or stomachache) as patients receiving trimethoprim (RR 2.18, 95% CI 1.39 to 3.41; RD 33%, 95% CI 17 to 50).	- Nitrofurantoin is less liable to cause resistance, which may account for its superior effectiveness; unfortunately, this is balanced by more adverse effects and treatment dropouts.<br><br>- No systematic review or RCT evidence comparing different durations of antibiotics.

	- C: One RCT, involving 176 children with spina bifida undergoing clean intermittent catheterisation and continuing or discontinuing low-dose prophylactic antibiotics.			
Long-term antibiotics for preventing recurrent urinary tract infection in children.<br>(Williams et al, 2019)	In this Cochrane updated systematic review, 16 studies (2036 children randomized in clinical trials and 1977 not randomized) were included and .<br><br>Children less than 18 years of age who were at risk of recurrence due to prior infection were included. Studies were included if the majority of participants (> 50%) did not have a predisposing cause such as a renal tract abnormality, including VUR, or a major neurological, urological or muscular disease.<br><br>To assess whether long-term antibiotic prophylaxis was more effective than placebo/no treatment in preventing recurrence of UTI in children, and if so which antibiotic in clinical use was the most effective.	High	- It is reported microbial resistance data and analysis showed that treatment with nitrofurantoin may lead to a lower risk of a UTI caused by a bacteria resistant to the treatment drug compared to children given trimethoprim-sulfamethoxazole (RR 0.54, 95% CI 0.31 to 0.92).<br><br>- However, patients receiving nitrofurantoin were twice as likely to experience side effects than patients receiving trimethoprim (RR 2.18, 95% CI 1.39 to 3.41; RD 33%, 95% CI 17 to 50). This suggests that the side effects of nitrofurantoin (NNH = 3, 95% CI 2 to 6) are similar to the prophylactic benefit (NNT = 5, 95% CI 3 to 33) compared with trimethoprim.<br><br>One study compared three antibiotics (cotrimoxazole, cefadroxil and cefprozil) with cefadroxil appearing the most effective. No results showed a difference and the study was underpowered.	- Although nitrofurantoin was more effective than trimethoprim or cotrimoxazole in preventing repeat symptomatic infection or repeat positive urine culture, it was associated with a greater number of side effects. The harmful effects of nitrofurantoin outweigh the prophylactic benefit and suggest that nitrofurantoin may not be an acceptable therapy. Patient compliance would be an important factor to consider in deciding on the use of nitrofurantoin as prophylaxis.

Table S24. Characteristics and recommendations of included guidelines

GUIDELINES			RECOMMENDATIONS	GRADING
2019	Updated Italian recommendations for the diagnosis, treatment and follow-up of the first febrile urinary tract infection in young children	Italian society for pediatric nephrology	a. As a first-choice prophylactic agent, we suggest amoxicillin-clavulanic acid, while ceftibuten or nitrofurantoin should be regarded as secondary options, keeping in mind that nitrofurantoin may cause gastrointestinal intolerance and is inactive against most strains of Proteus.	a. Not specified

After a breakthrough UTI in children already on prophylaxis, which antibiotic should be preferred to continue the prophylaxis?
P: children aged <18 years on antibiotic prophylaxis who experience a breakthrough UTI
I: to continue with the same antibiotic
C: to change with a different antibiotic
O1: risk of UTI recurrence
O2: risk of new renal scars
O3: risk of new antimicrobial resistances
O4: risk of drug-related adverse events

Table S25. Characteristics and results of included studies

TITLE	DESIGN	POPULATION	INTERVENTIONS	OUTCOMES	RESULTS	RISK OF BIAS ASSESSMENT (RoB2 or NOS)
Optimal management of continuous antibiotic prophylaxis after initial breakthrough uti in children with vesicoureteral reflux (Shish, 2021)	Observational retrospective cross-sectional study<br><br>Mean follow-up: 3.5 years	62 patients aged <18 months receiving antibiotic prophylaxis for primary VUR and with a subsequent breakthrough UTI. Children were enrolled in a single center in the USA.	24 (38.7%) had antibiotic prophylaxis changed after a subsequent UTI and 38 had prophylaxis unchanged.	Risk factors for subsequent breakthrough UTI.	A second BT-UTI developed in 12/24 children (50%) with CAP changed and in 22/38 children (57.9%) with CAP unchanged. The relative risk of a second BT-UTI when CAP was changed (versus unchanged) was 0.86 (p 0.55), not statistically significant.	NOS 6

Which dosage should be preferred for long-term antibiotic prophylaxis?
P: children aged <18 years at risk of UTI
I: ⅓ of the standard dosage
C: ½ of the standard dosage
O1: risk of UTI recurrence
O2: risk of new renal scars
O3: risk of new antimicrobial resistances
O4: risk of drug-related adverse events

No studies were included.

Table S26. Characteristics and results of included systematic reviews.

TITLE	POPULATION, INTERVENTIONS AND OUTCOMES	QUALITY ASSESSMENT (AMSTAR2)	RESULTS	CONCLUSIONS
Long-term antibiotics for preventing recurrent urinary tract infection in children. (Williams et al, 2019)	In this Cochrane updated systematic review, 16 studies (2036 children randomized in clinical trials and 1977 not randomized) were included and .<br><br>Children less than 18 years of age who were at risk of recurrence due to prior infection were included. Studies were included if the majority of participants (> 50%) did not have a predisposing cause such as a renal tract abnormality, including VUR, or a major neurological, urological or muscular disease.<br><br>To assess whether long-term antibiotic prophylaxis was more effective than placebo/no treatment in preventing recurrence of UTI in children, and if so which antibiotic in clinical use was the most effective.	High	- Comparing every night cefadroxil treatment with alternate evening therapy, no difference between the doses was evident (RR 0.9, 95% CI 0.24 to 3.41; RD -2%, 95% CI -30 to 26).	- No conclusions were reported about antibiotic dosages.

Table S27. Characteristics and recommendations of included guidelines

GUIDELINES			RECOMMENDATIONS	GRADING
2019	Updated Italian recommendations for the diagnosis, treatment and follow-up of the first febrile urinary tract infection in young children	Italian society for pediatric nephrology	a. There is insufficient evidence to recommend a specific dose; however, traditionally, the dose used for prophylaxis has been one-quarter to one-third of the treatment dose, given once per day. There are no data on the efficacy of the practice of alternating prophylactic antibiotics.<br>b. the optimal duration of prophylaxis has not been established. According to the longer susceptibility to UTI in girls than in boys, we suggest 12-24 months in girls and 6-12 months in boys.	a. Not specified<br>b. Grade C
2015	KHA-CARI guideline: Diagnosis and treatment of urinary tract infection in children	KHA/CARI	a. There is no data that determine the appropriate duration of antibiotic prophylaxis. Most studies have administered prophylaxis for 6 months to 2 years.<br>b. For those children offered prophylaxis, based on results of the PRIVENT and RIVUR trials the following dose and duration is considered appropriate:<br>- 6 months of cotrimoxazole at a dose of 2 mg of trimethoprim plus 10 mg of sulphamethoxazole per kilogram of body weight per day or<br>- 0.25 mL of suspension (containing 40 mg of trimethoprim and 200 mg of sulphamethoxazole per 5 mL) per kilogram to the nearest 0.5 mL.	a. ungraded<br>b. ungraded

How long should antibiotic prophylaxis be continued in children undergoing pyeloplasty?
P: children aged <18 years who underwent pyeloplasty
I: to continue prophylaxis after pyeloplasty
C: to discontinue prophylaxis after pyeloplasty
O1: risk of UTI recurrence
O2: risk of new renal scars
O3: risk of new antimicrobial resistances
O4: risk of drug-related adverse events

Table S28. Characteristics and results of included studies

TITLE	DESIGN	POPULATION	INTERVENTIONS	OUTCOMES	RESULTS	RISK OF BIAS ASSESSMENT (RoB2 or NOS)
Pyeloplasty with ureteral stent placement in children: Do prophylactic antibiotics serve a purpose? (Vidovic et al, 2022)	Observational retrospective cohort study<br><br>Mean follow-up: 4 years	672 patients aged <22 years undergoing pyeloplasty for ureteropelvic junction obstruction between January 2010 and July 2018 across seven institutions in the USA.<br><br>Exclusion criteria were: age older than 22 years, no stent placed, externalized stents used, and incomplete records.	338 received antibiotic prophylaxis after pyeloplasty and 334 did not.<br><br>These groups differed in mean age (3.91 vs. 6.91 years, P < .001), mean stent duration (38.5 vs. 35.32 days, P < .001), and surgical approach (53.25% vs. 32.04% open vs. laparoscopic, P < .001).	Risk factors for subsequent breakthrough UTI.	The incidence of stent UTI was low overall (7.59%) and similar in both groups: 31/338 (9.17%) in the prophylaxis group and 20/334 (5.99%) in the non-prophylaxis group (P = 0.119).<br><br>Although female gender, likely diaper use, and positive intraoperative urine culture were each associated with significantly higher odds of stent UTI, prophylactic antibiotic use was not associated with significant reduction in stent UTI in any of these groups. Surgical approach, stent duration, and Foley duration were not associated with stent UTI.	NOS 7
The role of prophylactic antibiotics after minimally invasive pyeloplasty with ureteral stent placement in children. (Ferroni et al, 2015)	Observational retrospective cohort study<br><br>Mean follow-up: 4 years	163 patients who underwent minimally invasive pyeloplasty from January 2009 to March 2015 in a single center in the USA. All patients had an indwelling urethral catheter placed at the time	Patients were discharged home either with or without a prescription for prophylactic dose trimethoprim-sulfamathoxazole from the time of discharge		Groups were different with respect to median age (7.1 vs 12.0 years, P = .03) and median duration of ureteral stent (35 days vs 28 days, P = .02).<br><br>The incidence of culture-positive UTI between the time of discharge and stent removal was comparably low between	NOS: 7

		of surgery, which was removed on postoperative day one unless otherwise indicated.<br><br>Exclusion criteria included age less than 12 months and greater than 18 years at time of surgery, patients with a positive preop-erative urine culture, patients who were receiving antibiotics (either continuous prophylaxis or active treatment for infec-tion) at time of surgery, cases in which a ureteral stent was not placed due to surgeon discretion, and any case that required an open conversion	until 3 days after ureteral stent<br>removal.<br>Of 163 patients (106 robotic and 57 pure laparoscopic) performed over the study period, 126 patients were discharged on prophylactic antibiotics whereas 37 patients were discharged without prophylaxis.		groups; 2/126 (1.6%) in the prophylaxis group and 1/37 (2.7%) in the group not on prophylaxis.<br><br>At time of stent removal, perioperative urine culture was positive in 2/20 (10.0 %) patients who received prophylactic antibiotics and in 1/25 (4.0%) patients who did not (P = 0.54).	
Urinary tract infection after robot-assisted laparoscopic pyeloplasty: are urine cultures and antibiotics helpful?<br>(Chan et al, 2020	Observational retrospective cohort study<br><br>Mean follow-up: 2 months	152 patients aged <18 years undergoing robot assisted laparoscopic pyeloplasty for ureteropelvic obstruction at a single institution in the USA from 2014 to 2018.<br><br>Patients with vesicoureteral reflux, neurogenic bladder, intermittent catheterization, or < 2 months follow-up after stent removal were excluded.	56 patients received prophylactic antibiotics upon hospital discharge, 27 received therapeutic antibiotics upon hospital discharge and 69 patients did not receive antibiotics.	Risk of UTI recurrence.	UTI occurred in 4/70 (6%) not in post-surgery prophylaxis and 3/56 (5%) in prophylaxis (p=0.92)<br><br>Use of pre-operative prophylactic antibiotics was associated with higher rates of post-RALP UTI (p<0.01).<br><br>Use of post-RALP prophylactic antibiotics was not associated with lower rates of post-RALP UTI (p=0.9).	NOS: 7

How long should antibiotic prophylaxis be continued in children undergoing ablation of posterior urethral valves?
P: children aged <18 years who underwent surgery for posterior urethral valves
I: to continue prophylaxis after surgery
C: to discontinue prophylaxis after surgery
O1: risk of UTI recurrence
O2: risk of new renal scars
O3: risk of new antimicrobial resistances
O4: risk of drug-related adverse events
No studies, systematic review, and guidelines were included.

How long should antibiotic prophylaxis be continued in children undergoing ureteral reimplantation?
P: children aged <18 years who underwent ureteral reimplantation
I: to continue prophylaxis after surgery
C: to discontinue prophylaxis after surgery
O1: risk of UTI recurrence
O2: risk of new renal scars
O3: risk of new antimicrobial resistances
O4: risk of drug-related adverse events
No studies, systematic review, and guidelines were included.

How long should antibiotic prophylaxis be continued in children undergoing endoscopic treatment of VUR?
P: children aged <18 years who underwent endoscopic treatment of VUR
I: to continue prophylaxis after endoscopy
C: to discontinue prophylaxis after endoscopy
O1: risk of UTI recurrence
O2: risk of new renal scars
O3: risk of new antimicrobial resistances
O4: risk of drug-related adverse events
No studies, systematic review, and guidelines were included.

Table S29. AGREE II domain scores for included guidelines.

GUIDELINES	DOMAIN					
	SCOPE AND PURPOSE	STAKEHOLDER INVOLVEMENT	RIGOUR OF DEVELOPMENT	CLARITY OF PRESENTATION	APPLICABILITY	EDITORIAL INDEPENDENCE
SINePe (2019) [32]	100%	67%	43%	91%	70%	100%
Asian guidelines (2021) [52]	48%	55%	32%	64%	26%	50%
Swiss consensus (2020) [33]	81%	64%	56%	86%	70%	66%
NICE (2018) [37]	100%	72%	65%	94%	74%	100%
EAU/ESPU (2021) [38]	100%	75%	75%	94%	78%	83%
KHA-CARI (2015) [34]	100%	55%	76%	91%	78%	83%
AUA (2010) [39]	89%	72%	78%	78%	56%	83%
AAP (2011) [49]	100%	92%	97%	94%	89%	83%
